# Automated segmentation of target volumes in breast cancer radiotherapy, impact on target size and dose to organs at risk

**DOI:** 10.1016/j.ctro.2025.100986

**Published:** 2025-05-28

**Authors:** Vivi Tang, Elinore Wieslander, Mahnaz Haghanegi, Elisabeth Kjellén, Sara Alkner

**Affiliations:** a Skåne University Hospital, Department of Hematology, Oncology and Radiation Physics, 222 42 Lund, Sweden; b Lund University, Faculty of Medicine, Institute of Clinical Sciences, Department of Oncology, Barngatan 4, 22242 Lund, Sweden

**Keywords:** Deep learning segmentation, AI contouring, Target volume delineation, Dosimetric data, Radiotherapy, Breast cancer

## Abstract

**Introduction:**

Target volume delineation is crucial in breast cancer radiotherapy planning but involves significant interobserver variability. Deep learning (DL) models may reduce this variability, saving time and costs. However, current DL-models do not consider clinical data, such as tumor location and patient comorbidity, to adjust the target and reduce dose to organs at risk (OAR). This study compares clinically defined target volumes to those generated by a DL-model in terms of size, geometric overlap, and dose to OAR.

**Method:**

For a hypothetical breast cancer patient, we compared target volumes constructed by Swedish radiotherapy clinics and two DL-models, Raystation and MVision. Geometrical overlap was evaluated, as well as the impact of differences in target delineation on dose to OAR. Treatment plans for locoregional vs. breast-only 3D-conformal radiotherapy were generated.

**Results:**

CTV-structures for the breast, lymph nodes level I-IV, and internal mammary nodes were available for 10, 11, and 14 centers respectively. Volume of the CTV-breasts varied between 770–890cc, and the total CTV-volumes (breast + lymph nodes) between 875–1003cc. The DL-models did not constitute the largest nor smallest breast or total CTV-volumes, and geometric overlap between structures was relatively good. Evaluating dose to OAR from dose plans based on the respective CTV-volumes for locoregional radiotherapy, this was comparable between the DL-models and the mean of the CTVs generated by the clinics. In radiotherapy of only the breast, the CTV-breasts constructed by the DL-models gave the highest heart doses due to their proximity to the chest wall, affecting field angle choices. No difference was seen in dose to the ipsilateral lung, thyroid gland, or humeral head.

**Conclusion:**

DL-models for target delineation have great potential. However, their introduction must be closely monitored since even small differences compared to clinical standards may affect doses to OAR in 3D conformal breast cancer radiotherapy.

## Highlights

• This study compares target volumes from 15 clinics and 2 AI models.
• AI models’ target volumes were neither largest nor smallest.
• Geometric overlap was good between AI models and clinically constructed CTVs.
• AI models’ CTVs gave the highest heart doses in whole breast radiotherapy.

## 1 Introduction

The complex process of planning radiotherapy is accomplished by an expert team. Adjuvant breast cancer target delineation has historically been performed by a radiation oncologist, taking contouring guidelines as well as patient and tumor related information into consideration. With AI (artificial intelligence) being a growing aid globally including in radiotherapy, several studies have shown its efficacy in reducing time consumption and interobserver variability [[1], [2], [3]], by automatizing target and organ at risk (OAR) delineations [4,5]. However, current deep learning (DL) based auto-segmentation models solely rely on CT (computed tomography) imaging for contouring, not adjusting the target volume in accordance with the patient’s clinical data. This has led to concerns that using a DL-model will generally result in larger target volumes and higher doses to adjacent OAR such as the heart and lungs. In addition, in case of a rare tumor location, parts of the tumor bed may not be included in the DL-model generated target volume.

Numerous studies have evaluated geometric concordance between clinical defined targets and those generated by a DL-model [[6], [7], [8], [9], [10]], and generally shown this to be acceptable. However, due to the tangential field technique most often used in breast cancer radiotherapy, specific regions of the target volume such as the lateral and medial border of the breast, are crucial in determining dose to the lung and the heart. Hence, only comparing overlap between two target volumes does not answer the question as to how generating a dose plan based directly on a DL-model based target will affect dose to OAR.

We therefore conducted this study with data from the Swedish Radiotherapy Group, where 15 Swedish radiotherapy clinics have defined target volumes (breast and regional lymph node stations) for the same hypothetical breast cancer patient. Target volumes, including dose plans made for each target, are compared between the respective clinics and the two most commonly used commercially available DL based models. Our aim was to assess whether it is safe to rely on the DL-models in clinical practice by comparing clinically defined target volumes to those generated by a DL-model in terms of size and geometrical overlap. We also evaluate how dose plans based on the respective target volumes differ in dose to OAR, and how using a dose plan constructed from a DL-model based target would affect target coverage and dose outside target compared to the respective clinics current standard.

## 2 Materials and methods

This study was approved by Swedish Ethic Review Authority (Dnr 2023–02667-01). In 2023 the Swedish Breast Radiotherapy Group performed a dummy run, where Swedish radiotherapy clinics delineated target for the same fictive breast cancer patient. The participating clinics were instructed to outline Clinical Target Volume (CTV) and/or Planning Target Volume (PTV) according to local routine based on either European Society of Radiation Oncology (ESTRO) guidelines [11] or the previous Swedish target guidelines [12] (Supplementary Information 1 and 2).

The CT-scan chosen was from a former breast cancer patient treated at Skåne University Hospital, representing a patient with “normal” anatomy in relation to body constitution, lung volume and position of the heart. A short fictive medical history was provided, telling this to be a patient with a left sided T2 ductal, luminal breast cancer with a median location in the breast, 1 sentinel node macrometastasis, operated with breast conserving surgery and sentinel node biopsy (no axillary dissection). It was further stated that the patient should receive 40.05 Gy in 15 fractions to the remaining breast and lymph nodes level I-IV, interpectoral nodes and the internal mammary nodes (IMN).

Fifteen out of sixteen Swedish radiotherapy clinics participated in the dummy, and target was delineated by one of the clinics radiation/clinical oncologists. OAR was delineated by a radiation oncologist in accordance with current clinical standards. However, the lungs and the body contour were generated by the Eclipse software auto-contouring tool (version 15.6, Varian Medical Systems, Palo Alto, CA, USA) and then manually adjusted if needed.

One clinic used uncorrected DL-based delineation for target definition, hence this clinic was excluded from further analysis below. CTV-IMN was available for all remaining 14 clinics. In relation to the breast and lymph nodes level I-IV and the interpectoral nodes, two centers delineated these as one joint PTV directly (no CTV), in accordance with the previous Swedish guidelines. In addition, two centers delineated PTV for the breast directly, but CTV for the lymph nodes. One of these clinics did not delineate level I. Since only CTV-structures were possible to compare to the DL-models delineations, this left 10 centers with CTV-breast delineated, and 11 with CTV lymph nodes level I-IV and the interpectoral nodes delineated.

Along with delineations by the sites’ radiation oncologists, target was also defined by two commercially available DL-models for target delineation, MVision (version 1.2.3, MVision AI, Helsinki, Finland) (MV-DL) and Raystation (version 12A, RaySearch Laboratories AB, Sweden) (RS-DL) [6] (further information at: https://mvision.ai/contour/ and https://www.raysearchlabs.com). Following CTVs were contoured: residual breast, lymph node level 1–4, interpectoral nodes and IMN. The CTVs for lymph node level I-IV and the interpectoral nodes were grouped and analyzed together as CTVN, while the CTV-breast and CTV-IMN were analyzed as separate volumes. OAR included ipsilateral lung, contralateral lung, contralateral breast, esophagus, thyroid gland, heart, humeral head and body.

As a golden standard for target structure delineation CTV-mean-structures were created from all the participating centers CTVs’. The mean structure is based on the mean value, in each point, of the summed structure masks and points with values ≥0.5 are assigned to the mean structure. (Hero version 2024.2.0, Hero Imaging AB, Sweden). PTV was created with a 5 mm margin around CTV-breast, CTVN, and CTV-IMN.

### 2.1 Data analysis

Volume data was obtained through software Eclipse (version 18.0, Varian Medical Systems, Palo Alto, CA, USA). For comparison of target delineations both 2D measurements, and 3D volumes were evaluated. 2D measurements were focused on the cranial, caudal, medial and lateral border definition of the breast, the cranial border of level IV, and the caudal border of level I and IMN.

3D-conformal dose plans for locoregional treatment, i.e CTV-breast + CTVN + CTV-IMN, and whole breast radiotherapy plans, i.e CTV-breast, were generated by an experienced dose planner based on contouring from RS-DL and MV-DL, the CTV-mean-structure, the hospitals with the largest (Center J) vs. smallest CTV-breast volume (Center A), and the hospitals with the largest (Center H) vs. smallest total CTV volume (Center C), defined as the total volume in cubic centimeters (cc) for CTV-breast + CTVN + CTV-IMN. The field setup was tangential for CTV-breast and anteroposterior/posteroanterior for CTVN. The treatment planning system used was Eclipse version 18.0 (Varian Medical Systems, Palo Alto, CA, USA) and the anisotropic analytical algorithm version 15.6.05. The prescribed dose was 40.05 Gy in 15 fractions, five days a week. The dose distribution to OAR was analyzed using the Swedish national guidelines for breast cancer radiotherapy, as in clinical routine (Supplementary Table 1). I.e doses given below are in absolute numbers. Treated and irradiated volume were analyzed according to ICRU report 50 [13]. The treated volume was defined as the volume receiving 90 % of the prescribed dose, and the irradiated volume as the volume receiving 50 % of the prescribed dose.

The degree of target coverage and dose outside PTV were evaluated when applying the dose plans based on RS-DL and MV-DL models’ target definitions upon the 10 clinics with CTV available for all structures and the CTV-mean. The analyze was performed according to Swedish national guidelines recommending CTV-breast + CTVN receiving D98% ≥95 %, CTV-IMN D98% ≥90 %, and PTV-breast + PTVN D98% ≥93 % (Supplementary Table 1).

Coherence between two delineations were assessed through surface dice similarity coefficient (sDSC) [14,15], with the equivalent CTV-mean-structure as reference. The sDSC measure evaluates the overlap of two surfaces with a predefined tolerance for differences, in our case 3 mm and 5 mm. A surface dice similarity coefficient with value 0 indicates no overlap and a value of 1 indicates complete overlap. sDSC were calculated in Hero (version 2024.2.0, Hero Imaging AB). Mean value and medial value for sDSC in respective structure group were analyzed in Microsoft Excel (version 16.24, Microsoft Corporation).

## 3 Results

### 3.1 CTV-breast

**Fig. 1:**
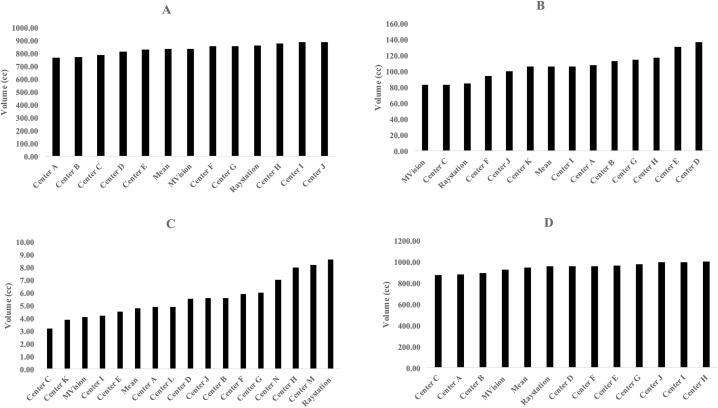
**A-d.** Variability in target volume in CTV-breast (A), CTV lymph node level I-IV including the interpectoral nodes (CTVN) (B), CTV internal mammary chain (IMN) (C) and CTV-breast + CTVN + CTV-IMN (D).

**Fig. 2:**
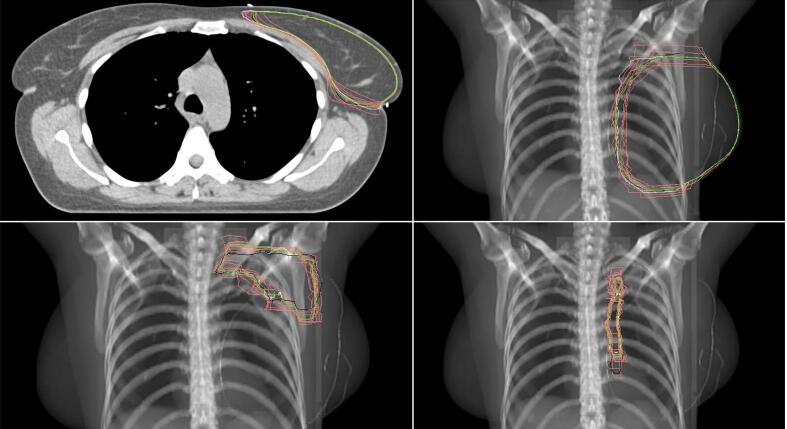
Axial CT image and topogram showing CTV breast, CTVN and CTV-IMN for all included centers. Raystation’s CTV is black, MVision’s CTV white and the Mean-CTV-structure green. (For interpretation of the references to colour in this figure legend, the reader is referred to the web version of this article.)

CTV-breast was available for 10 clinics and the two DL-models. For the structures outlined by the clinics the CTV-volume ranged between 770 and 890cc. RS-DL outlined 861cc and MV-DL outlined 838cc (Fig. 1a). The volume for the CTV-breast-mean structure was 835cc. Delineation difference was visually evaluated and measured considering the medial, lateral, cranial and caudal edges (Fig. 2). The maximal differences in cranial/caudal/lateral/medial borders between clinics including the DL-models were 2.70/1.50/1.75/2.10 cm respectively (Supplementary Table 2). The two DL-models constituted neither the inner nor outer limits in either parameter.

### 3.2 CTVN: Lymph node level I-IV and the interpectoral nodes

CTVN, including lymph node level I-IV and the interpectoral nodes, were available for 11 clinics and the two DL-models. Volume ranged between 83 and 137cc for the structures outlined by the clinics. RS-DL outlined 85cc, and MV-DL 83cc which is less than many of the clinics (Fig. 1b). The CTVN-mean volume was 106cc. The largest cranial border discrepancy for level IV i.e. the CT-section at which delineation of CTVN began was 1.80 cm. The largest difference in caudal border of level I i.e. the most caudal part of CTVN, was also 1.80 cm. In both cases RS-DL represented the innermost border (Fig. 2, Supplementary Table 2).

### 3.3 CTV-IMN

CTV-IMN was available for 14 clinics and the two DL-models. Volume ranged between 3.20 to 8.20cc for the structures outlined by the clinics. MV-DL CTV-IMN volume was 4.10cc while RS-DL CTV-IMN represented the largest IMN-volume of 8.60cc, almost double the volume of the CTV-IMN-mean 4,80cc (Fig. 1c). However, RS-DL CTV-IMN did not have the most caudal border of the CTV-IMNs (Fig. 2). The caudal border of CTV-IMN differed 5.70 cm between the clinics that delineated the shortest vs. the longest CTV-IMN (Supplementary Table 2).

### 3.4 Total CTV-volume

Total volumes for CTV breast, CTVN and CTV-IMN were assessed for the 10 clinics with CTV available for all structures and the two DL-models (875-1003cc). RS-DL and the CTV-mean-volume showed similar target size with 955cc and 946cc respectively. MV-DL outlined a somewhat smaller volume of 925cc (Fig. 1d).

### 3.5 Delineation coherence – Surface dice similarity coefficient

There is no consensus as to what constitutes a significant threshold value for a high sDSC [14]. Within tolerance 3 mm, our data showed greatest overall concordance in the CTV-breast with a mean value 0.94 (0.89–0.99). Bigger coherence variability was seen for CTVN (0.76–0.90) and CTV-IMN (0.77–1.00). However, with a tolerance of 5 mm the consistency was considered overall good with mean sDSC > 0.90 for CTV-breast, CTVN and CTV-IMN (Table 1).

**Table 1:** Surface dice similarity coefficient (sDSC) in relation to the Mean-CTV-delineation for the corresponding structures^1^.

	Surface Dice 3 mm	Surface Dice 5 mm
**CTV-breast**		
Center A	0.91	0.97
Center B	0.89	0.95
Center C	0.91	0.95
Center D	0.93	0.98
Center E	0.99	1.00
Center F	0.97	0.99
Center G	0.95	0.98
Center H	0.93	0.96
Center I	0.91	0.96
Center J	0.92	0.95
Raystation	0.99	1.00
MVision	0.97	0.99
Mean value	0.94	0.97
Median value	0.93	0.97

**CTVN**		
Center A	0.85	0.93
Center B	0.83	0.93
Center C	0.89	0.95
Center D	0.76	0.91
Center E	0.77	0.91
Center F	0.90	0.96
Center G	0.89	0.95
Center H	0.87	0.95
Center I	0.88	0.94
Center J	0.85	0.95
Center K	0.82	0.93
Raystation	0.86	0.92
MVision	0.83	0.91
Mean value	0.85	0.93
Median value	0.85	0.93

**CTV-IMN**		
Center A	0.97	0.98
Center B	0.93	0.94
Center C	0.88	0.90
Center D	0.91	0.97
Center E	0.87	0.89
Center F	0.87	0.92
Center G	0.95	0.97
Center H	0.91	0.94
Center I	0.95	0.98
Center J	0.77	0.85
Center K	0.96	0.99
Center L	1.00	1.00
Center M	0.84	0.92
Center N	1.00	1.00
Raystation	0.91	0.93
MVision	0.89	0.90
Mean value	0.91	0.94
Median value	0.91	0.94

^1^Surface dice values with overlap tolerance 3 mm respectively 5 mm between the different target delineation surfaces.

### 3.6 Difference in dose to organs at risk in relation to target definition

In the analysis of dose plans for locoregional treatment of the CTV-breast, CTVN and CTV-IMN, mean heart dose ranged between 1.95 to 3.95 Gy, with dose plans based on the DL-models giving a mean heart dose of 2.84 Gy and 3.03 Gy respectively. Mean dose to ipsilateral lung ranged between 12.10 and 14.15 Gy, with the DL-models giving doses of 13.00 Gy and 12.70 Gy respectively. Mean dose to thyroid gland varied between 13.33 Gy to 17.61 Gy and dose plans based on our DL-models contributed with the lowest doses, 13.33 Gy and 13.40 Gy respectively. A variation was further seen in mean dose to humeral head with a range of 6.31 Gy to 11.49 Gy, and the radiation exposure with the DL-models dose plans were 10.04 Gy and 11.12 Gy respectively (Table 2a).

**Table 2a:** Dose to organs at risk based on locoregional radiotherapy plans for CTV-breast + CTVN + CTV-IMN.

	Constraint	Center A (smallest CTV-breast volume)	Center C (smallest total CTV volume)	Center H (largest total CTV volume)	Center J (largest CTV breast- volume)	CTV-mean	Raystation	MVision
**Heart**	Dmean (Gy)	1.95	2.02	3.95	2.94	2.76	3.03	2.84
	V17Gy (%)	0.99	1.33	6.82	3.79	3.43	4.15	3.89
**Lung** (ipsilateral)	V16Gy (%)	28.85	28.09	34.73	34.40	30.62	30.99	30.23
	Dmean (Gy)	12.19	12.10	14.15	13.93	12.83	13.00	12.70
	V5Gy (%)	51.16	51.05	54.87	54.40	52.20	53.00	52.07
**Lung** (contralateral)	Dmean (Gy)	0.40	0.37	0.62	0.54	0.43	0.43	0.39
**Breast** (contralateral)	Dmean(Gy)	0.16	0.16	0.31	0.25	0.18	0.19	0.16
**Esophagus**	V9Gy (cc)	0.81	0.03	0.84	0.12	0.05	0.80	0.79
	V18Gy (cc)	0.32	0.00	0.33	0.00	0.00	0.32	0.32
	Dmean (Gy)	1.60	1.01	1.75	1.16	1.06	1.56	1.55
**Thyroid gland**	Dmean (Gy)	16.32	15.18	17.61	16.07	15.87	13.40	13.33
**Body**	V105% (cc)	79.74	72.71	112.50	128.43	85.60	139.11	143.66
**Humeral Head**	Dmean (Gy)	11.49	6.31	11.74	8.70	8.95	10.04	11.12

Abbreviations: *cc* cubic centimeters, *CTVN* clinical target volume of level I-IV and the interpectoral nodes, *CTV-IMN* clinical target volume of the internal mammary nodes, *D* dose, *Gy* Gray, *V* volume.

**Fig. 3:**
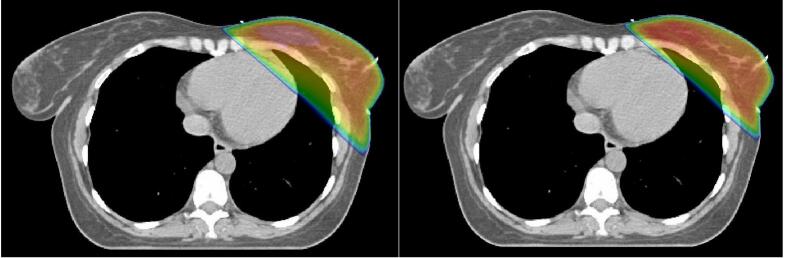
CTV-breast dose plans illustrating the difference in dose to the heart between the delineations contributing with maximum (left picture, RS-DL) respective minimum heart dose (right picture, Center A).

Dose plans for whole breast radiotherapy, i.e. only to the CTV-breast, were analyzed separately. Mean dose to heart ranged between 1.27 Gy to 2.50 Gy with the highest doses seen in the dose plans generated for the DL-models target volumes. Mean dose to heart was 2.50 Gy with RS-DL and 2.19 Gy with MV-DL (Fig. 3). A similar pattern was seen in mean dose to ipsilateral lung which received 6.89 Gy with RS-DL CTV and 6.78 Gy with MV-DL CTV (range 4.83–7.41 Gy) (Table 2b).

**Table 2b:** Dose to organs at risk based on whole breast radiotherapy plans for CTV-breast.

	Constraint	Center A (smallest CTV-breast volume)	Center C (smallest total CTV volume)	Center H (largest total CTV volume)	Center J (largest CTV- breast volume)	CTV-mean	Raystation	MVision
**Heart**	Dmean (Gy)	1.27	1.79	2.10	1.64	1.81	2.50	2.19
	V17Gy (%)	0.88	2.15	2.66	1.53	2.15	3.94	3.09
**Lung** (ipsilateral)	V16Gy (%)	9.95	11.21	17.37	14.52	13.66	15.86	15.34
	Dmean (Gy)	4.83	5.39	7.41	6.50	6.15	6.89	6.78
	V5Gy (%)	18.43	20.74	26.85	24.84	22.87	25.28	25.18
**Lung** (contralateral)	Dmean (Gy)	0.06	0.07	0.11	0.09	0.08	0.10	0.10
**Breast** (contralateral)	Dmean (Gy)	0.10	0.05	0.07	0.06	0.05	0.06	0.06
**Esophagus**	V9Gy (cc)	0.00	0.00	0.00	0.00	0.00	0.00	0.00
	V18Gy (cc)	0.00	0.00	0.00	0.00	0.00	0.00	0.00
	Dmean (Gy)	0.25	0.29	0.34	0.36	0.29	0.32	0.32
**Thyroid gland**	Dmean (Gy)	0.15	0.19	0.30	0.43	0.20	0.24	0.26
**Body**	V105% (cc)	10.10	18.93	3.19	5.53	4.55	0.66	8.57
**Humeral Head**	Dmean (Gy)	0.44	0.49	1.27	1.64	0.62	0.78	0.71

Abbreviations: *cc* cubic centimeters, *D* dose, *Gy* Gray, *V* volume.

### 3.7 Target coverage and dose to normal tissue when using a DL-based plan on clinically defined target volumes

When applying RS-DL dose plan, CTV-breast + CTVN coverage was fulfilled for all 10 clinical centers delineation. Target coverage was fulfilled for all clinically delineated CTV-IMN as well. PTV-breast + PTVN coverage was fulfilled for seven clinics (Table 3).

**Table 3:** Dose coverage when applying a dose plan based on Raystation’s and MVision’s target definition on other CTV outlines.

	Center A (smallest CTV-breast volume)	Center B	Center C (smallest total CTV volume)	Center D	Center E	Center F	Center G	Center H (largest total CTV volume)	Center I	Center J (Largest CTV-breast volume)	CTV-mean	Raystation	MVision
**Raystation's dose plan**													
**CTV-breast + CTVN** D98% (%)(recommended ≥ 95 %)	95.60	95.50	95.70	95.10	95.50	95.30	95.20	95.10	95.10	95.40	97.80	95.90	96.10
**CTV-IMN** D98% (%) (recommended ≥ 90 %)	90.40	91.30	91.40	93.30	91.00	90.50	91.20	90.70	90.40	91.60	91.40	90.70	95.40
**PTV-breast + PTVN** D98% (%) (recommended ≥ 93 %)	93.00	94.00	93.30	93.20	93.10	91.90	93.10	91.40	91.00	93.00	93.60	94.40	94.40
**PTV-Body** V90% (cc)	1103.23	1144.87	1145.3	1089.19	1064.21	1055.94	1064.21	1021.34	1013.6	1003.17	1078.21	1044.23	1065.18

**Mvision's dose plan**													
**CTV-breast + CTVN** D98%(%) (recommended ≥ 95 %)	95.20	95.20	95.30	94.80	95.20	94.90	94.70	94.70	94.70	95.00	95.20	95.40	95.60
**CTV-IMN** D98% (%) (recommended ≥ 90 %)	87.90	89.50	90.30	92.60	90.10	85.90	89.40	89.00	85.70	90.00	89.90	89.30	96.00
**PTV-breast + PTVN** D98% (%) (recommended ≥ 93 %)	93.10	93.90	93.20	93.90	92.80	91.90	92.80	90.90	90.50	92.60	93.40	93.90	94.00
**PTV-Body** V90% (cc)	1073.13	1113.32	1115.32	1057.70	1035.06	1025.88	1035.06	993.66	986.17	975.04	1048.93	1016.19	1036.47

Abbreviations: *cc* cubic centimeters, *CTVN* clinical target volume of level I-IV and the interpectoral nodes, *CTV-IMN* clinical target volume of the internal mammary nodes, *D* dose, *Gy* Gray, *V* volume.

When analyzing MV-DL dose plan, CTV-breast + CTVN coverage was fulfilled for five out of ten centers. CTV-IMN coverage was fulfilled for four out of ten centers. PTV-breast + PTVN coverage was fulfilled for four out of ten centers (Table 3).

The irradiated volume of normal tissue, here defined as the tissue volume outside PTV receiving >90 % of the prescribed dose, was 1044cc with RS-DL PTV and dose plan. When applying RS-DL dose plan on the PTVs defined by the clinics, the irradiated volume outside PTV was <1044cc for three centers (1003 – 1021 cc), and >1044 cc for seven centers (1056–1145 cc). Indicating more vs. less of the high dose area being defined as target compared with the PTV defined by RayStation. The corresponding volume for MV-DL dose plan was 1036cc. When applying this dose plan to the clinically defined CTVs, the irradiated volume outside PTV was <1036 cc for six centers (975-1035cc) and >1036 cc for four centers (1049–1115 cc) (Table 3).

## 4 Discussion

With AI being a growing aid globally including in radiotherapy, our study presents significant findings considering future implementation of AI for target delineation in clinical practice.

Analysis of volume data revealed the clinical CTV-breast delineations to be both smaller and larger than those delineated by the DL-models. For CTVN (level I-IV and the interpectoral nodes) the DL-models created smaller delineations than the clinics. RS-DL created the largest CTV-IMN volume while MV-DL created the third smallest in terms of volume in cc. Geometrical conformity was assessed through sDSC in reference to a mean-structure. There is no golden standard in creating a “ground truth” structure [16,17] and here we chose to create a mean structure based on included clinics. Greatest overall concordance was seen in CTV-breast with bigger coherence variability for CTVN and CTV-IMN which align with previous studies [6,7,18]. In the study by Almberg et al. the difference in CTV-breast was not significant while the difference in CTVN was [7]. Meixner et al. concluded that the most frequent adjustments were needed in cranial and caudal aspects of the target [6], which coordinates with our finding of maximal variance of the caudal border of CTV-IMN being 5.70 cm between the clinics and our DL CTV-IMNs not being outliers. Volume data and geometrical analysis does indicate whether the DL-delineations are reasonable, but these data do not predict the clinical adequacy since dose to organs at risk depends on margins at certain localizations, which are not shown through these metrics.

Therefore, we further analyzed dose to OARs. Mean dose to the heart ranged between 1.95 Gy to 3.95 Gy and 1.27 Gy to 2.50 Gy in the locoregional and tangential treatment plans respectively. Our DL-models represented doses closer to the upper limit, due to their CTV-breast extending closer to the thoracic wall as well as RS-DL CTV-IMN extending deeper in the thorax than many centers.

In locoregional and whole breast radiotherapy the differences in mean heart dose between the plan with the highest and lowest dose were 2.00 Gy and 1.23 Gy respectively. This difference may not be considered that large measured in Gray. However, they constitute an increase of 103 % and 97 % respectively within the range. In a study by Kügele et al., deep inspiration breath-hold (DIBH) technique compared to free breathing reduced mean heart dose with 0.76 Gy and 0.54 Gy in locoregional and tangential breast radiotherapy respectively [19]. These reductions were considered significant, and breath hold techniques in order to spare the heart, are today implemented at most modern radiotherapy departments. We here show that uncritically implementing a new technique for target delineation, may easily increase heart dose in a similar range as by not using DIBH.

There was no considerable variation in mean dose to ipsilateral lung between CTV-mean and RS-DL and MV-DL in the locoregional treatment plans with irradiation doses of 12.83 Gy, 13.00 Gy and 12.70 Gy respectively. Furthermore, no considerable variations were seen between CTV-mean, RS-DL and MV-DL in the local treatment plan, with irradiation doses of 6.15 Gy, 6.89 Gy and 6.78 Gy. Dose to thyroid gland was lowest in the DL-based locoregional treatment plans as a result of the lower cranial border of CTVN. Dose to the humeral head varied depending on target delineation, but was comparable between the DL-models and the dose plans based on clinically generated target volumes.

Dose coverage was investigated through application of the DL-model based treatment plans on the manually delineated targets. CTV-coverage for breast, lymph nodes level I-IV and IMN were fulfilled for all the clinically defined delineations when applying the RS-DL dose plan. The MV-DL dose plan showed underdosage for five centers for CTV-breast + CTVN and four centers considering CTV-IMN. Our results partially align with previous studies showing overall good CTV-coverage when applying dose plans based on auto-segmented delineations on manual delineations [7,8]. Considerable discrepancies in manual and auto-segmented contours of IMN were seen in the study by Chung et al. that to our understanding assessed dose plans based on manually generated target volumes on both manual and DL-contours [10].

Regarding PTV-coverage, the dose plan based on RS-DL showed acceptable target coverage for 70 % of the clinics while MV-DL’s dose plan fulfilled target coverage for 40 % of the clinics. These findings are consistent with the volume data, showing that RS-DL delineation for total volume i.e. CTV breast, lymph node level I-IV, IMN, was overall bigger than the mean of the clinics structures while MV-DL instead delineated a smaller total volume than the clinical mean. Whether the DL-models’ treatment plans demonstrate adequate overall dose coverage depends not only on the size but also on the shape of the clinic’s delineation. Hence reaching a definitive conclusion on what is correct or incorrect is challenging.

There are certain limitations to our study focusing solely on one fictive patient restricted to the two investigated DL-models, and therefore the results may not be generalized to the total patient population as well as all available DL-models. In addition, results only apply to 3D conformal radiotherapy. However, many clinics participated in this study and hence the data still indicates certain aspects of DL auto-segmentation. To our understanding no previous study has evaluated dose to OARs by comparing DL-based treatment plans to manually based plans and consequently these findings give novel insights to current knowledge.

In this study the DL-models investigated presented high quality delineations similar to those generated by the clinics. To date the DL-models however, do not consider clinical aspects such as breast cancer histological subtype and tumor location. The use of DL-models have been proved to reduce time consumption and interobserver variability [20,21], which is promising but also implies its inability to deviate from guidelines in complex patient cases [20]. To date, we can effectively use the DL-targets followed by target modification of primarily CTVN and CTV-IMN considering sDSC as well as the medial and lateral border of CTV-breast affecting mean dose to heart.

In conclusion, although DL-models have great potential for being a strong support in clinical practice, their introduction in the clinic must be closely monitored since even small differences compared to clinical standards may affect dose to OAR. Before fully relying on the DL-models, software improvement and further studies also need to be conducted with a broader spectrum of breast cancer patient cases.

## Declaration of Generative AI and AI-assisted technologies in the writing process

During the preparation of this work the authors used Microsoft Copilot in order to improve language. After using this tool/service, the authors reviewed and edited the content as needed and takes full responsibility for the content of the publication

## Funding sources

This work was supported by the Swedish Cancer Society, grant number: 22 2015 S. and 21 1889 S. The Swedish Society of Medicine, grant number: SLS-971645. Skåne University Hospital’s Foundations, grant number: Alkner 2022. Mrs Beta Kamprad’s Foundation, grant number: FBKS-2022-6 – 376. Gunnar Nilsson’s Cancer Foundation, grant number: GN-2022-1 – 265. Percy Falk’s Foundation, grant number: 2021-EQX803. The Swedish Society for Medical Research and Governmental Funding of Clinical Research within National Health Service, grant number: 2022-Projekt0008.

The funders have no authority on study design; collection, management, analysis, and interpretation of data; writing of the report; and the decision to submit the report for publication.

## CRediT authorship contribution statement

**Vivi Tang:** Data curation, Formal analysis, Investigation, Methodology, Visualization, Writing – original draft, Writing – review & editing. **Elinore Wieslander:** Conceptualization, Data curation, Formal analysis, Investigation, Software, Supervision, Validation, Visualization, Writing – original draft, Writing – review & editing. **Mahnaz Haghanegi:** Conceptualization, Data curation, Formal analysis, Investigation, Methodology, Supervision, Writing – original draft, Writing – review & editing. **Elisabeth Kjellén:** Conceptualization, Formal analysis, Investigation, Methodology, Project administration, Supervision, Validation, Visualization, Writing – original draft, Writing – review & editing. **Sara Alkner:** Conceptualization, Data curation, Formal analysis, Funding acquisition, Investigation, Methodology, Project administration, Resources, Supervision, Validation, Visualization, Writing – original draft, Writing – review & editing.

## Declaration of Competing Interest

The authors declare that they have no known competing financial interests or personal relationships that could have appeared to influence the work reported in this paper.
