# Supplementary material for: Automated segmentation of target volumes in breast cancer radiotherapy, impact on target size and dose to organs at risk
Source: Clin Transl Radiat Oncol. 2025 May 28;53:100986. doi: 10.1016/j.ctro.2025.100986 (PMC12173629; doi:10.1016/j.ctro.2025.100986)
Supplement: Supplementary Data 1 [file mmc1.pdf]

Supplementary Information

Supplementary Table 1.

Dose-volume criteria for patients receiving locoregional radiotherapy 40.05 Gy / 15F.

Priority	Volume		Constraint
**1**	**CTVT_40**		$D_{99,9\%} \geq 95\%$<br>$D_{mean} \geq 100\%$
	**CTV_40**	*For lobular ≥ T2 or multifocal cancer*	$D_{98\%} \geq 95\%$
	**PTV_40**		$D_{98\%} \geq 93\%$
	**Heart**		$D_{mean} < 4Gy$ (recommended 1,6Gy)<br>$V_{17Gy} \leq 10\%$ (recommended ≤ 5%)
**3**	**Lung_R/L** (ipsilat.)		$V_{16Gy} \leq 35\%$ (recommended 20%)<br>$D_{mean} \leq 16Gy$ (recommended 8Gy)<br>$V_{5Gy} \leq 70\%$ (recommended 55%)
	**Lung_L/R** (contralat.)		$D_{mean} < 2Gy$
**4**	**CTV_40** (breast+lgl)		$D_{98\%} \geq 95\%$
	**PTV_40** (breast+lgl)<br>**PTVT_40**		$D_{98\%} \geq 93\%$<br>$V_{105\%} \leq 20\%$ (recommended 10%)
**6**	**Breast_R/L** (contralat.)		$D_{mean} < 4Gy$ (recommended 2Gy)
**7**	**Esophagus**		$V_{9Gy} < 5cm^3$<br>$V_{18Gy} < 2cm^3$<br>D_{mean} lowest possible
	**Thyroid gland**		$D_{mean} < 20Gy$ (recommended 15Gy)
	**Body**		$V_{110\%}$ should be $< 1cm^3$<br>$V_{105\%}$ lowest possible
**8**	**Humeral Head_R/L**		D_{mean} lowest possible

Supplementary Table 2: Border variance in CTV breast, CTVN and CTV-IMN.

	Variance (cm)
**Maximal variance upper border CTV-breast**	2.70
**Maximal variance lower border CTV-breast**	1.50
**Maximal variance medial border CTV-breast**	2.10
**Maximal variance lateral border CTV-breast**	1.75
**Maximal variance upper border CTVN**	1.80
**Maximal variance lower border CTVN**	1.80
**Maximal variance lower border CTV-IMN**	5.70

Swedish National guidelines for target delineation constructed 2003/2004

PTV:

Corresponds to CTV breast and regional lymph nodes with 5-10 mm margin. Interpectoral lymph nodes, the supraclavicular fossa, and lymph nodes in level 2 and 3 of the axilla are included.

The anatomical landmarks for PTV of the supra- / infraclavicular field are:

- medial border: 5 mm lateral of trachea.
- lateral border: the lateral part of the pectoralis minor muscle.
- cranial border medially: 2 cm cranial of the medial part of the clavicle.
- cranial border laterally: level with the highest part of the acromioclavicular joint (the joint not included).
- caudal border: level with the caudal insertion of the pectoralis minor muscle.
- ventral border: 5 mm below the skin surface.
- dorsal border: 1/2 anterior-posterior distance at the level of the top of the axilla.
