# Supplementary material for: Automated segmentation of target volumes in breast cancer radiotherapy, impact on target size and dose to organs at risk
Source: Clin Transl Radiat Oncol. 2025 May 28;53:100986. doi: 10.1016/j.ctro.2025.100986 (PMC12173629; doi:10.1016/j.ctro.2025.100986)
Supplement: Supplementary Data 2 [file mmc2.pdf]

Slice at 16.50 cm (# 5/70)

**Patient name**	med körtelengagemang, bröst ca. I och II
**Patient id**	200609051245
**Case**	PTV mallar
**Plan**	Mallar
**Treatment position**	HFS
**Last saved**	29 Oct 2014 17:44:32

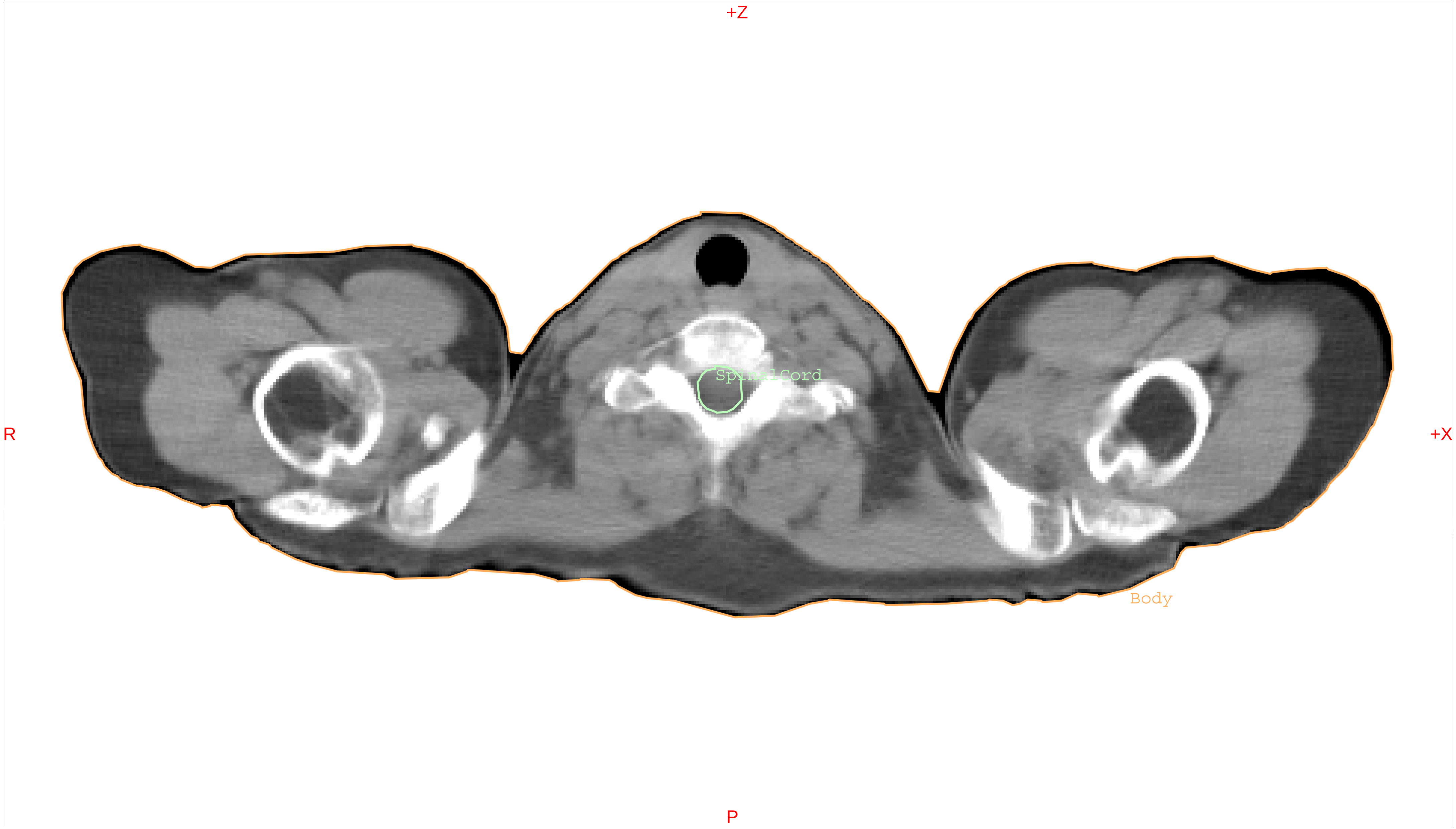

Scale 1:1.6 0 5 10 15 20 25 30 35 40 cm

Slice at 16.00 cm (# 6/70)

**Patient name**	med körtelengagemang, bröst ca. I och II
**Patient id**	200609051245
**Case**	PTV mallar
**Plan**	Mallar
**Treatment position**	HFS
**Last saved**	29 Oct 2014 17:44:32

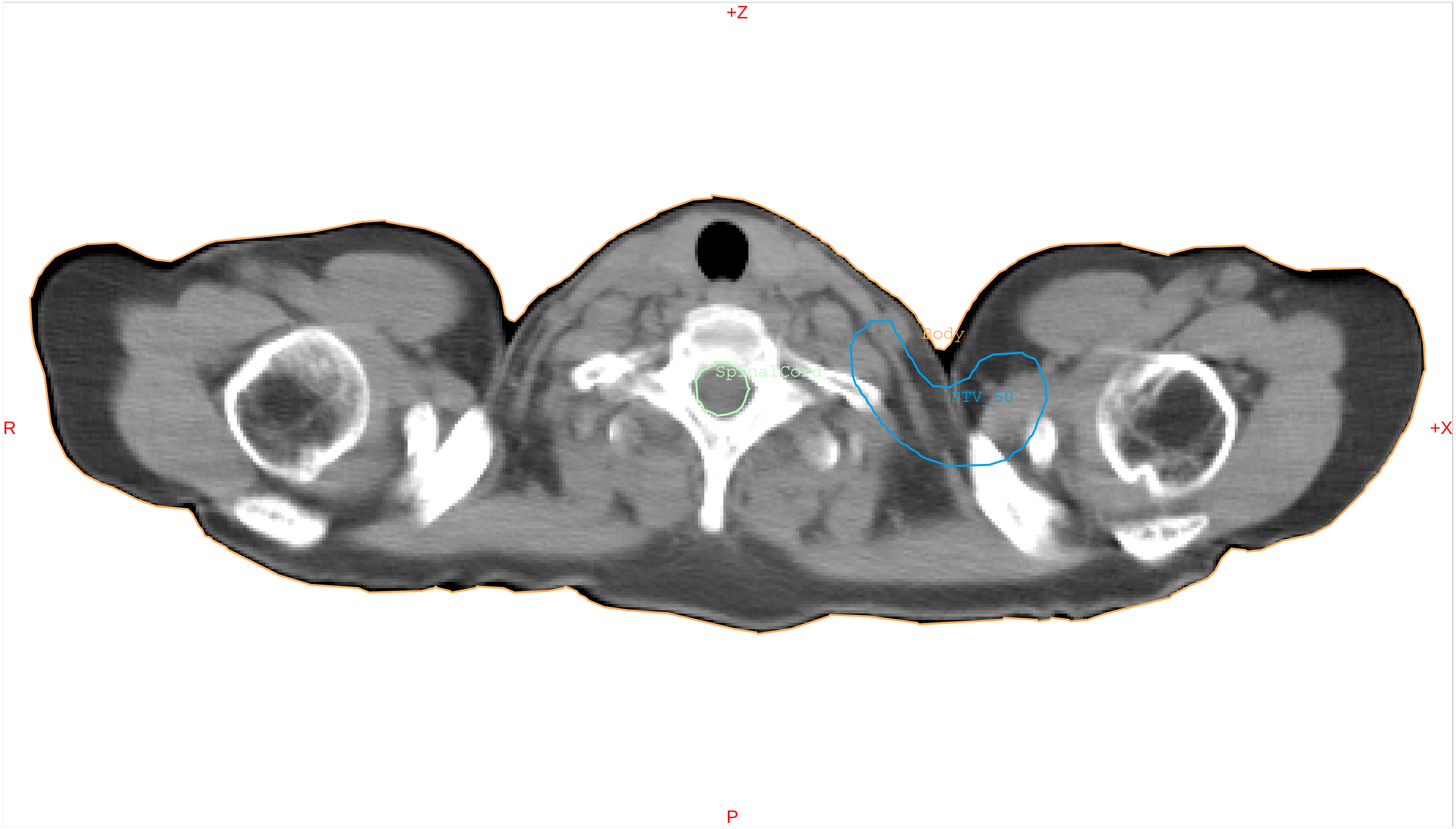

Scale 1:1.5 0 5 10 15 20 25 30 35 cm

Slice at 15.50 cm (# 7/70)

**Patient name**	med körtelengagemang, bröst ca. I och II
**Patient id**	200609051245
**Case**	PTV mallar
**Plan**	Mallar
**Treatment position**	HFS
**Last saved**	29 Oct 2014 17:44:32

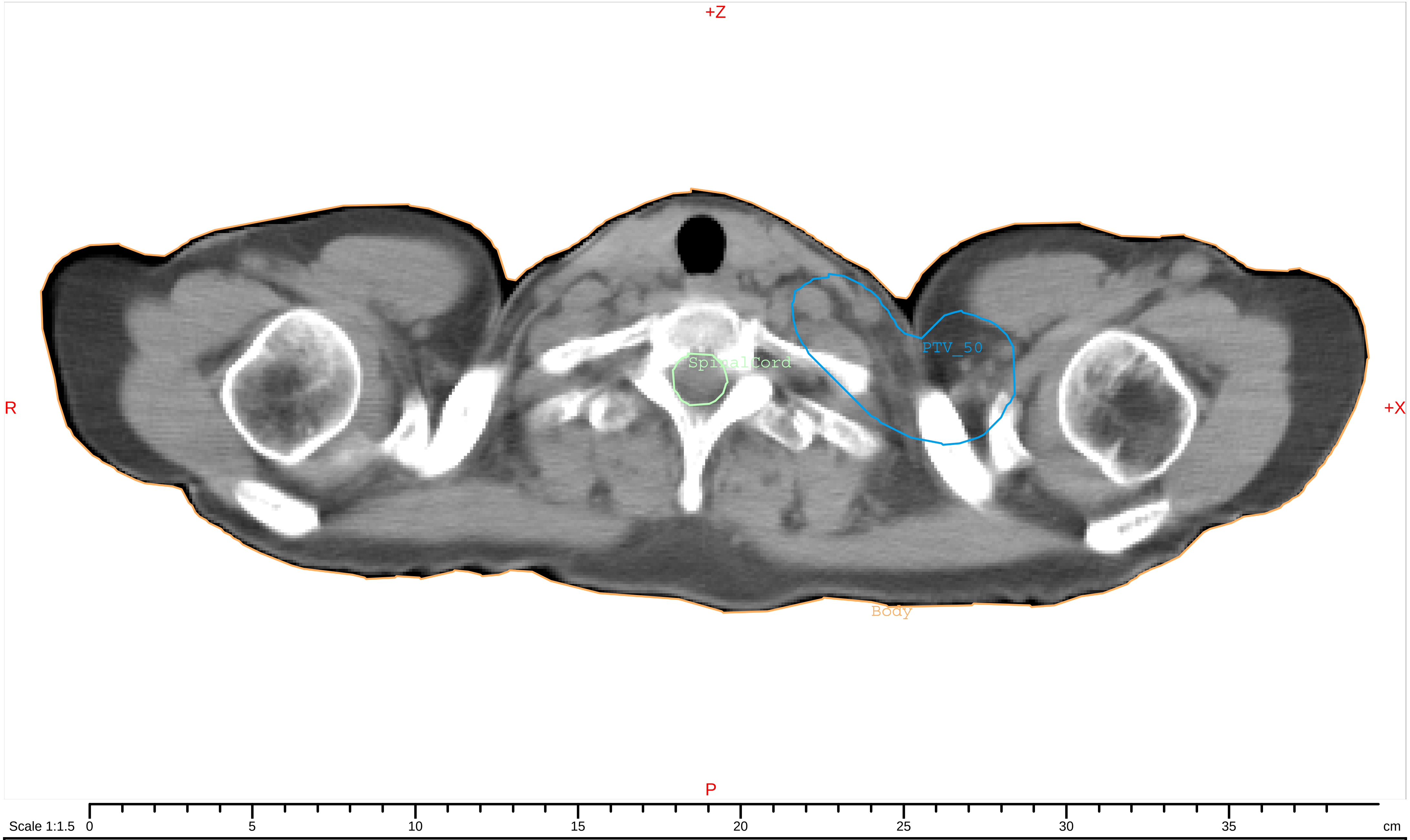

Slice at 15.00 cm (# 8/70)

**Patient name**	med körtelengagemang, bröst ca. I och II
**Patient id**	200609051245
**Case**	PTV mallar
**Plan**	Mallar
**Treatment position**	HFS
**Last saved**	29 Oct 2014 17:44:32

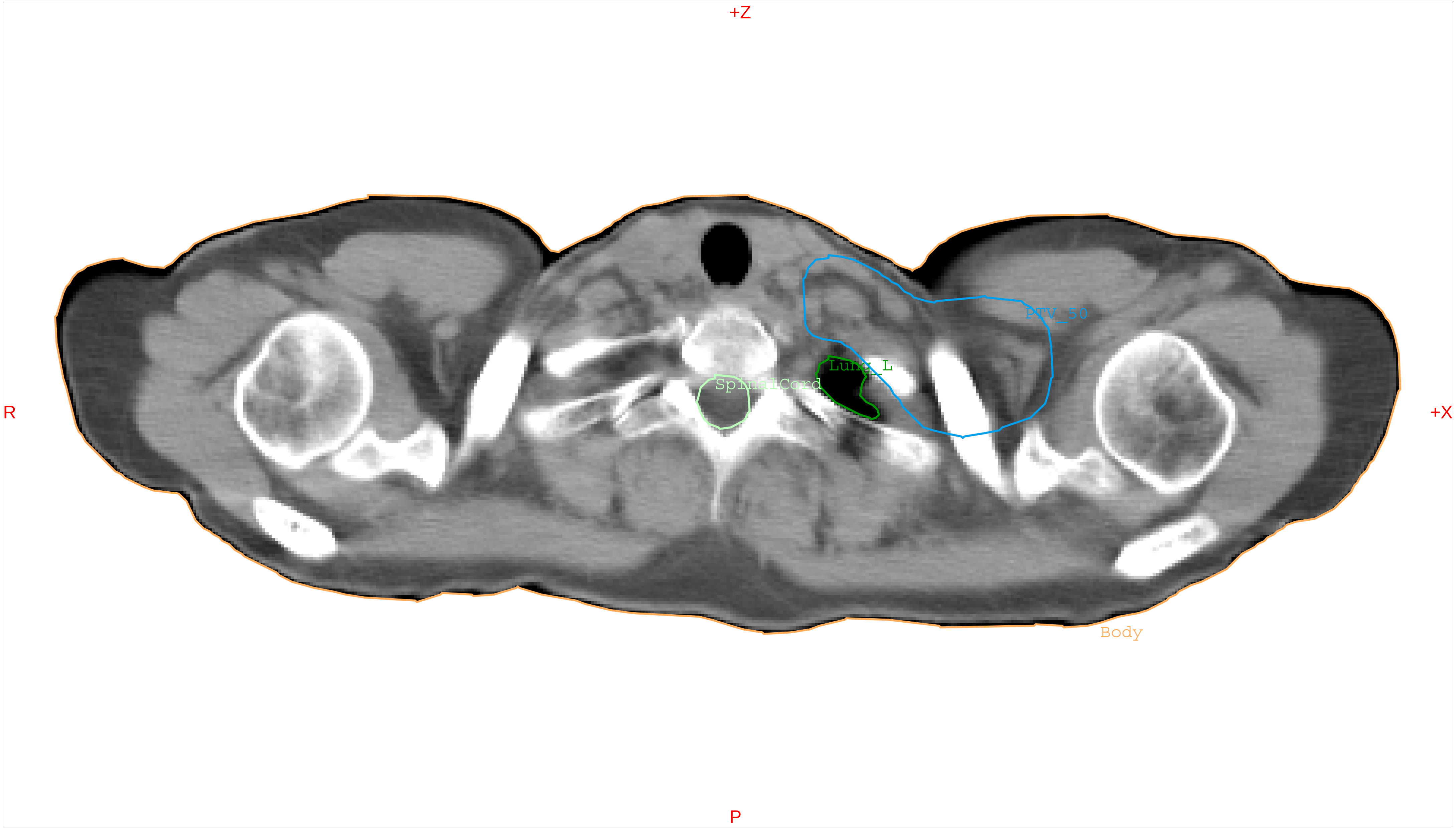

Scale 1:1.5 0 5 10 15 20 25 30 35 cm

Slice at 14.50 cm (# 9/70)

**Patient name**	med körtelengagemang, bröst ca. I och II
**Patient id**	200609051245
**Case**	PTV mallar
**Plan**	Mallar
**Treatment position**	HFS
**Last saved**	29 Oct 2014 17:44:32

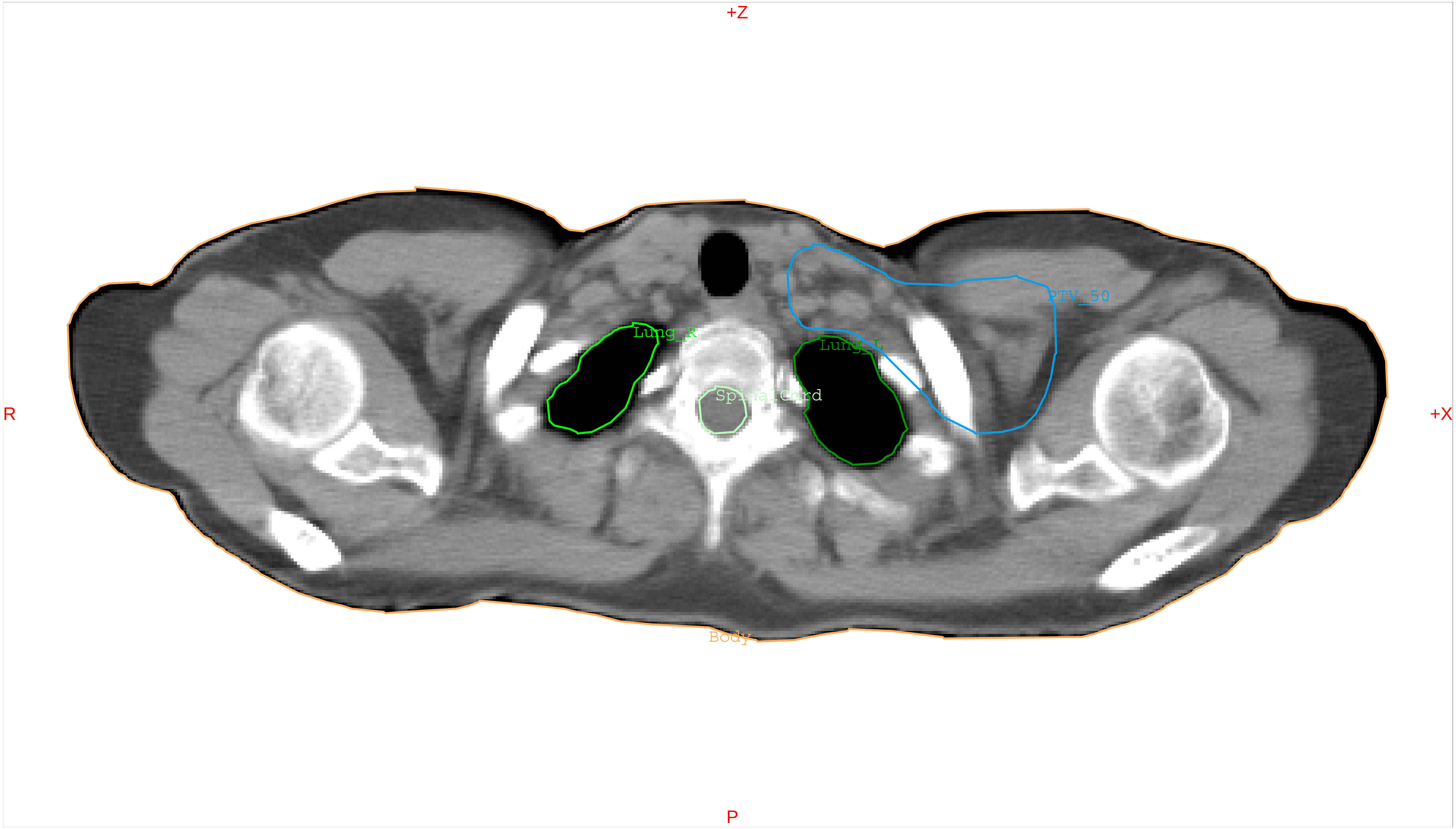

Scale 1:1.5 0 5 10 15 20 25 30 35 cm

Slice at 14.00 cm (# 10/70)

**Patient name**	med körtelengagemang, bröst ca. I och II
**Patient id**	200609051245
**Case**	PTV mallar
**Plan**	Mallar
**Treatment position**	HFS
**Last saved**	29 Oct 2014 17:44:32

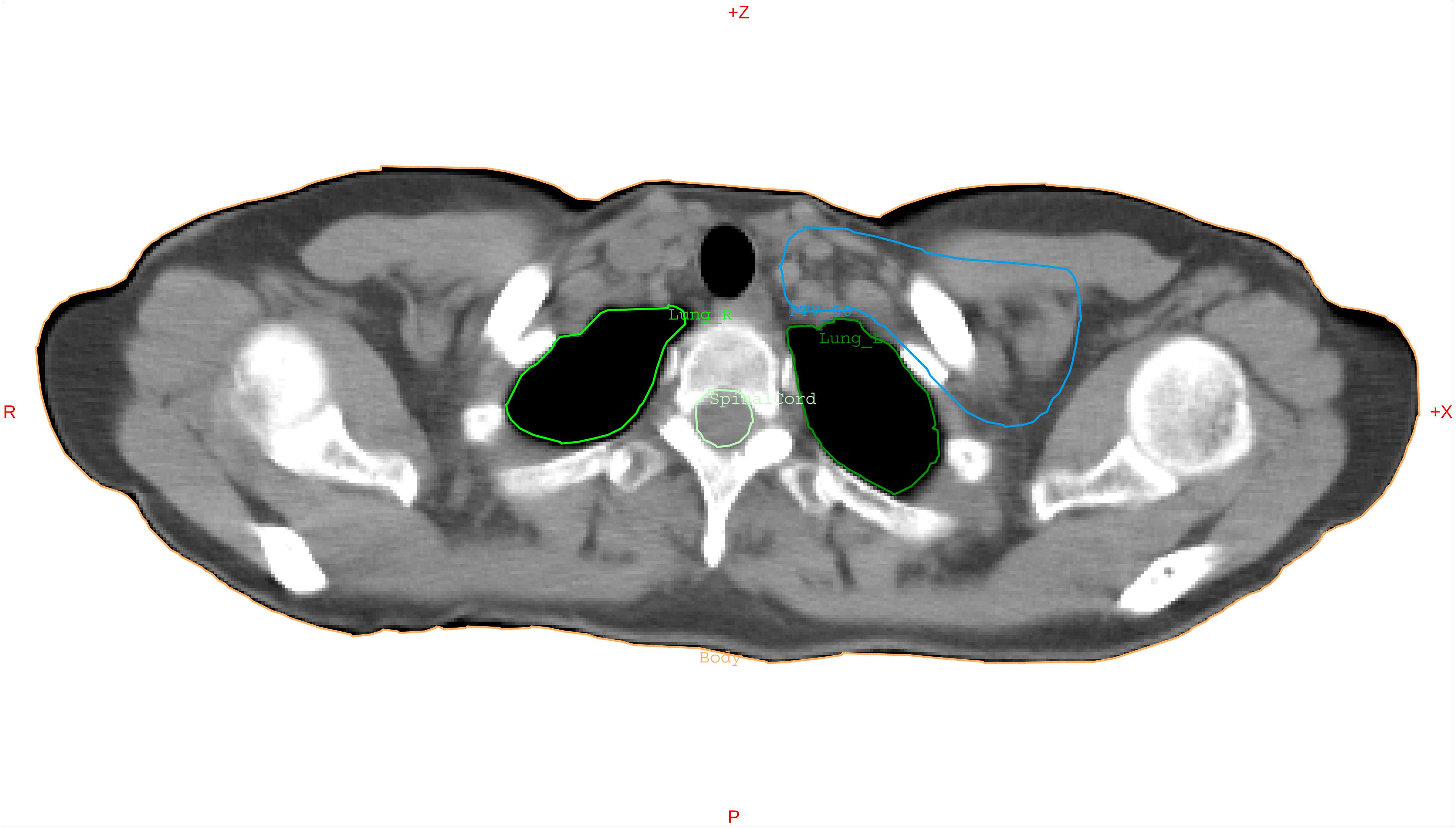

Scale 1:1.4 0 5 10 15 20 25 30 35 cm

Slice at 13.50 cm (# 11/70)

**Patient name**	med körtelengagemang, bröst ca. I och II
**Patient id**	200609051245
**Case**	PTV mallar
**Plan**	Mallar
**Treatment position**	HFS
**Last saved**	29 Oct 2014 17:44:32

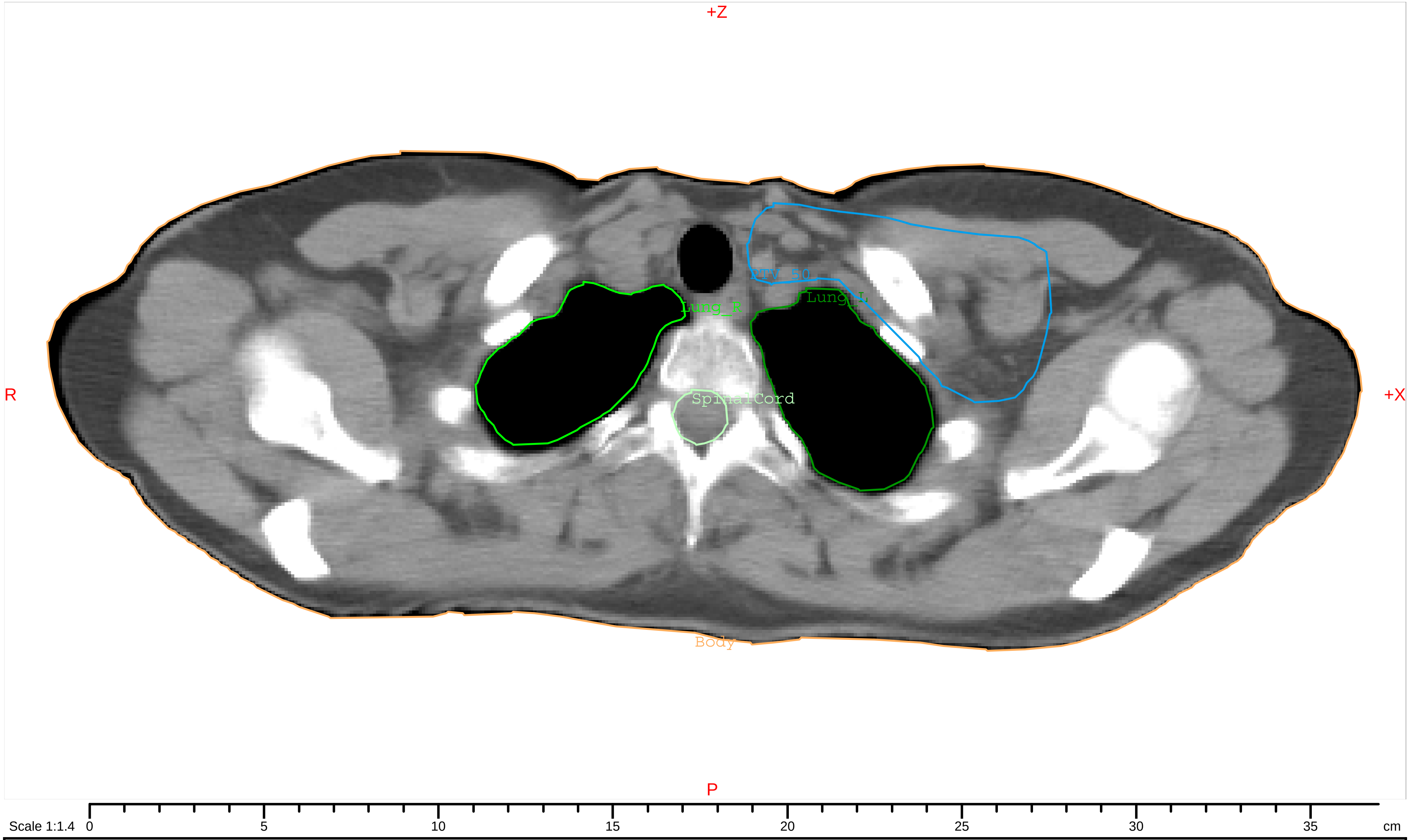

Slice at 13.00 cm (# 12/70)

**Patient name**	med körtelengagemang, bröst ca. I och II
**Patient id**	200609051245
**Case**	PTV mallar
**Plan**	Mallar
**Treatment position**	HFS
**Last saved**	29 Oct 2014 17:44:32

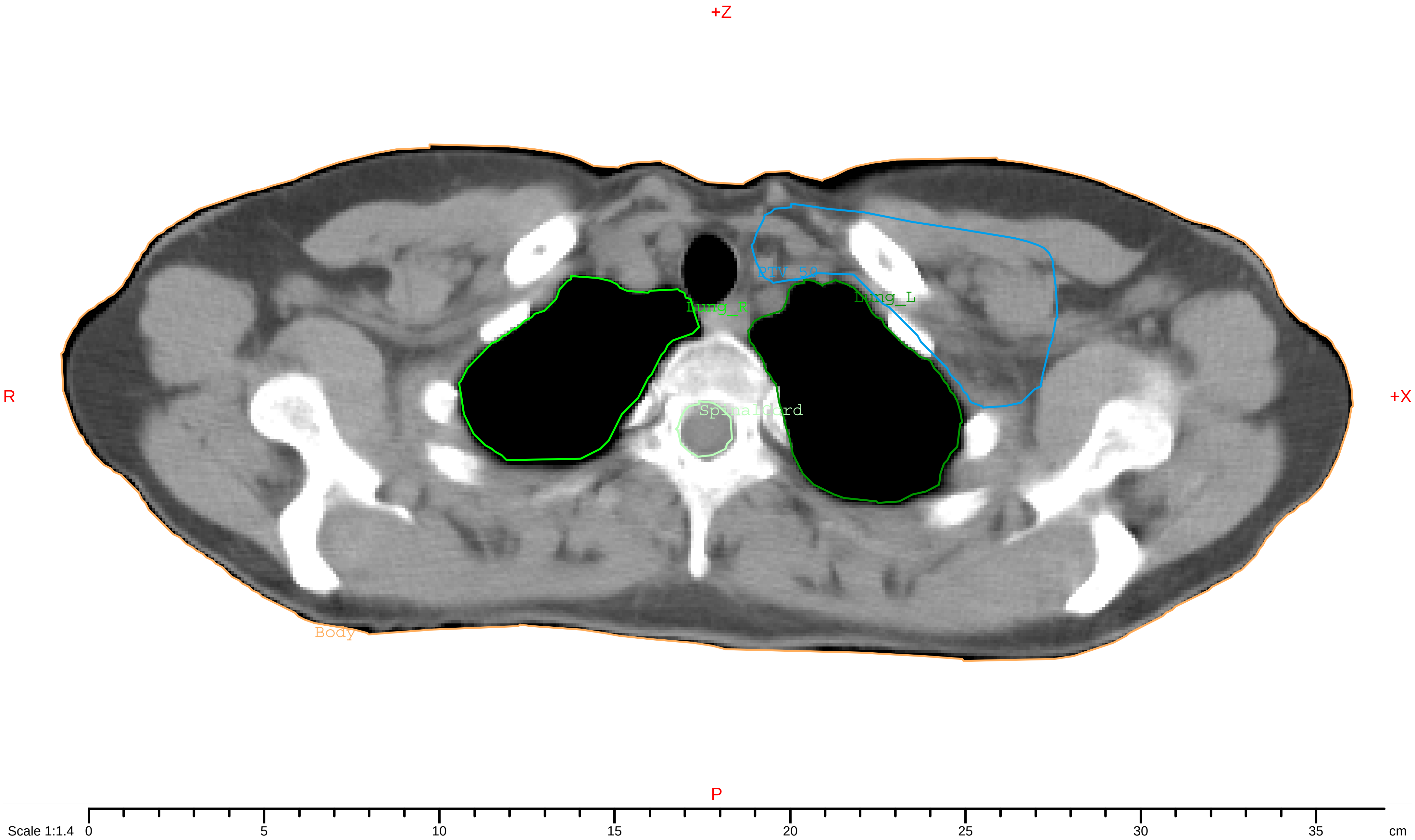

Slice at 12.50 cm (# 13/70)

**Patient name**	med körtelengagemang, bröst ca. I och II
**Patient id**	200609051245
**Case**	PTV mallar
**Plan**	Mallar
**Treatment position**	HFS
**Last saved**	29 Oct 2014 17:44:32

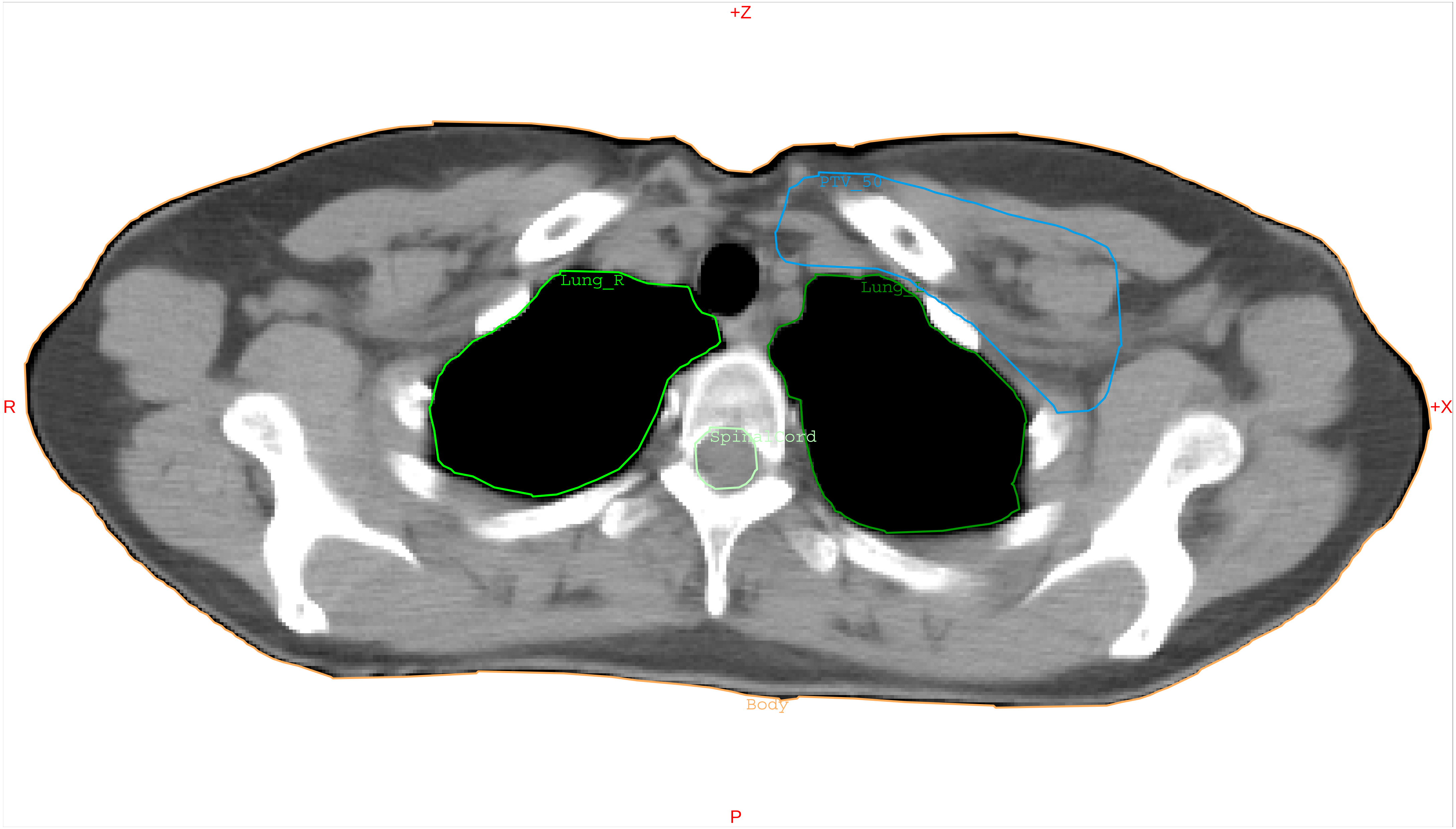

Scale 1:1.3 0 5 10 15 20 25 30 cm

Slice at 12.00 cm (# 14/70)

**Patient name**	med körtelengagemang, bröst ca. I och II
**Patient id**	200609051245
**Case**	PTV mallar
**Plan**	Mallar
**Treatment position**	HFS
**Last saved**	29 Oct 2014 17:44:32

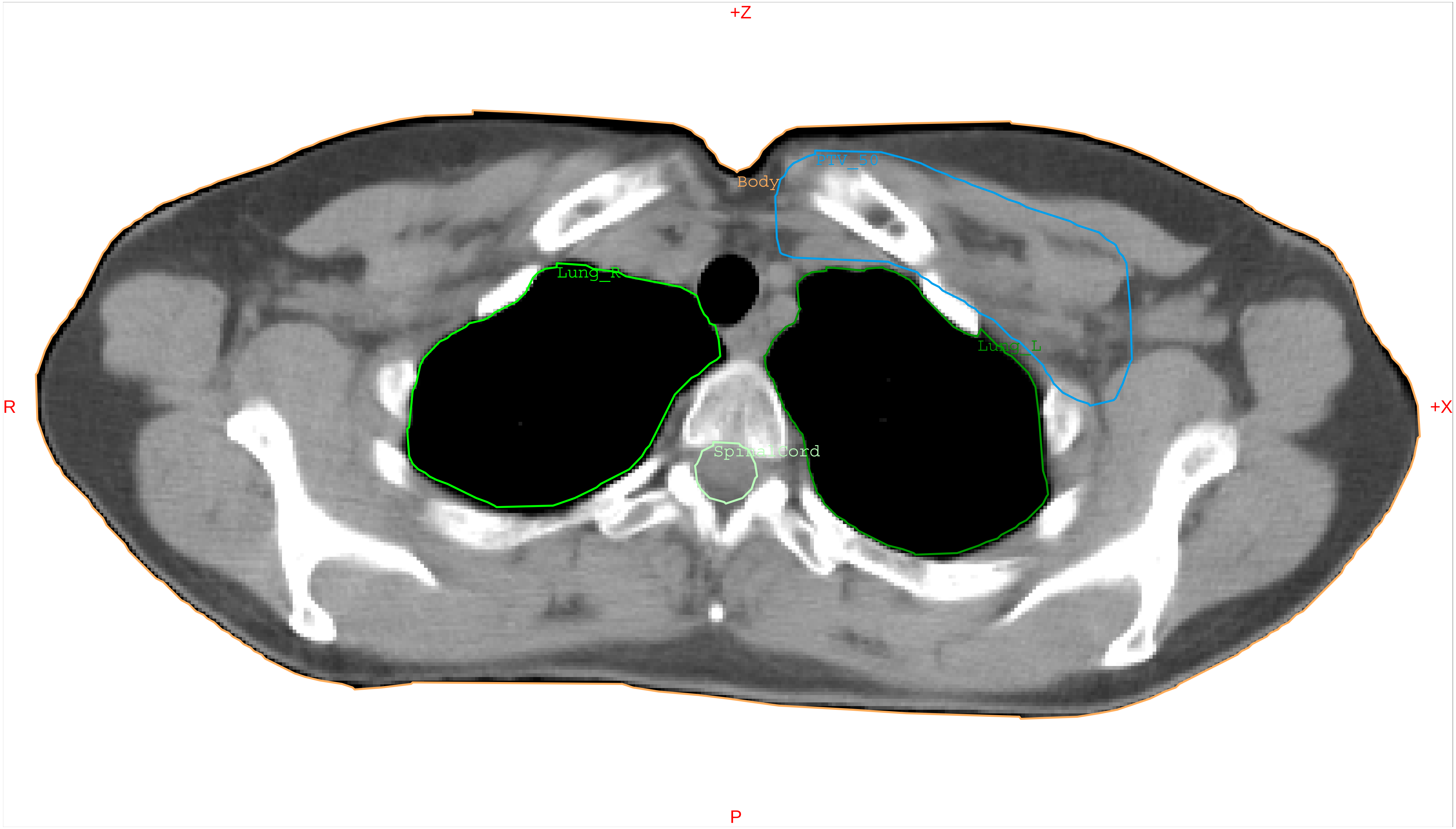

Scale 1:1.3 0 5 10 15 20 25 30 cm

Slice at 11.50 cm (# 15/70)

**Patient name**	med körtelengagemang, bröst ca. I och II
**Patient id**	200609051245
**Case**	PTV mallar
**Plan**	Mallar
**Treatment position**	HFS
**Last saved**	29 Oct 2014 17:44:32

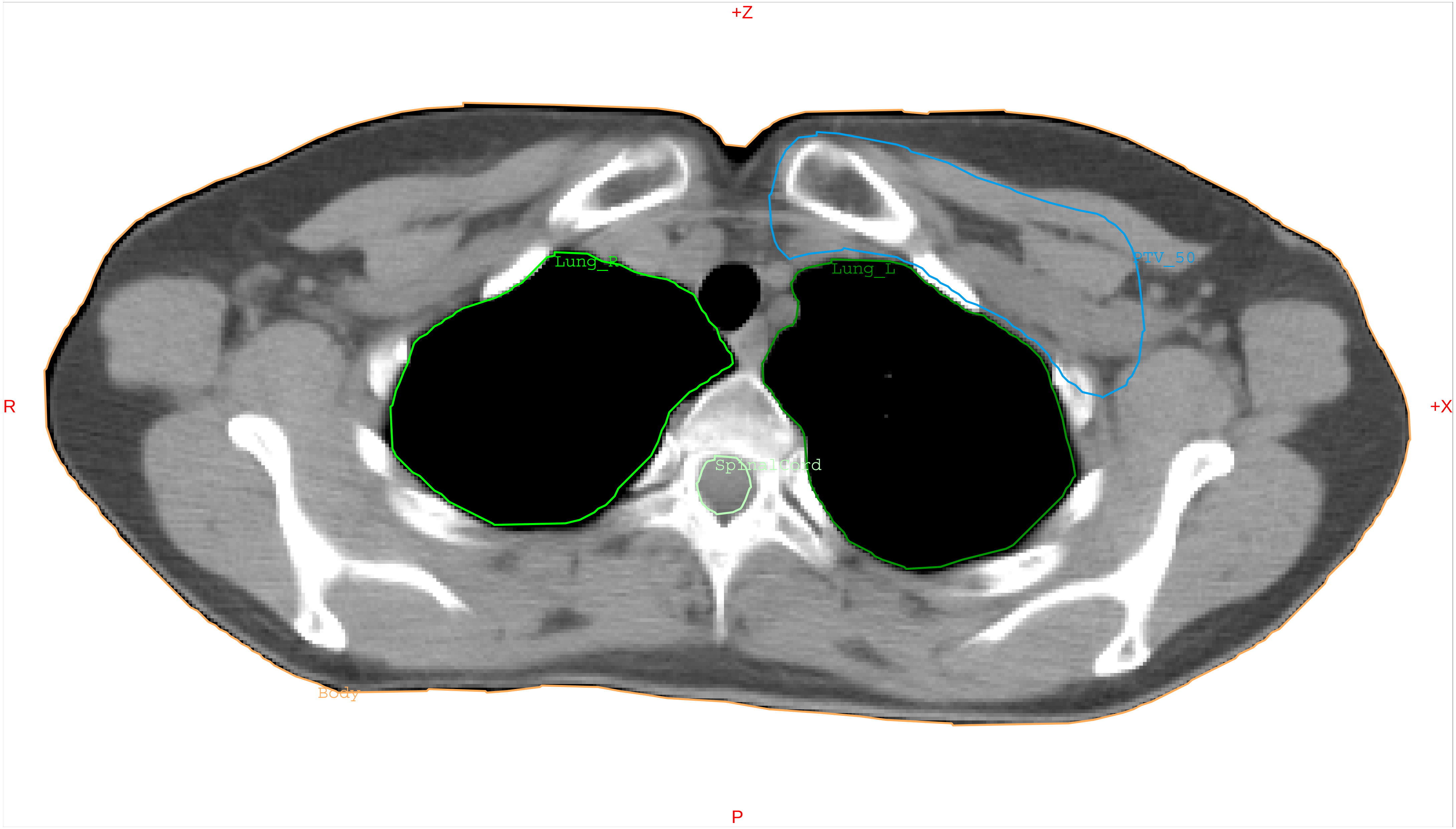

Scale 1:1.3 0 5 10 15 20 25 30 cm

Slice at 11.00 cm (# 16/70)

**Patient name**	med körtelengagemang, bröst ca. I och II
**Patient id**	200609051245
**Case**	PTV mallar
**Plan**	Mallar
**Treatment position**	HFS
**Last saved**	29 Oct 2014 17:44:32

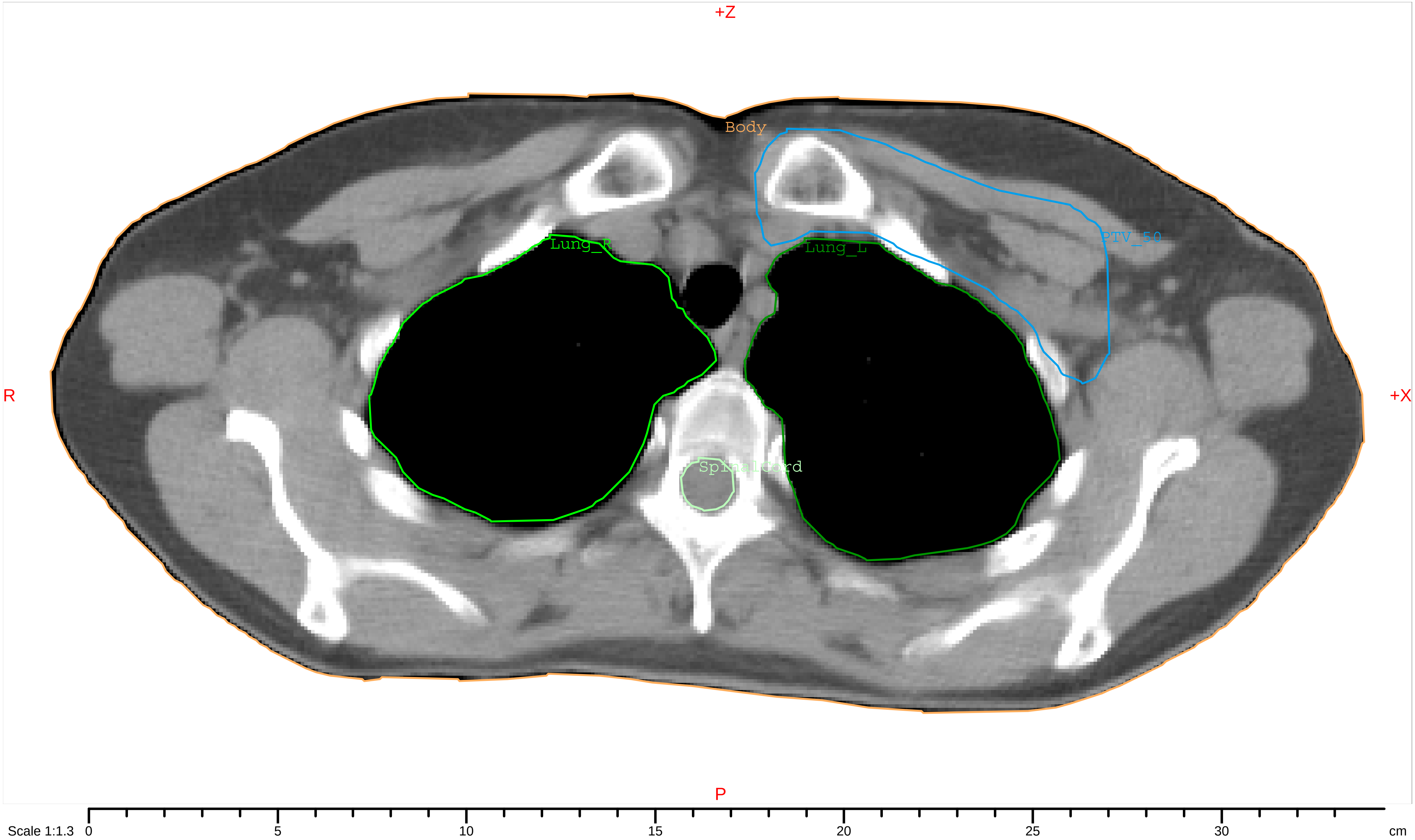

Slice at 10.50 cm (# 17/70)

**Patient name**	med körtelengagemang, bröst ca. I och II
**Patient id**	200609051245
**Case**	PTV mallar
**Plan**	Mallar
**Treatment position**	HFS
**Last saved**	29 Oct 2014 17:44:32

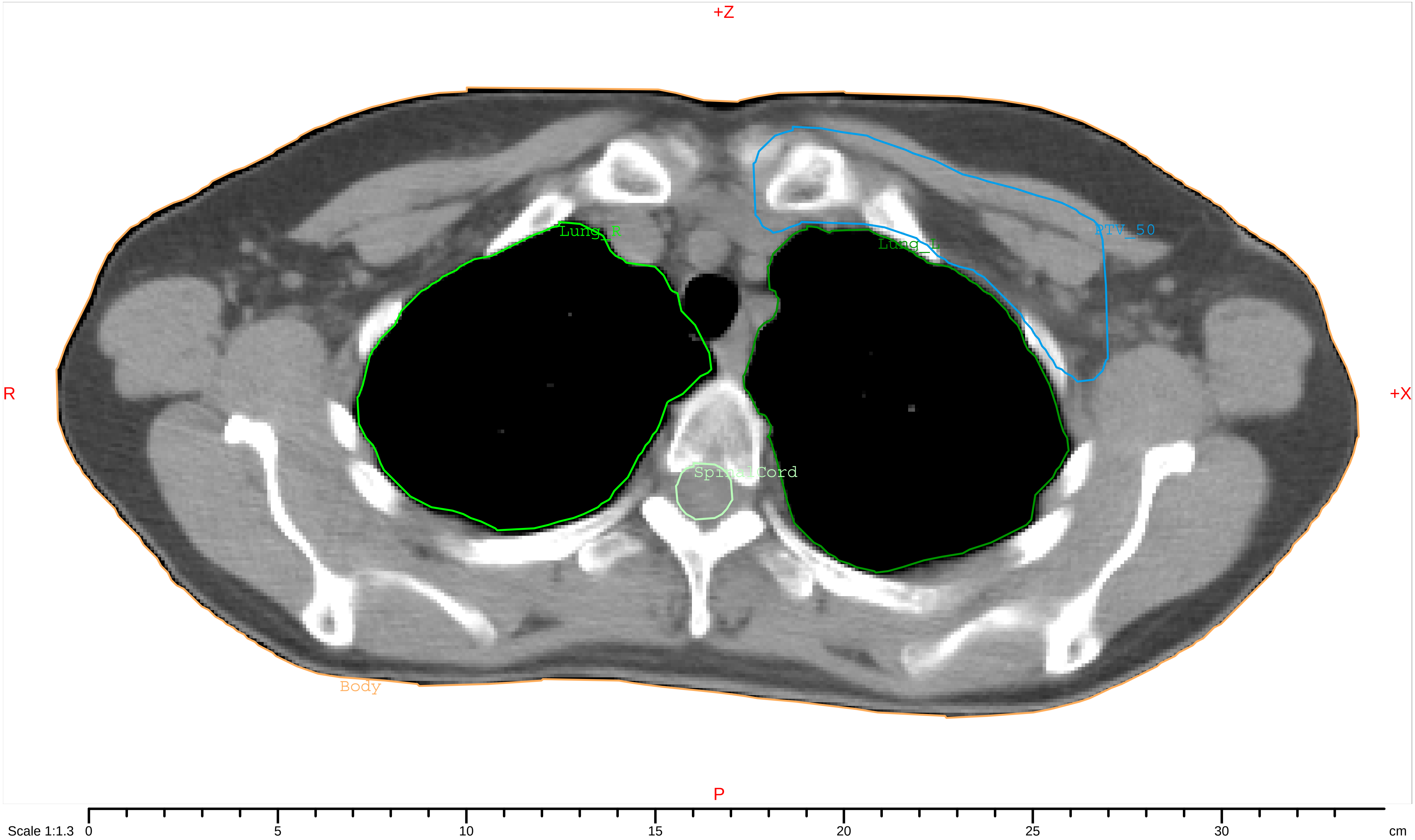

Slice at 10.00 cm (# 18/70)

**Patient name**	med körtelengagemang, bröst ca. I och II
**Patient id**	200609051245
**Case**	PTV mallar
**Plan**	Mallar
**Treatment position**	HFS
**Last saved**	29 Oct 2014 17:44:32

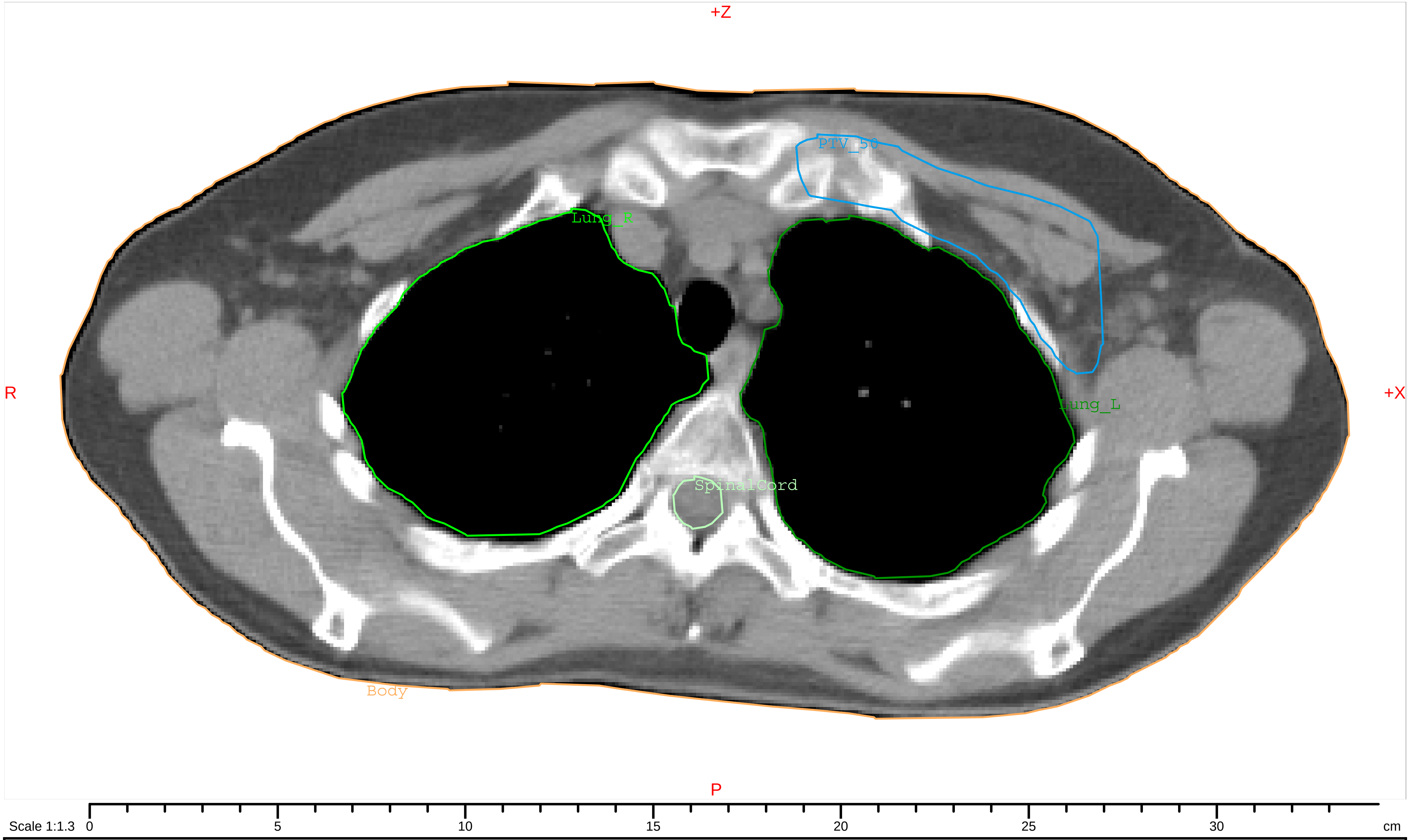

Slice at 9.50 cm (# 19/70)

**Patient name**	med körtelengagemang, bröst ca. I och II
**Patient id**	200609051245
**Case**	PTV mallar
**Plan**	Mallar
**Treatment position**	HFS
**Last saved**	29 Oct 2014 17:44:32

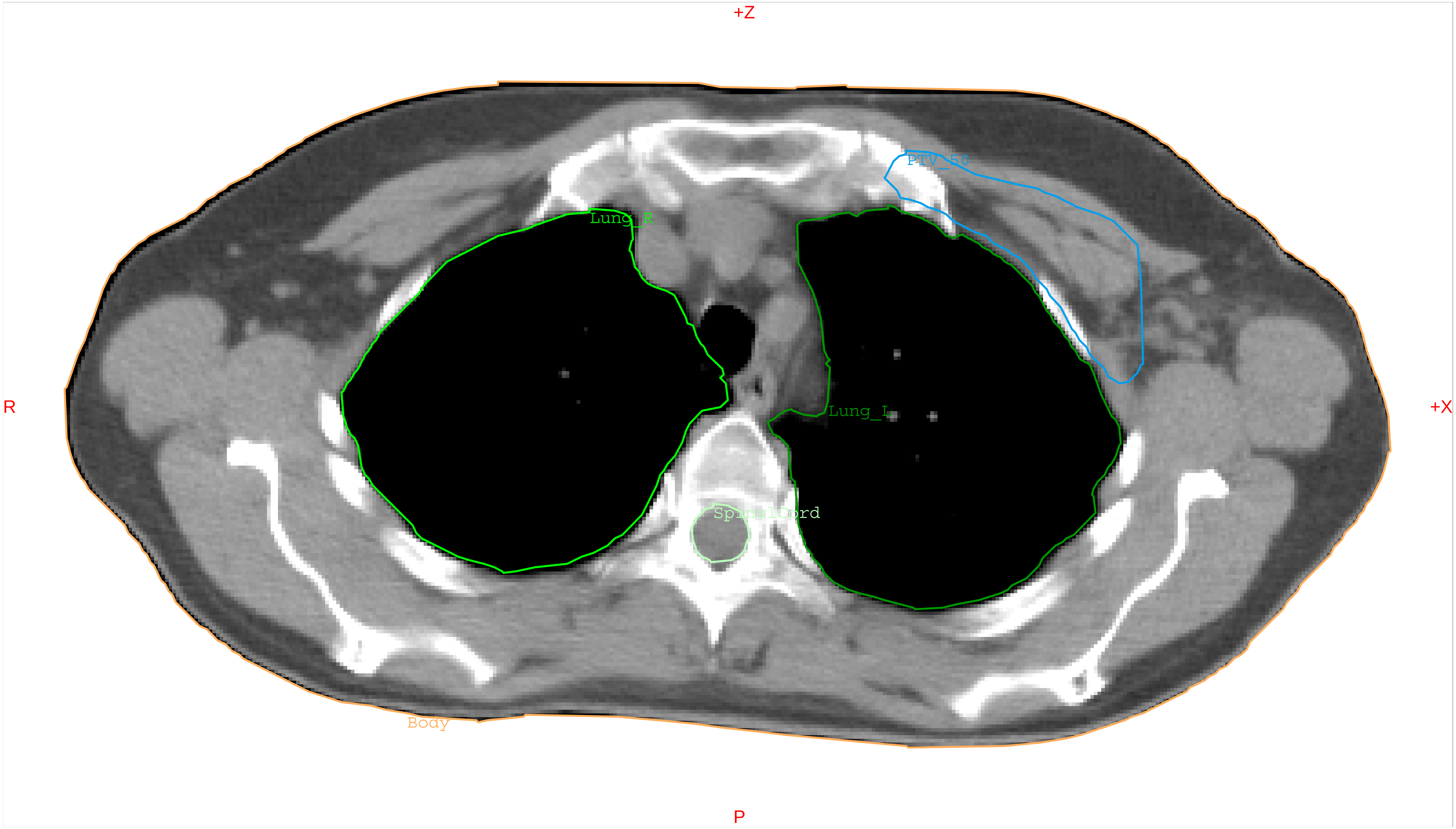

Scale 1:1.3 0 5 10 15 20 25 30 cm

Slice at 9.00 cm (# 20/70)

**Patient name**	med körtelengagemang, bröst ca. I och II
**Patient id**	200609051245
**Case**	PTV mallar
**Plan**	Mallar
**Treatment position**	HFS
**Last saved**	29 Oct 2014 17:44:32

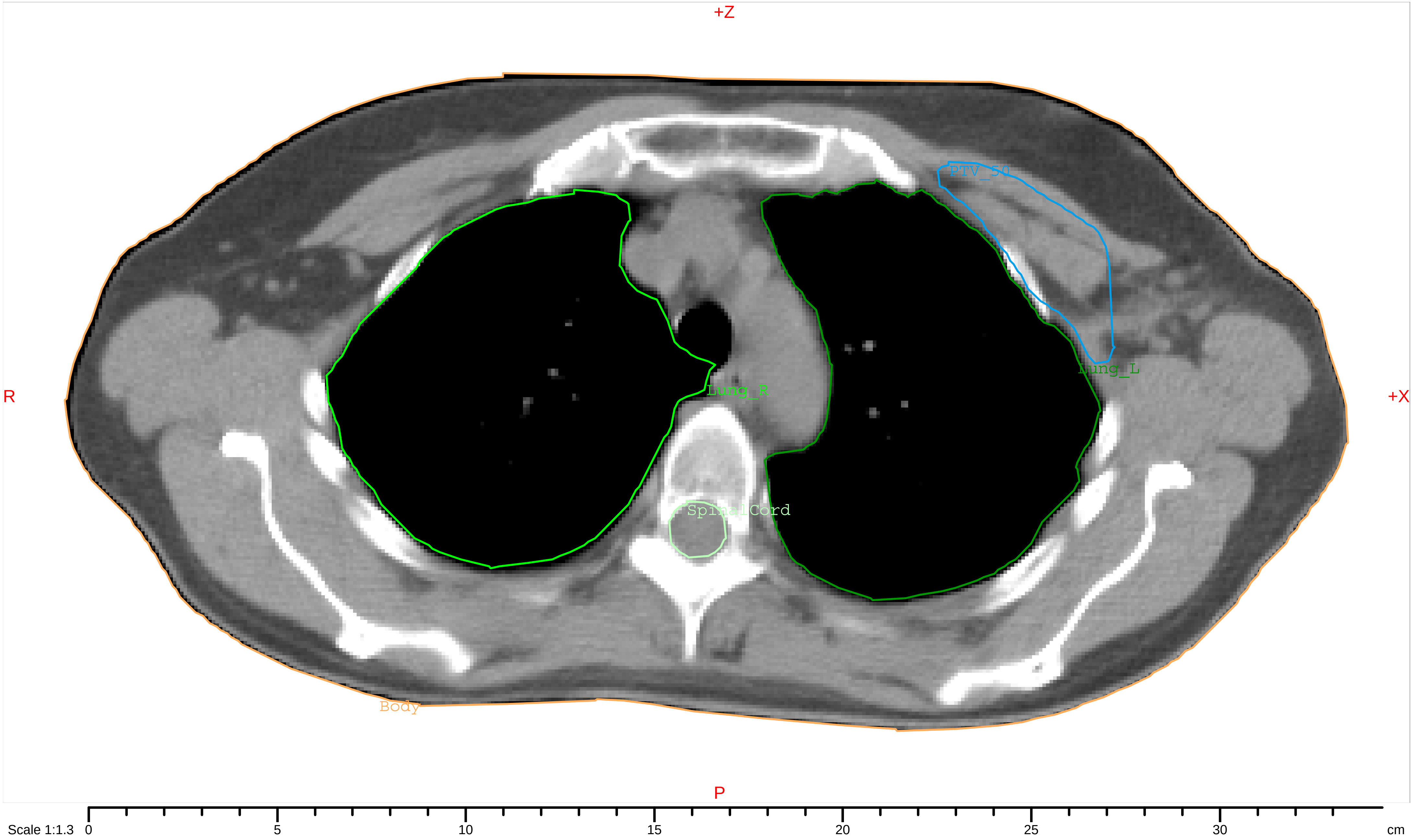

Slice at 8.50 cm (# 21/70)

**Patient name**	med körtelengagemang, bröst ca. I och II
**Patient id**	200609051245
**Case**	PTV mallar
**Plan**	Mallar
**Treatment position**	HFS
**Last saved**	29 Oct 2014 17:44:32

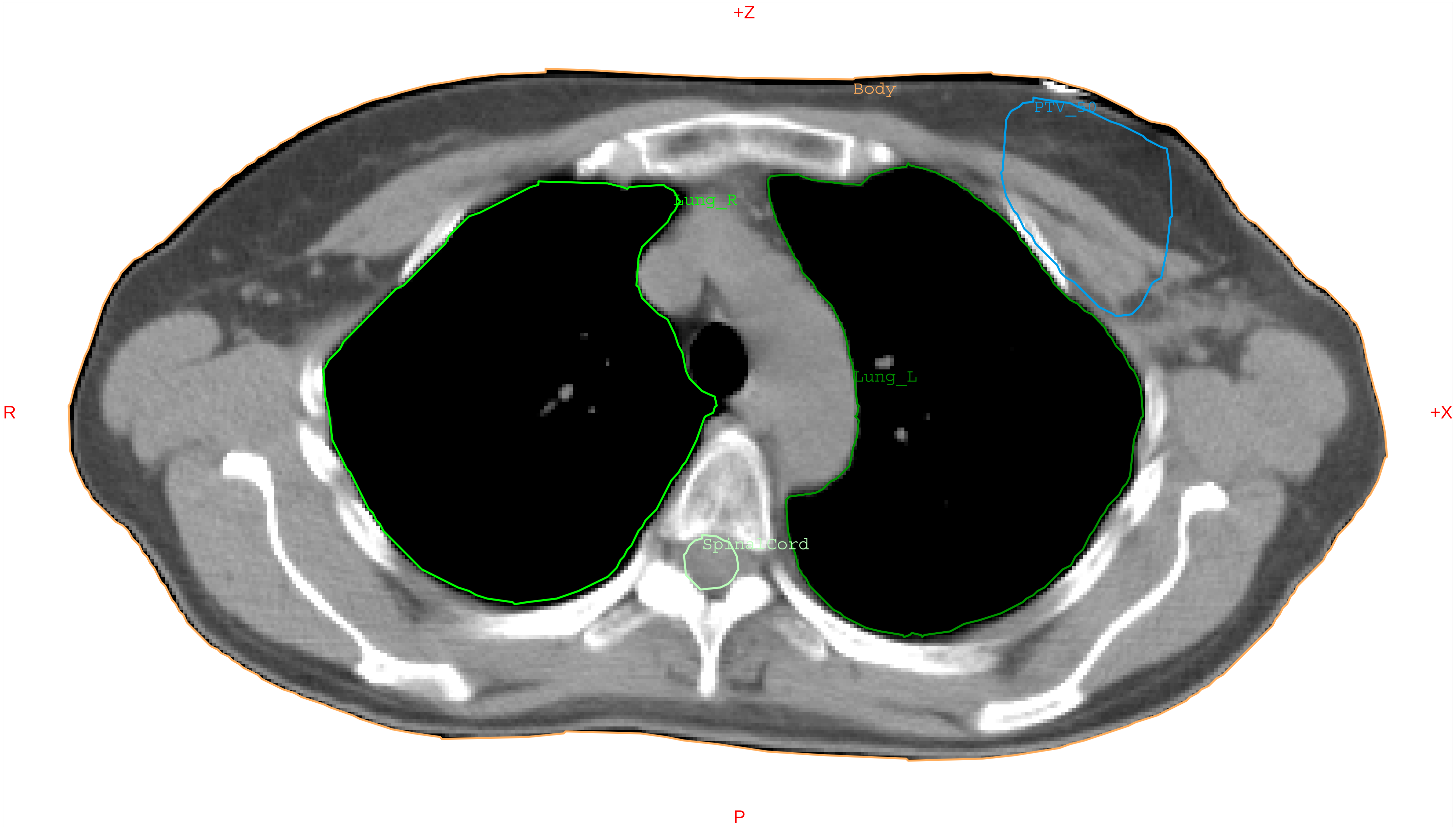

Scale 1:1.3 0 5 10 15 20 25 30 cm

Slice at 8.00 cm (# 22/70)

**Patient name**	med körtelengagemang, bröst ca. I och II
**Patient id**	200609051245
**Case**	PTV mallar
**Plan**	Mallar
**Treatment position**	HFS
**Last saved**	29 Oct 2014 17:44:32

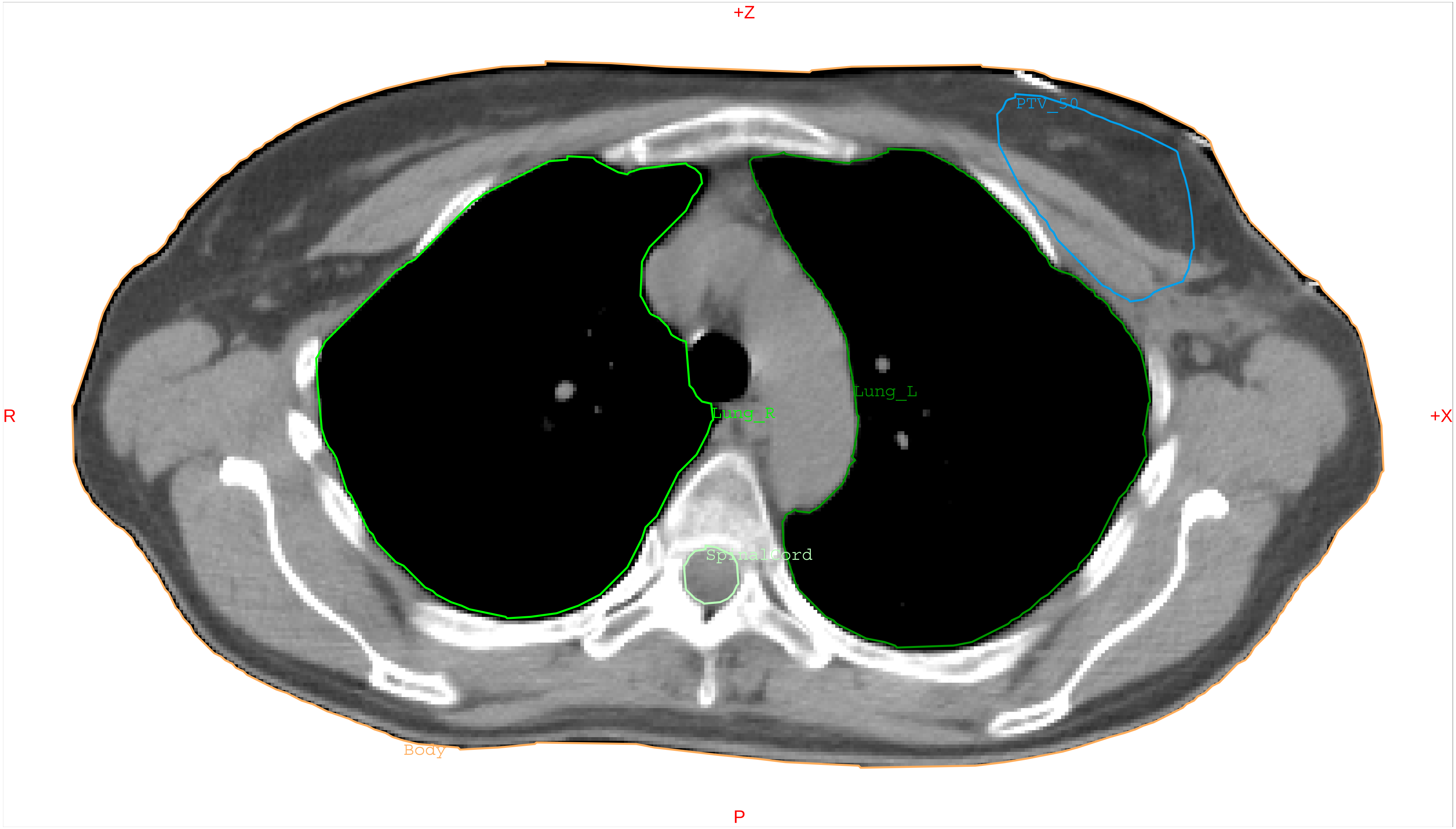

Scale 1:1.3 0 5 10 15 20 25 30 cm

Slice at 7.50 cm (# 23/70)

**Patient name**	med körtelengagemang, bröst ca. I och II
**Patient id**	200609051245
**Case**	PTV mallar
**Plan**	Mallar
**Treatment position**	HFS
**Last saved**	29 Oct 2014 17:44:32

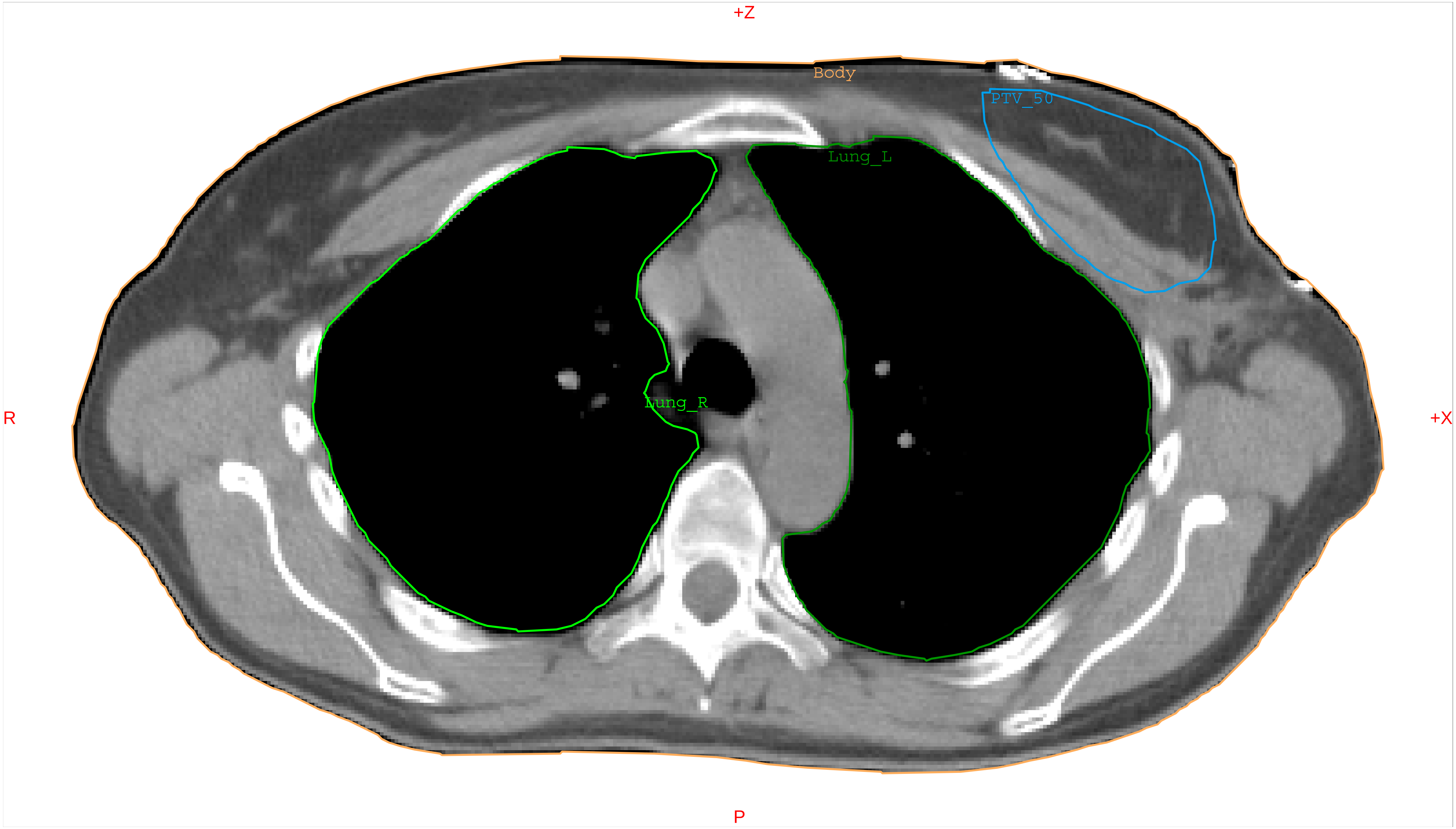

Scale 1:1.3 0 5 10 15 20 25 30 cm

Slice at 7.00 cm (# 24/70)

**Patient name**	med körtelengagemang, bröst ca. I och II
**Patient id**	200609051245
**Case**	PTV mallar
**Plan**	Mallar
**Treatment position**	HFS
**Last saved**	29 Oct 2014 17:44:32

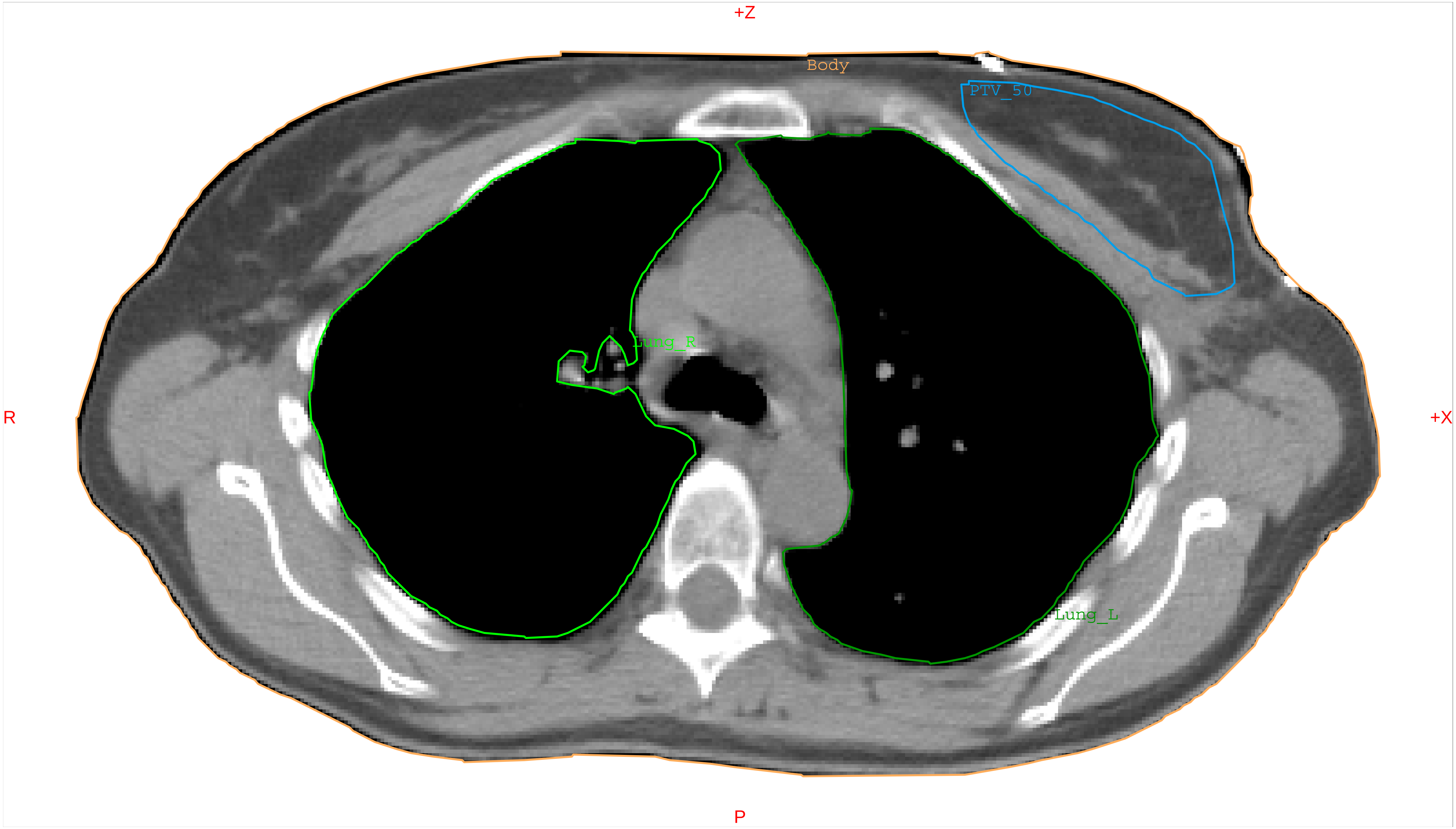

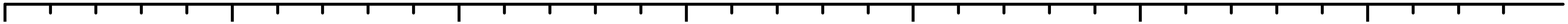

Slice at 6.50 cm (# 25/70)

**Patient name**	med körtelengagemang, bröst ca. I och II
**Patient id**	200609051245
**Case**	PTV mallar
**Plan**	Mallar
**Treatment position**	HFS
**Last saved**	29 Oct 2014 17:44:32

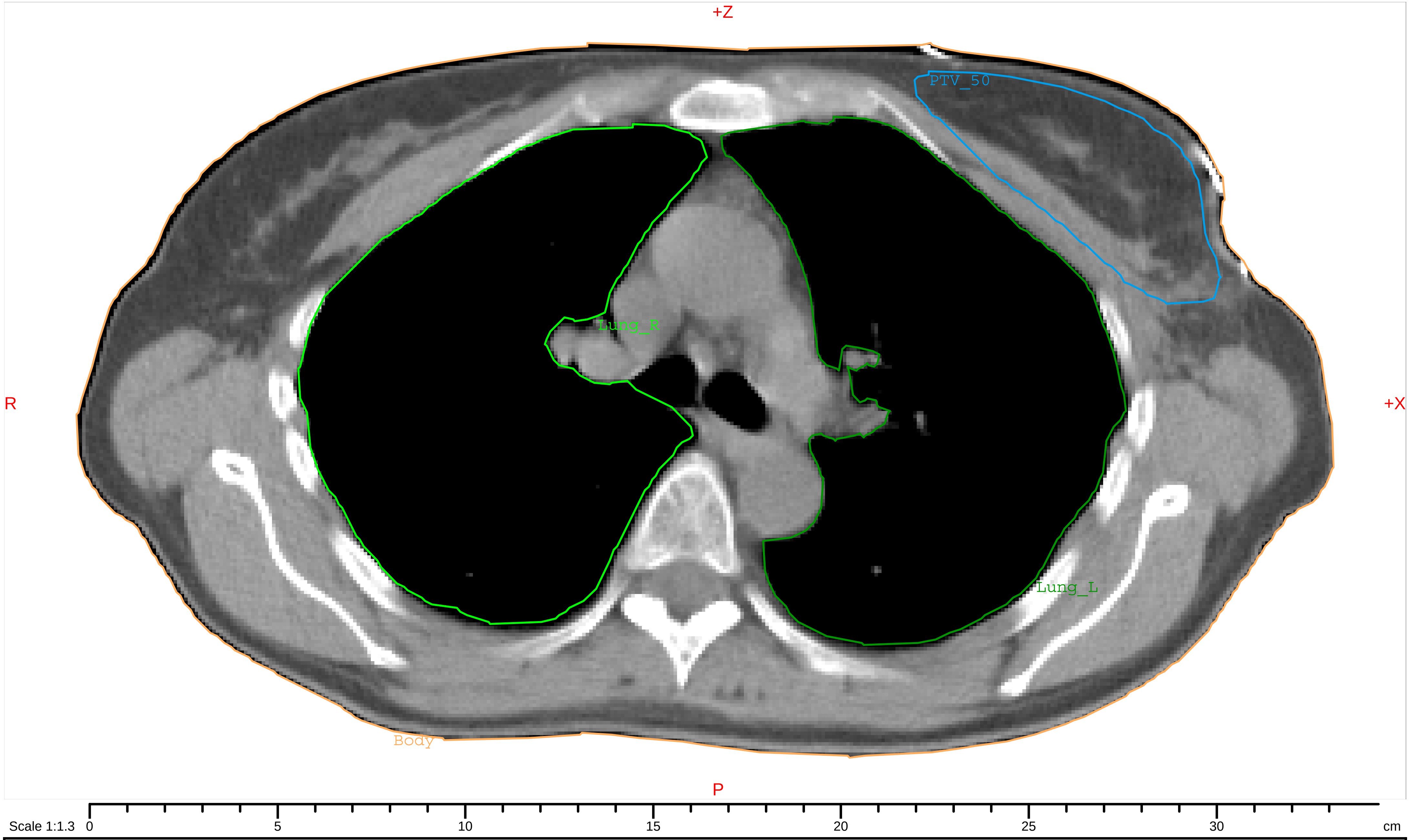

Slice at 6.00 cm (# 26/70)

**Patient name**	med körtelengagemang, bröst ca. I och II
**Patient id**	200609051245
**Case**	PTV mallar
**Plan**	Mallar
**Treatment position**	HFS
**Last saved**	29 Oct 2014 17:44:32

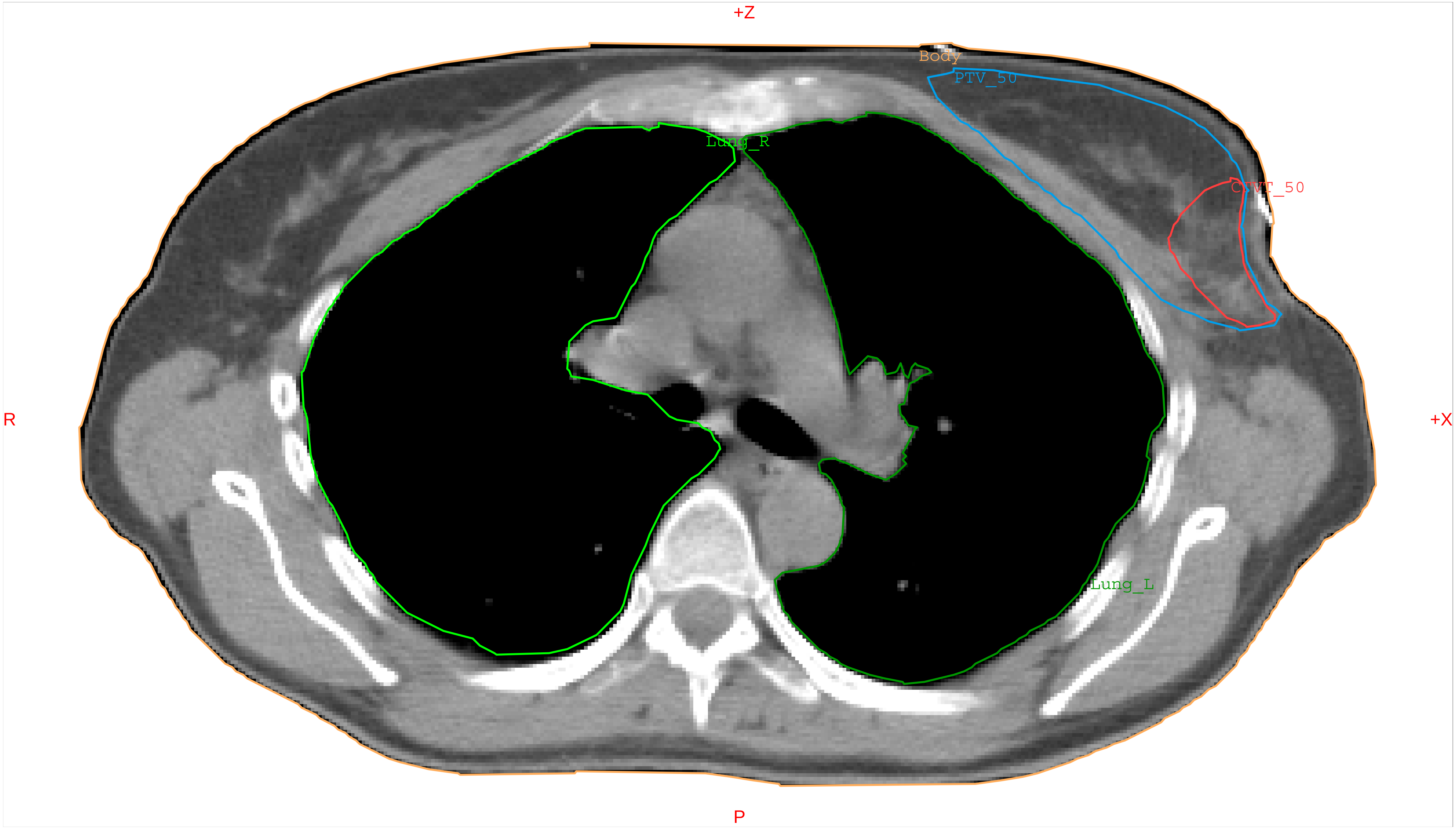

Scale 1:1.3 0 5 10 15 20 25 30 cm

Slice at 5.50 cm (# 27/70)

**Patient name**	med körtelengagemang, bröst ca. I och II
**Patient id**	200609051245
**Case**	PTV mallar
**Plan**	Mallar
**Treatment position**	HFS
**Last saved**	29 Oct 2014 17:44:32

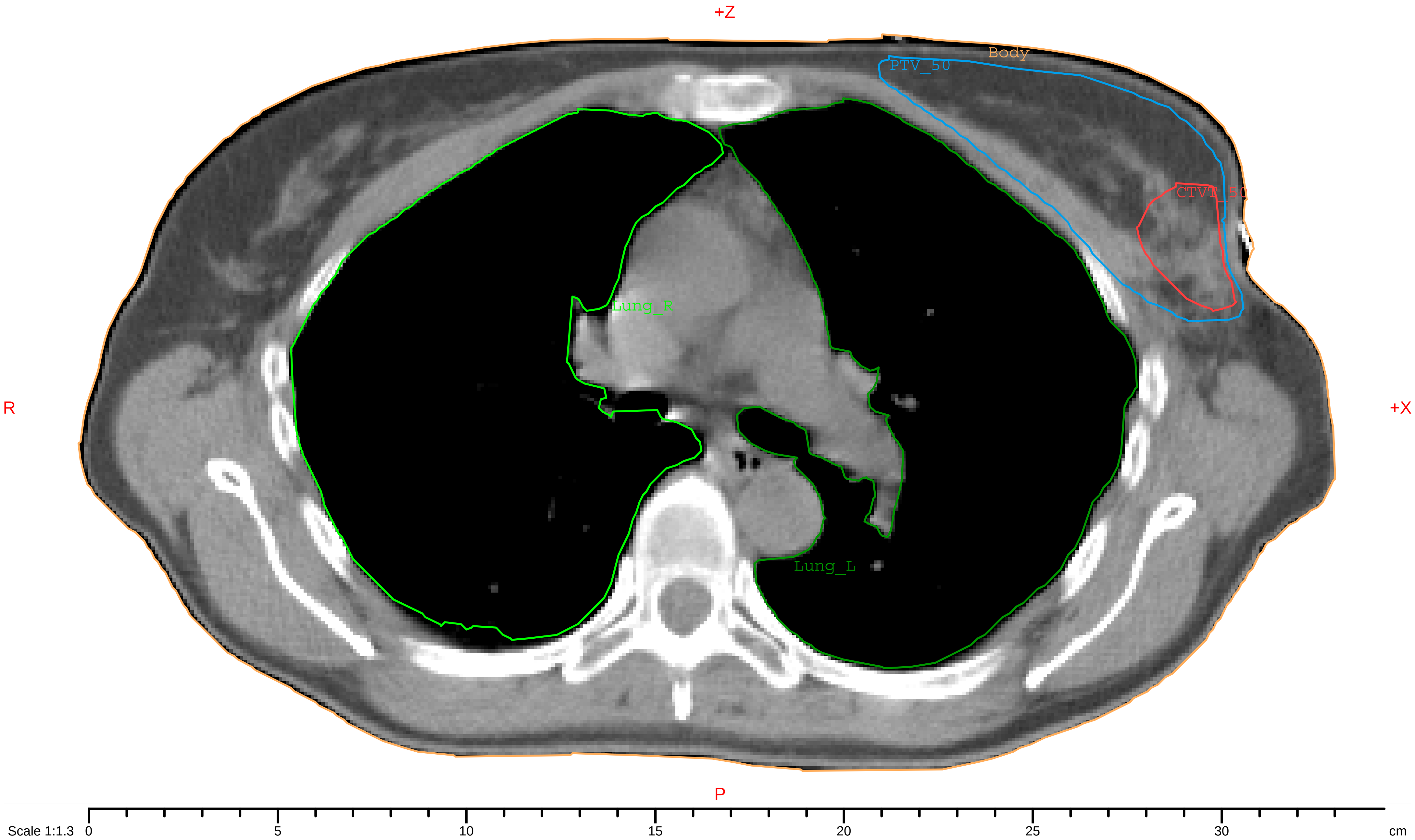

Slice at 5.00 cm (# 28/70)

**Patient name**	med körtelengagemang, bröst ca. I och II
**Patient id**	200609051245
**Case**	PTV mallar
**Plan**	Mallar
**Treatment position**	HFS
**Last saved**	29 Oct 2014 17:44:32

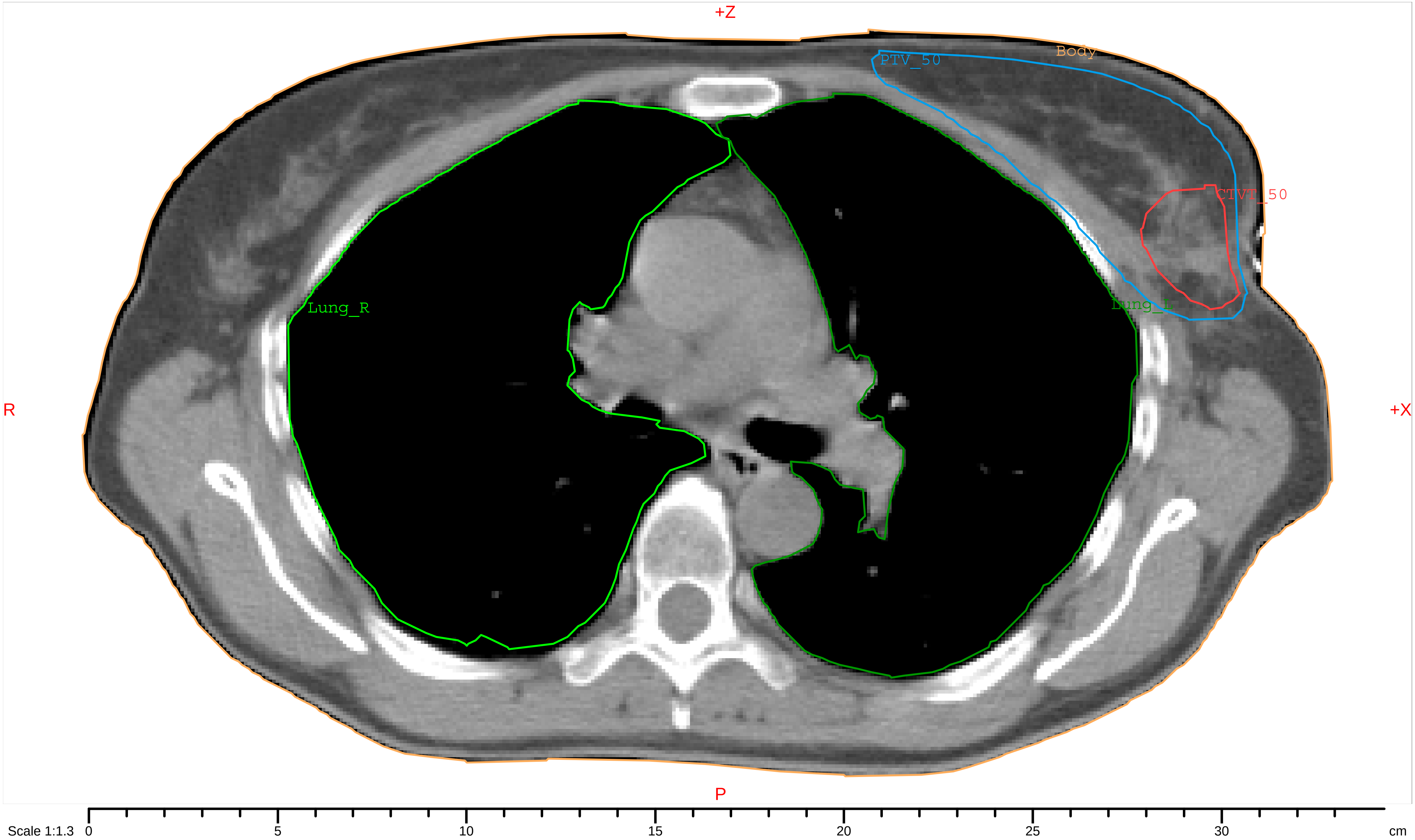

Slice at 4.50 cm (# 29/70)

**Patient name**	med körtelengagemang, bröst ca. I och II
**Patient id**	200609051245
**Case**	PTV mallar
**Plan**	Mallar
**Treatment position**	HFS
**Last saved**	29 Oct 2014 17:44:32

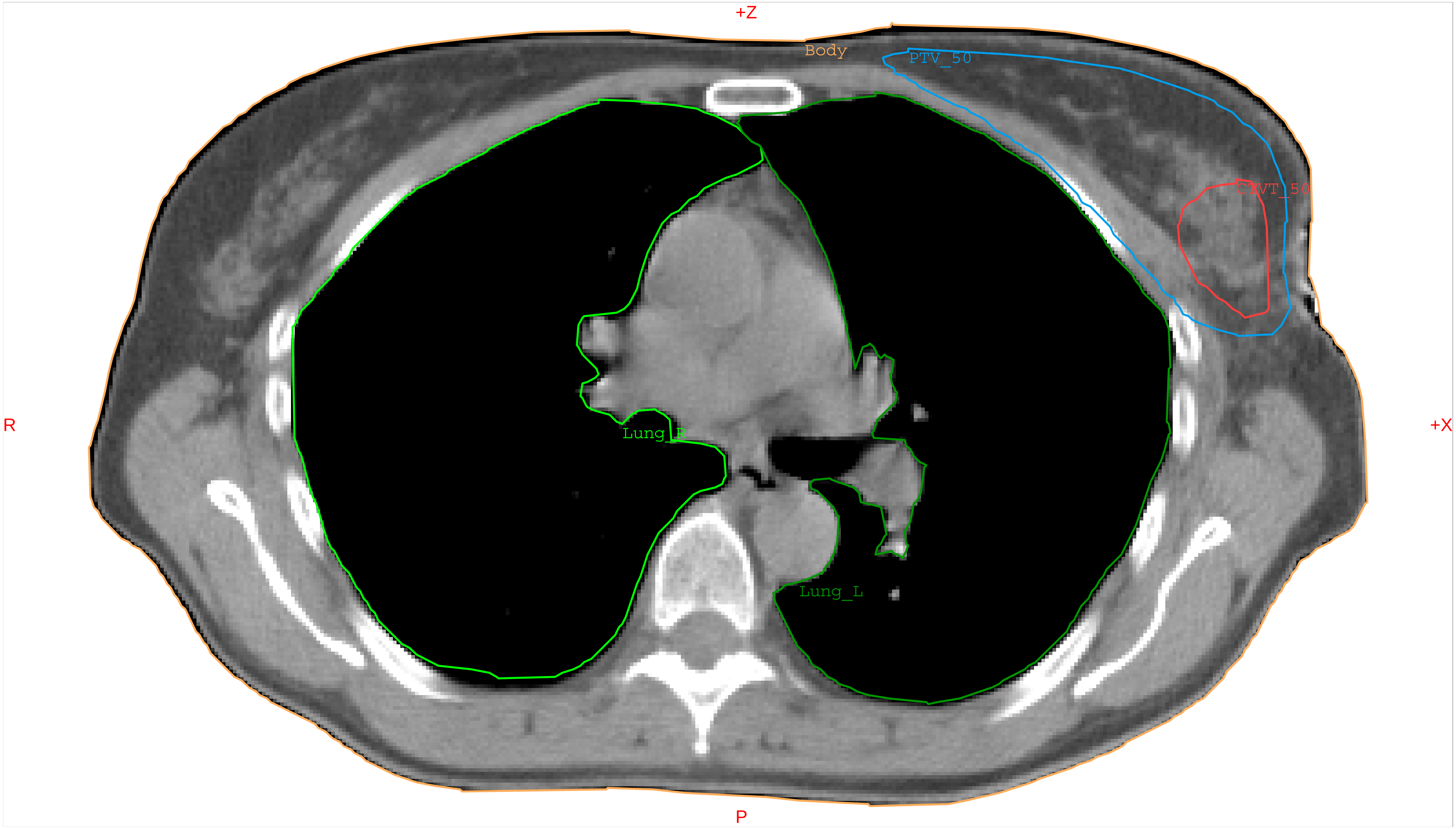

Scale 1:1.3 0 5 10 15 20 25 30 cm

Slice at 4.00 cm (# 30/70)

**Patient name**	med körtelengagemang, bröst ca. I och II
**Patient id**	200609051245
**Case**	PTV mallar
**Plan**	Mallar
**Treatment position**	HFS
**Last saved**	29 Oct 2014 17:44:32

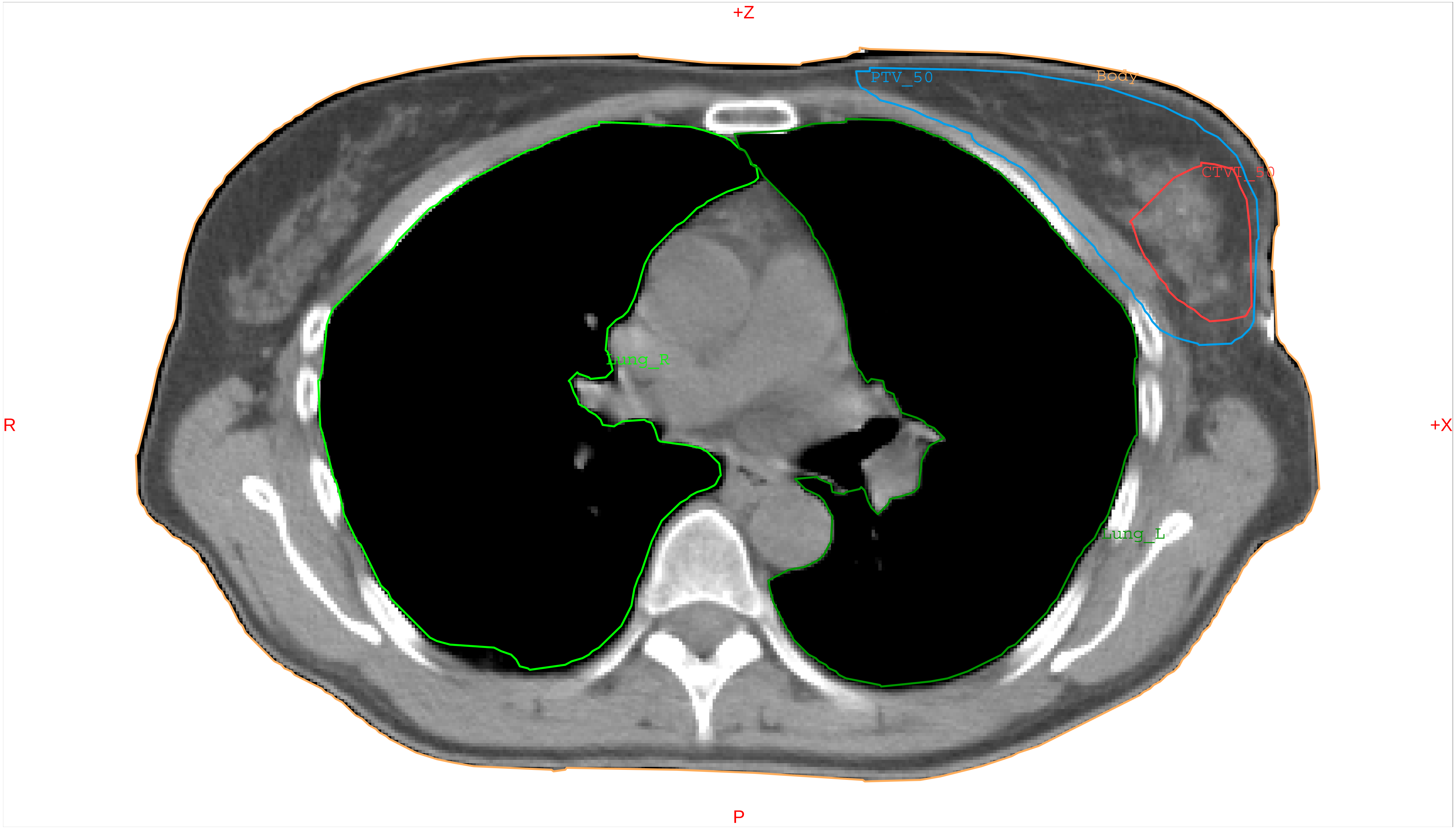

Scale 1:1.4 0 5 10 15 20 25 30 35 cm

Slice at 3.50 cm (# 31/70)

**Patient name**	med körtelengagemang, bröst ca. I och II
**Patient id**	200609051245
**Case**	PTV mallar
**Plan**	Mallar
**Treatment position**	HFS
**Last saved**	29 Oct 2014 17:44:32

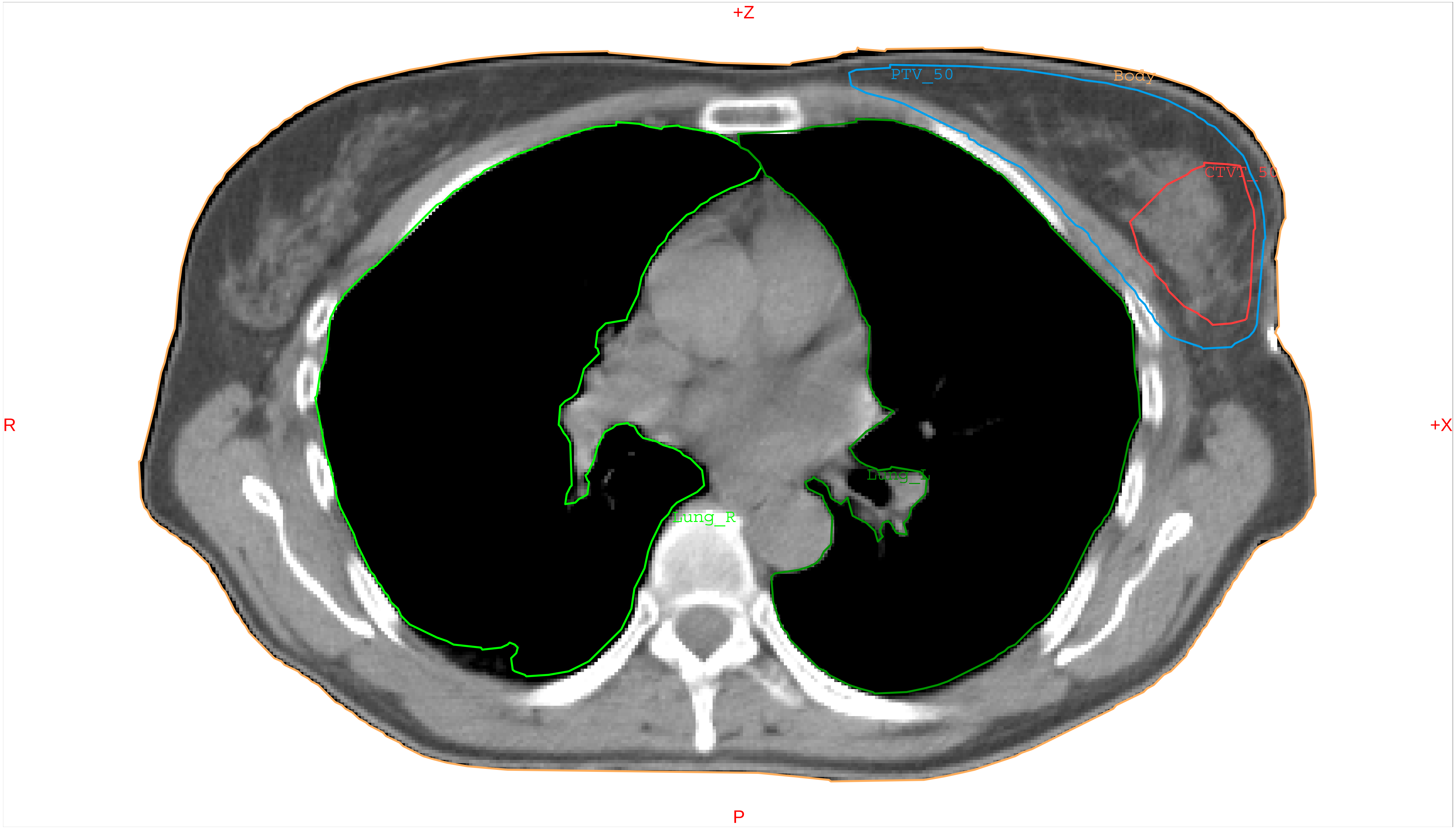

Scale 1:1.4 0 5 10 15 20 25 30 35 cm

Slice at 3.00 cm (# 32/70)

**Patient name**	med körtelengagemang, bröst ca. I och II
**Patient id**	200609051245
**Case**	PTV mallar
**Plan**	Mallar
**Treatment position**	HFS
**Last saved**	29 Oct 2014 17:44:32

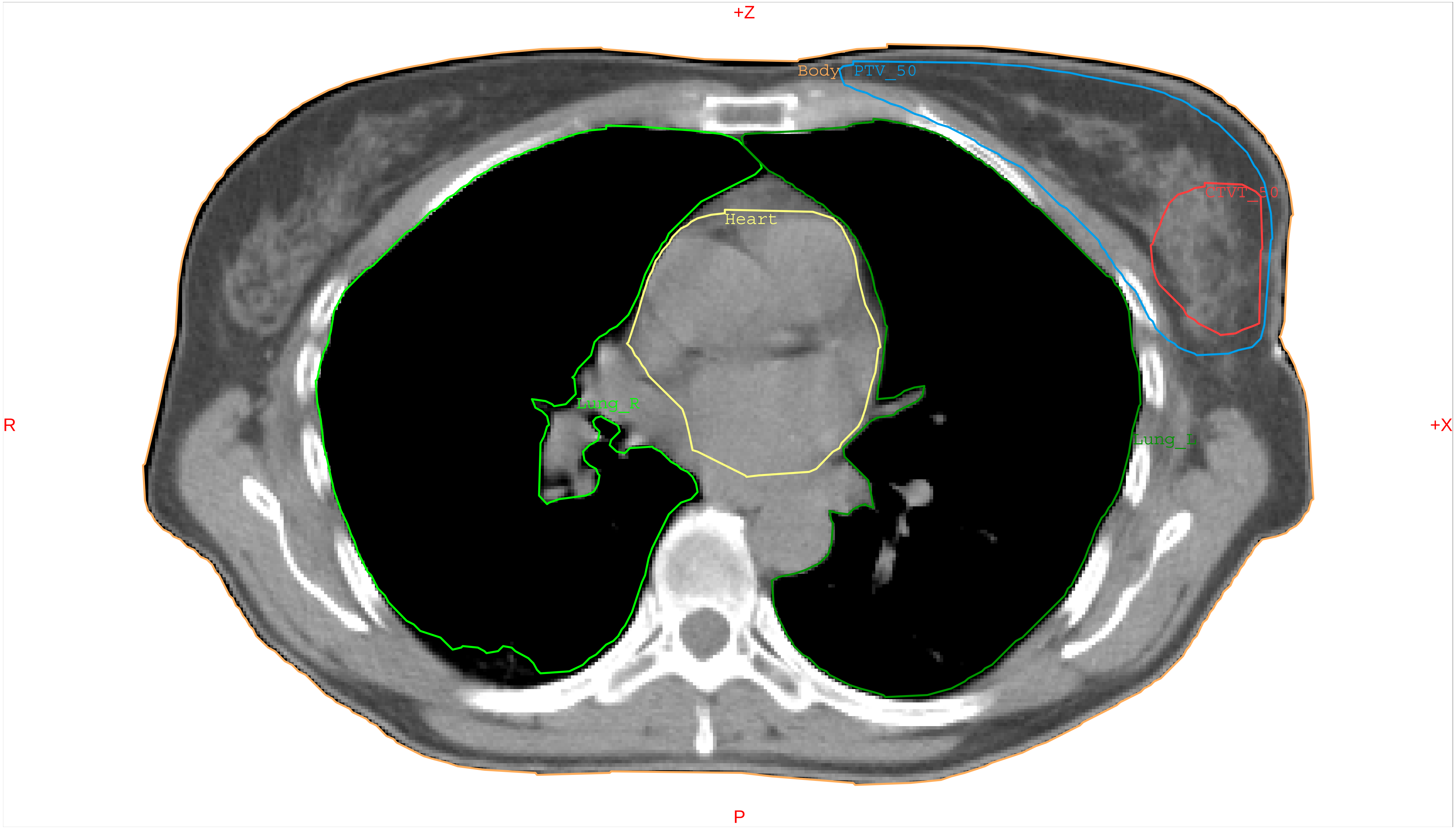

Scale 1:1.4 0 5 10 15 20 25 30 35 cm

Slice at 2.50 cm (# 33/70)

**Patient name**	med körtelengagemang, bröst ca. I och II
**Patient id**	200609051245
**Case**	PTV mallar
**Plan**	Mallar
**Treatment position**	HFS
**Last saved**	29 Oct 2014 17:44:32

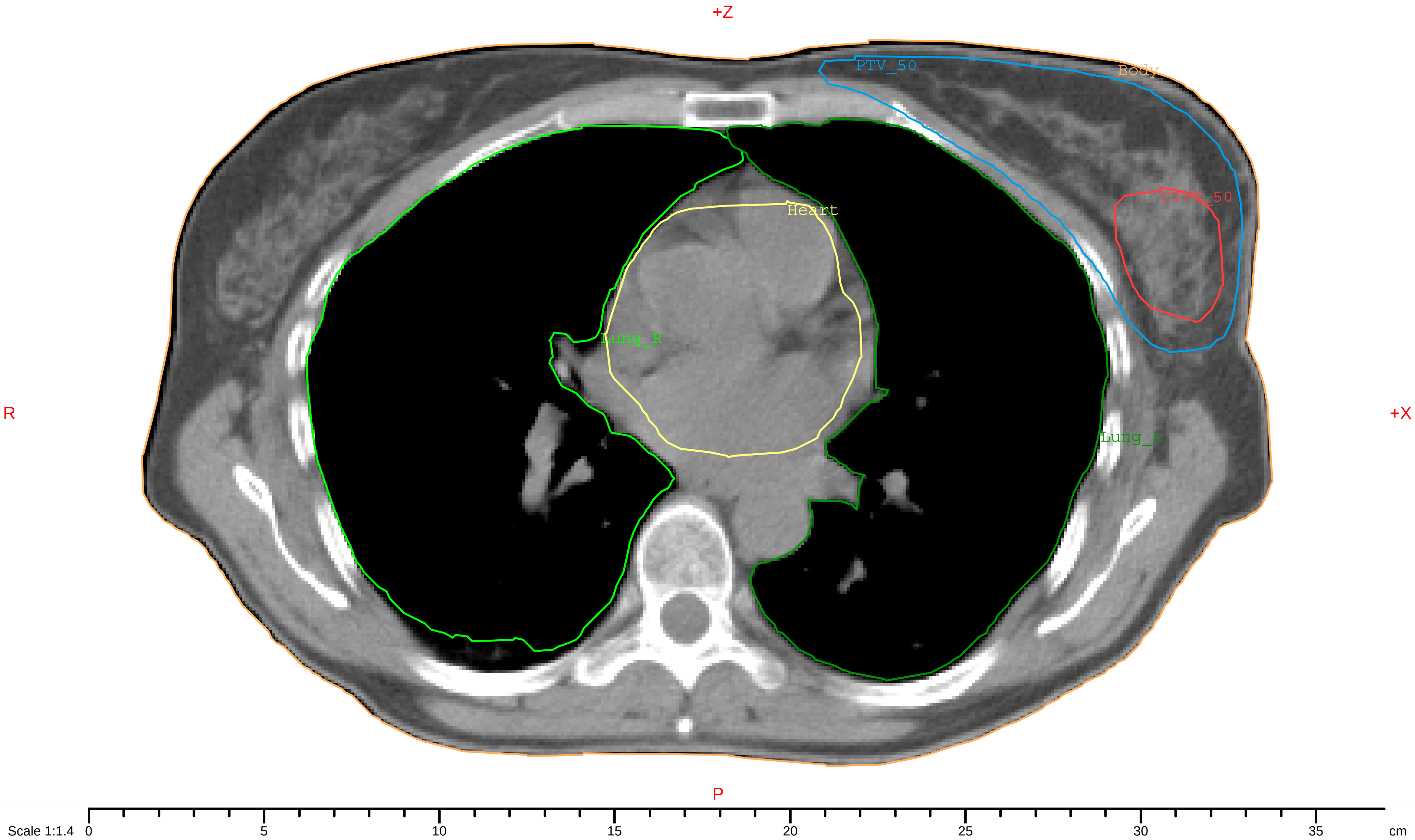

Slice at 2.00 cm (# 34/70)

**Patient name**	med körtelengagemang, bröst ca. I och II
**Patient id**	200609051245
**Case**	PTV mallar
**Plan**	Mallar
**Treatment position**	HFS
**Last saved**	29 Oct 2014 17:44:32

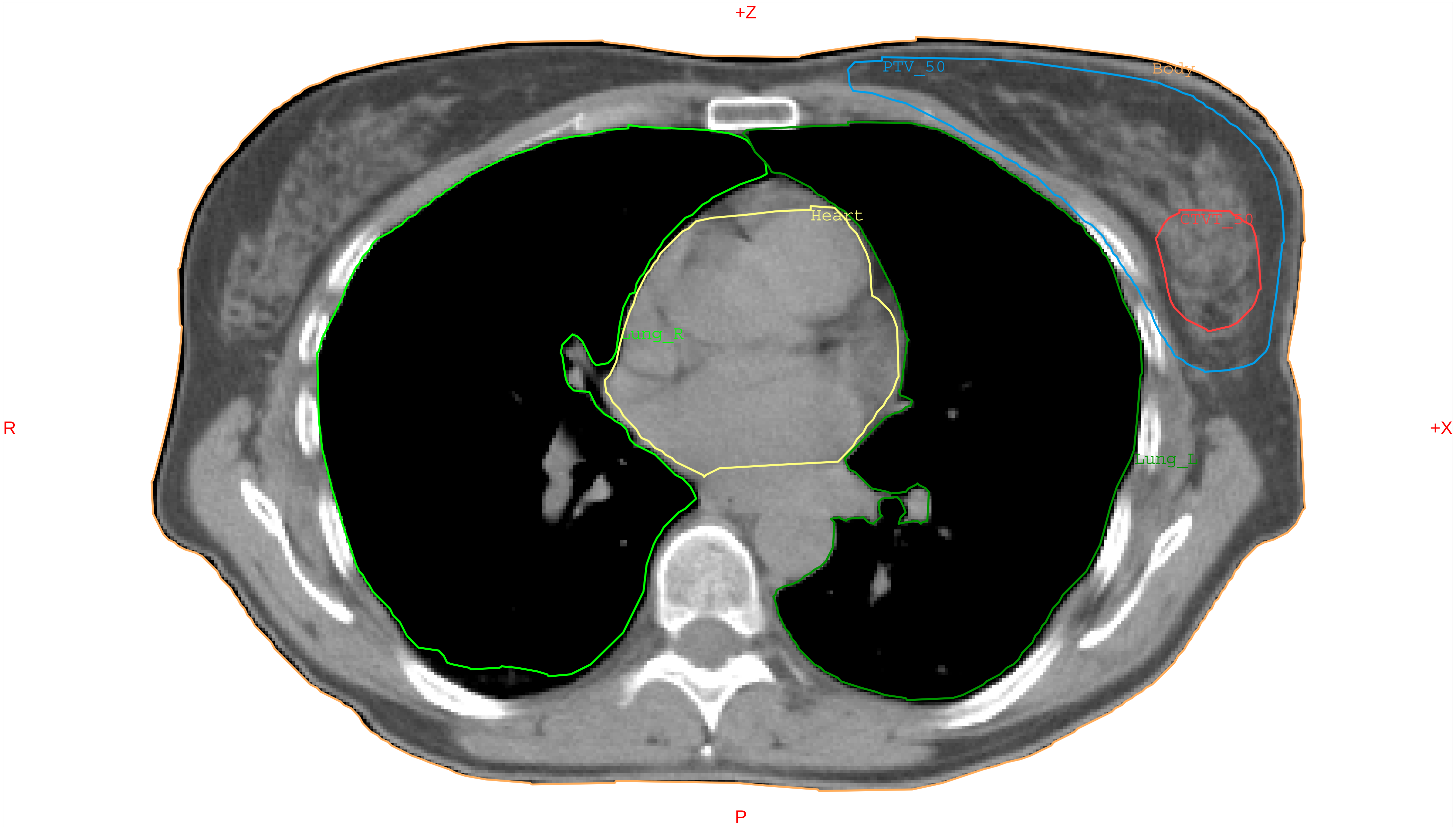

Scale 1:1.4 0 5 10 15 20 25 30 35 cm

Slice at 1.50 cm (# 35/70)

**Patient name**	med körtelengagemang, bröst ca. I och II
**Patient id**	200609051245
**Case**	PTV mallar
**Plan**	Mallar
**Treatment position**	HFS
**Last saved**	29 Oct 2014 17:44:32

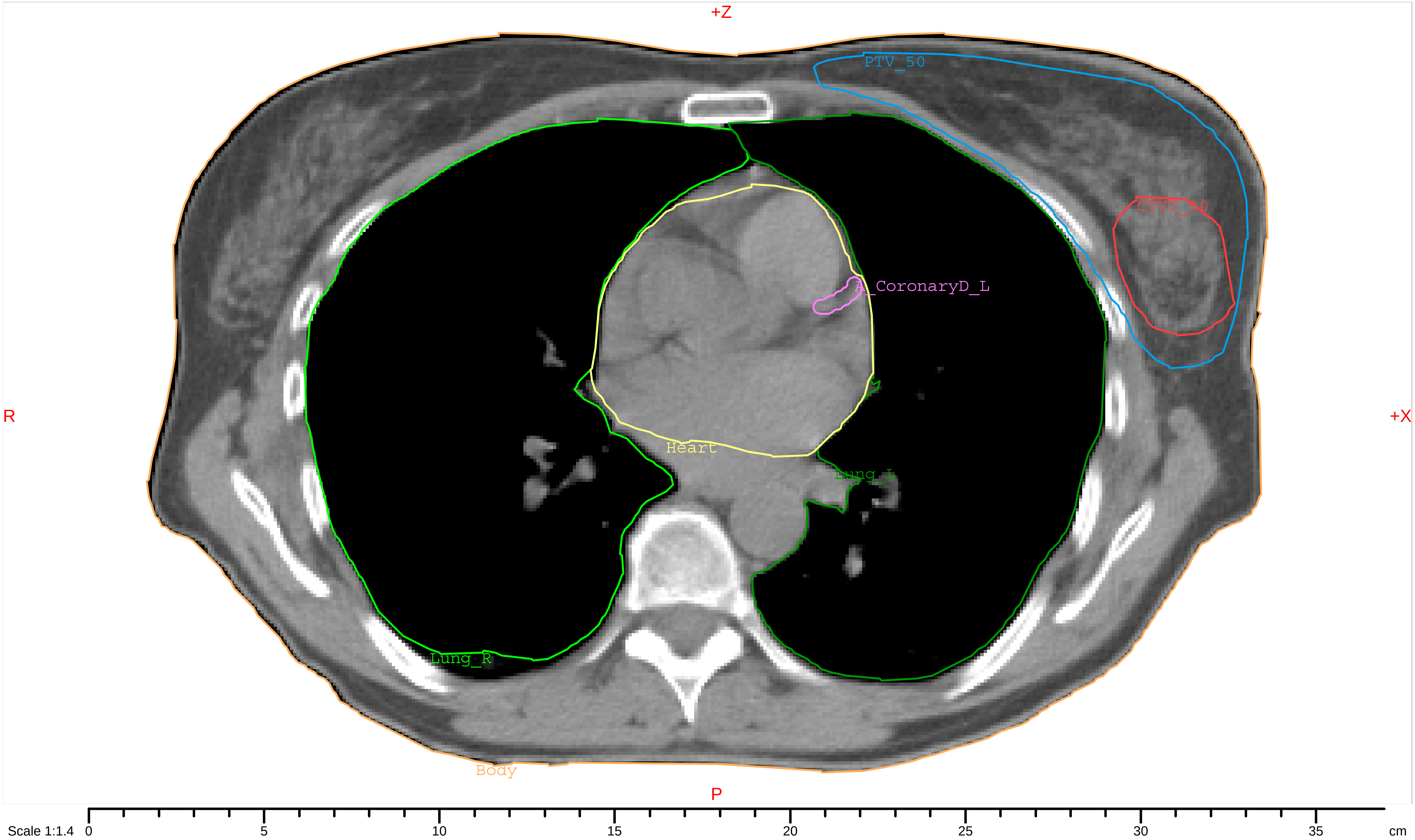

Slice at 1.00 cm (# 36/70)

**Patient name**	med körtelengagemang, bröst ca. I och II
**Patient id**	200609051245
**Case**	PTV mallar
**Plan**	Mallar
**Treatment position**	HFS
**Last saved**	29 Oct 2014 17:44:32

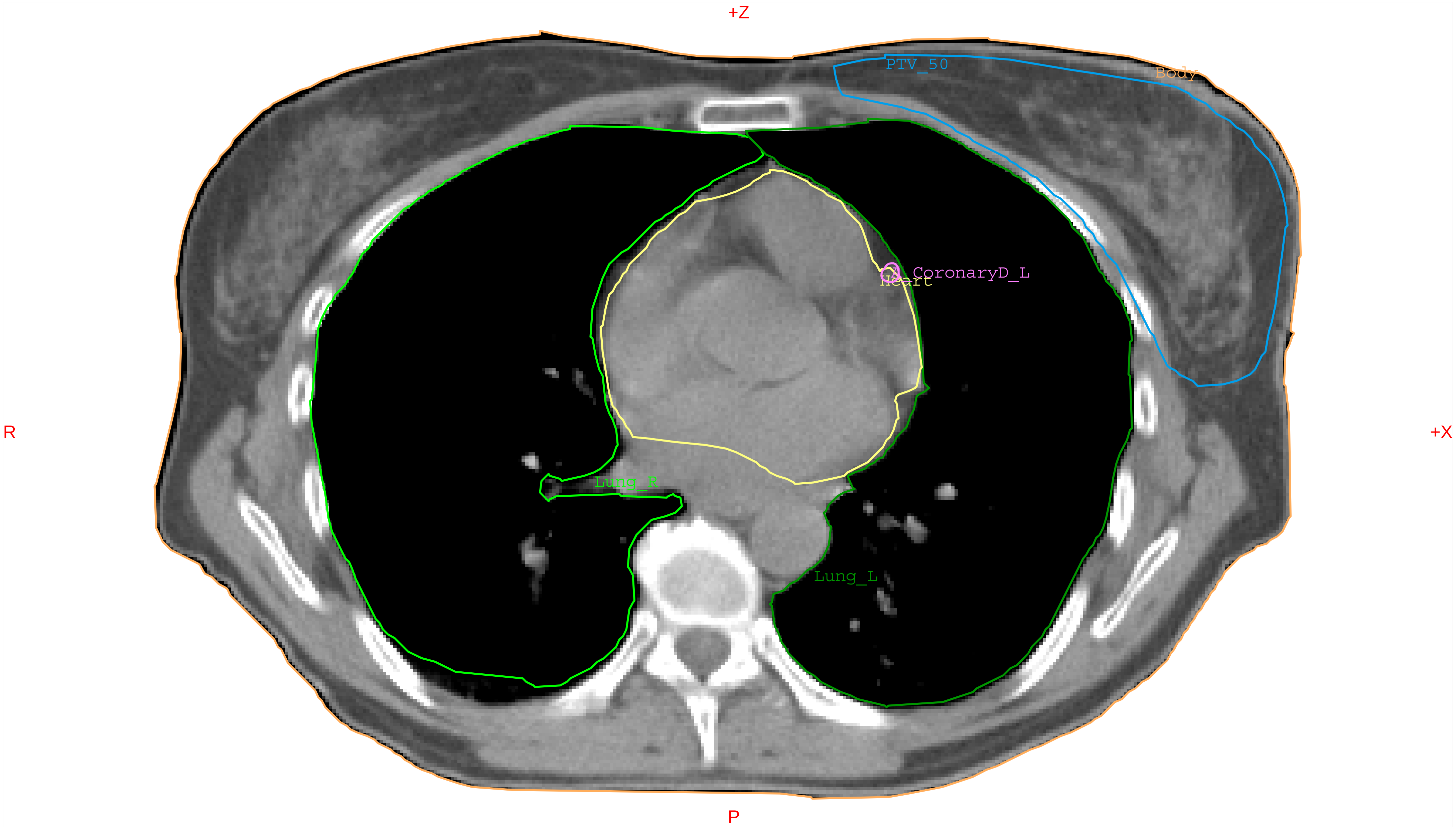

Scale 1:1.4 0 5 10 15 20 25 30 35 cm

Slice at 0.50 cm (# 37/70)

**Patient name**	med körtelengagemang, bröst ca. I och II
**Patient id**	200609051245
**Case**	PTV mallar
**Plan**	Mallar
**Treatment position**	HFS
**Last saved**	29 Oct 2014 17:44:32

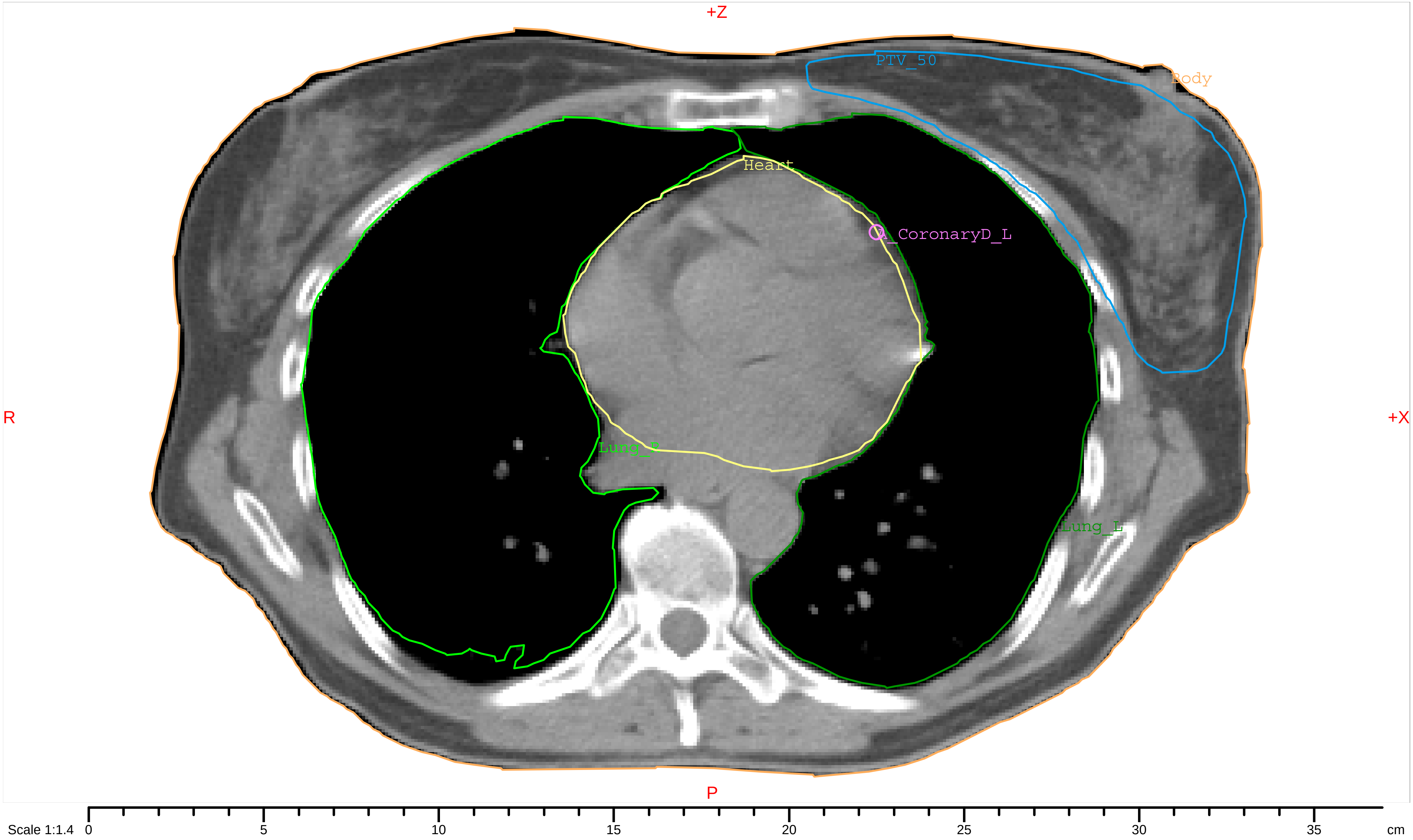

**Patient name**	med körtelengagemang, bröst ca. I och II
**Patient id**	200609051245
**Case**	PTV mallar
**Plan**	Mallar
**Treatment position**	HFS
**Last saved**	29 Oct 2014 17:44:32

Slice at 0.00 cm (# 38/70)

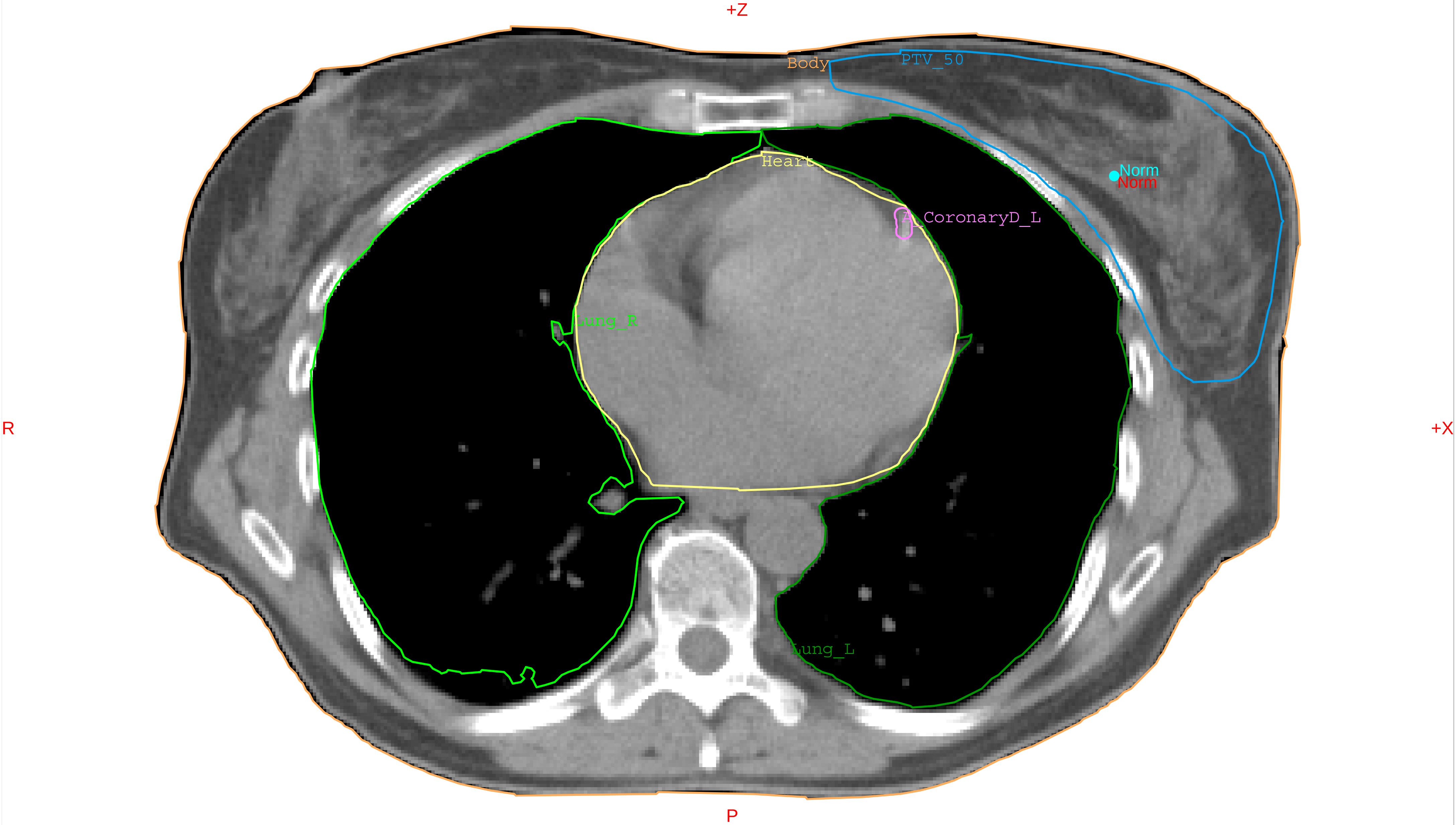

Scale 1:1.4 0 5 10 15 20 25 30 35 cm

Slice at -0.50 cm (# 39/70)

**Patient name**	med körtelengagemang, bröst ca. I och II
**Patient id**	200609051245
**Case**	PTV mallar
**Plan**	Mallar
**Treatment position**	HFS
**Last saved**	29 Oct 2014 17:44:32

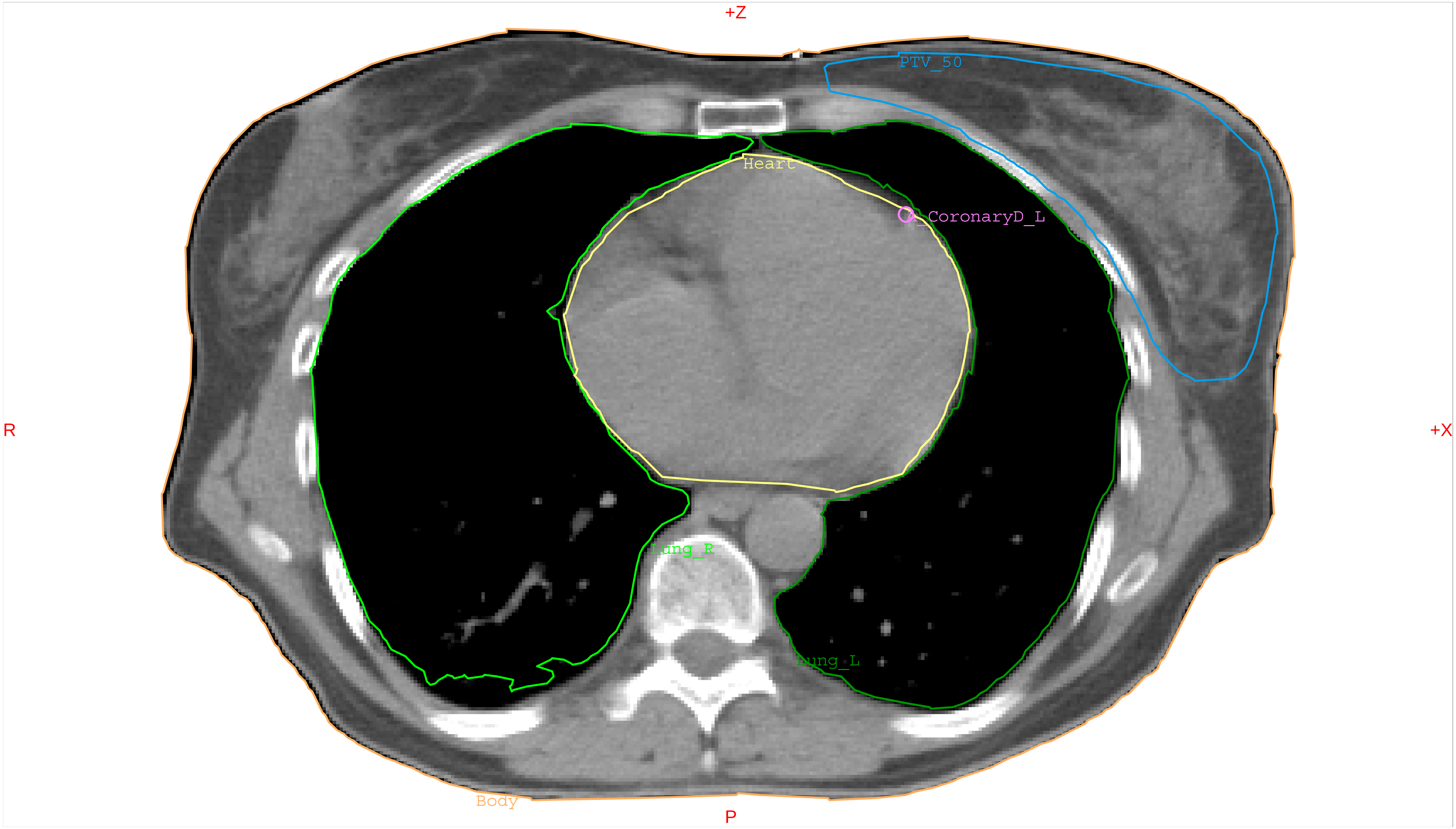

Scale 1:1.4 0 5 10 15 20 25 30 35 cm

**Patient name**	med körtelengagemang, bröst ca. I och II
**Patient id**	200609051245
**Case**	PTV mallar
**Plan**	Mallar
**Treatment position**	HFS
**Last saved**	29 Oct 2014 17:44:32

Slice at -1.00 cm (# 40/70)

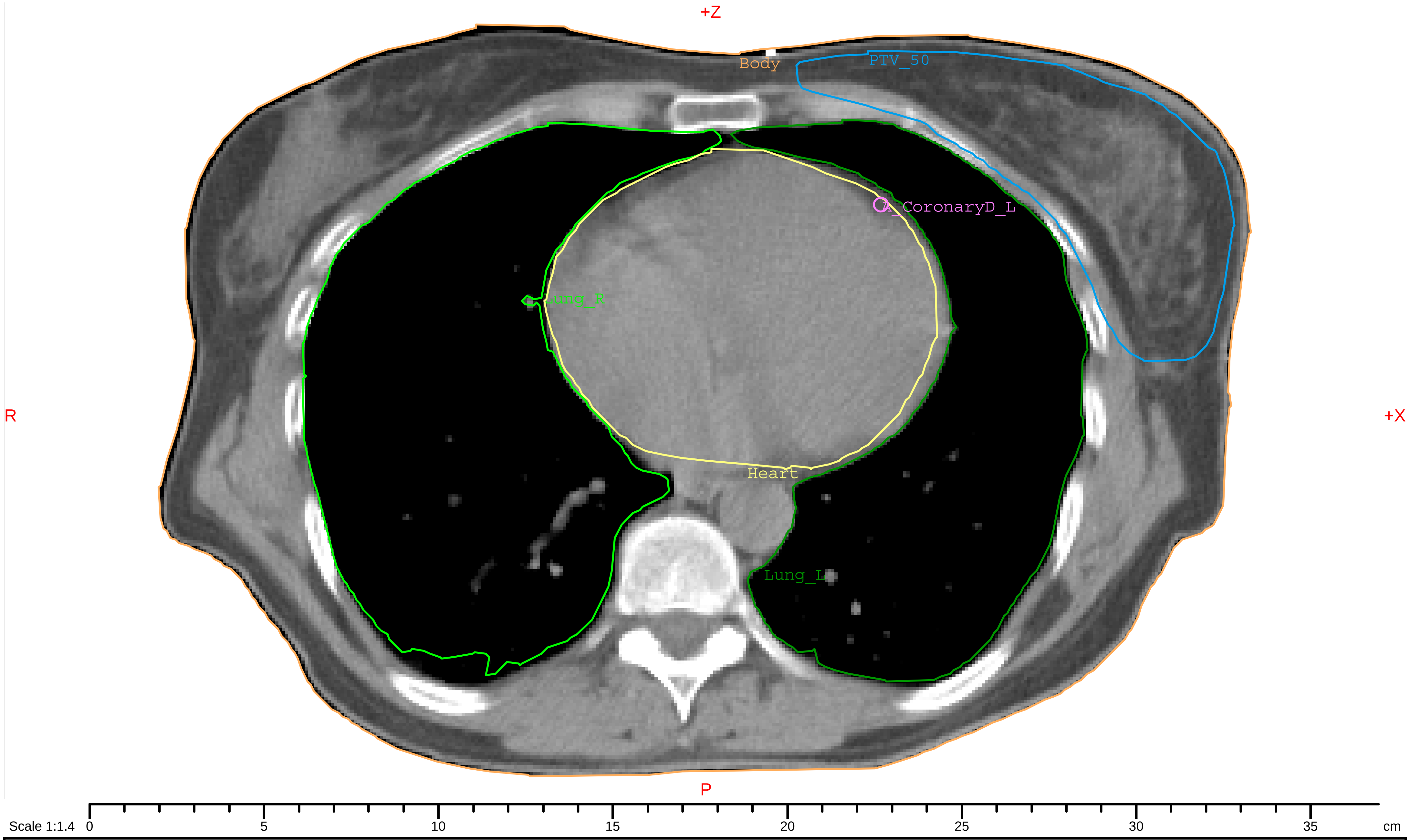

Slice at -1.50 cm (# 41/70)

**Patient name**	med körtelengagemang, bröst ca. I och II
**Patient id**	200609051245
**Case**	PTV mallar
**Plan**	Mallar
**Treatment position**	HFS
**Last saved**	29 Oct 2014 17:44:32

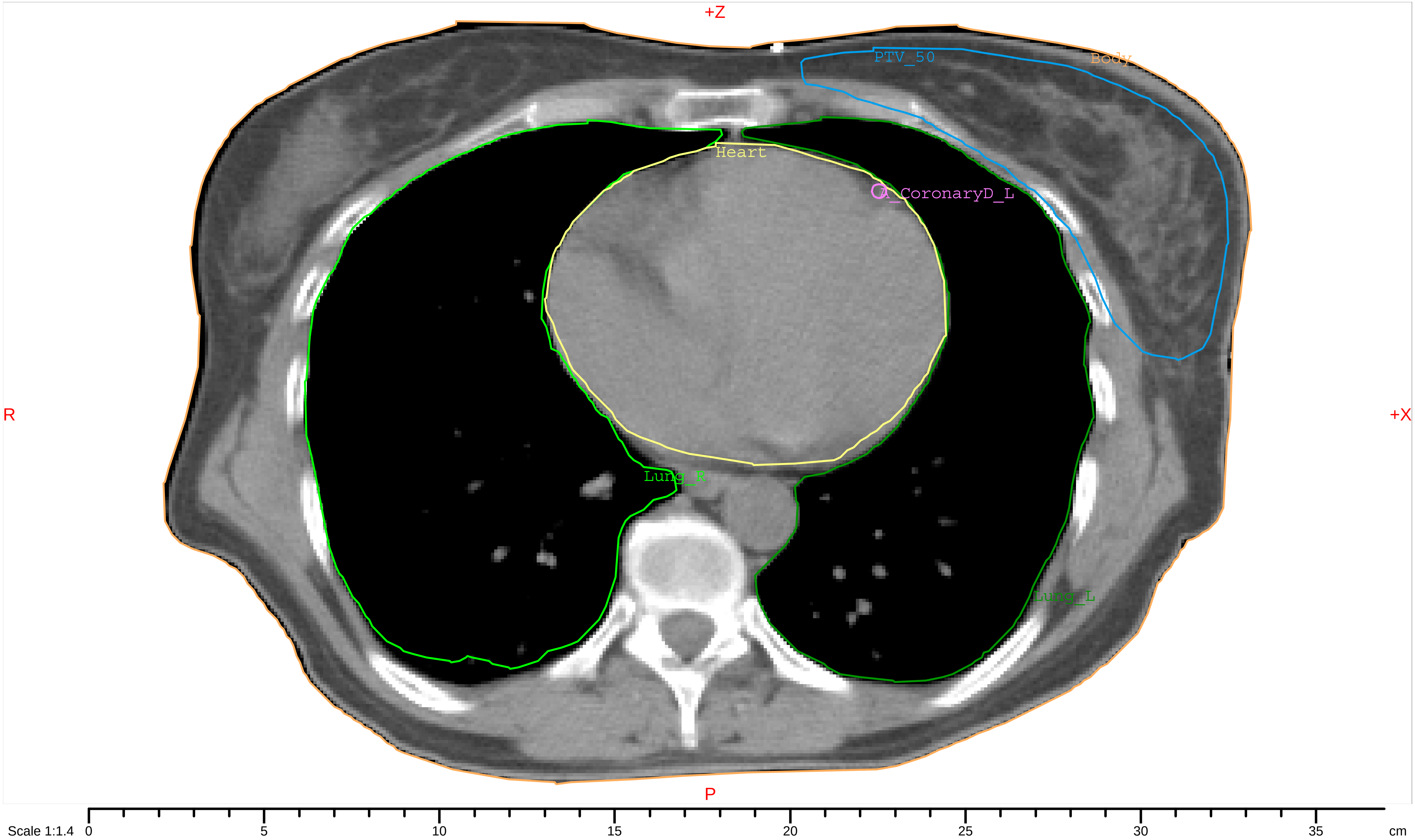

Slice at -2.00 cm (# 42/70)

**Patient name**	med körtelengagemang, bröst ca. I och II
**Patient id**	200609051245
**Case**	PTV mallar
**Plan**	Mallar
**Treatment position**	HFS
**Last saved**	29 Oct 2014 17:44:32

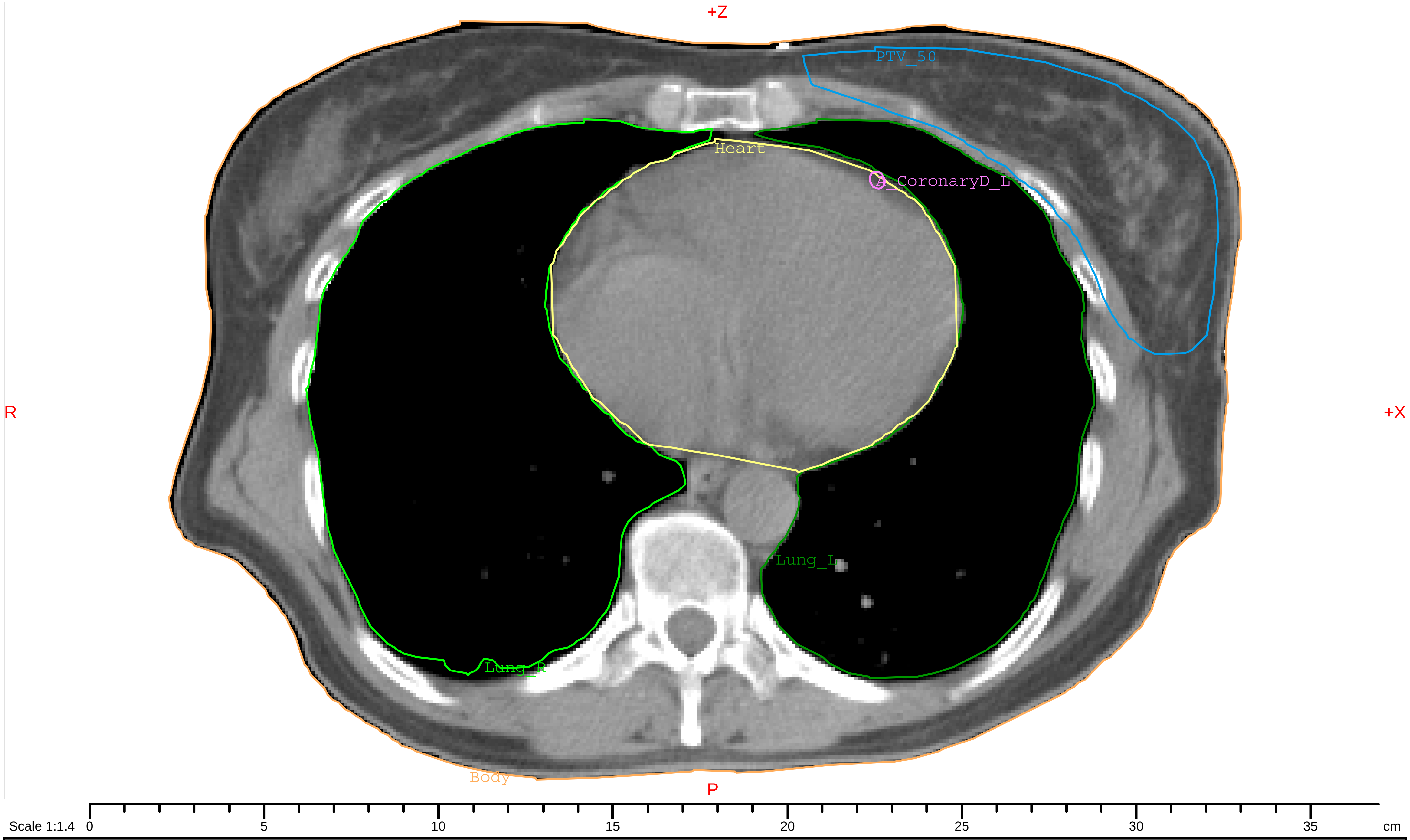

Slice at -2.50 cm (# 43/70)

**Patient name**	med körtelengagemang, bröst ca. I och II
**Patient id**	200609051245
**Case**	PTV mallar
**Plan**	Mallar
**Treatment position**	HFS
**Last saved**	29 Oct 2014 17:44:32

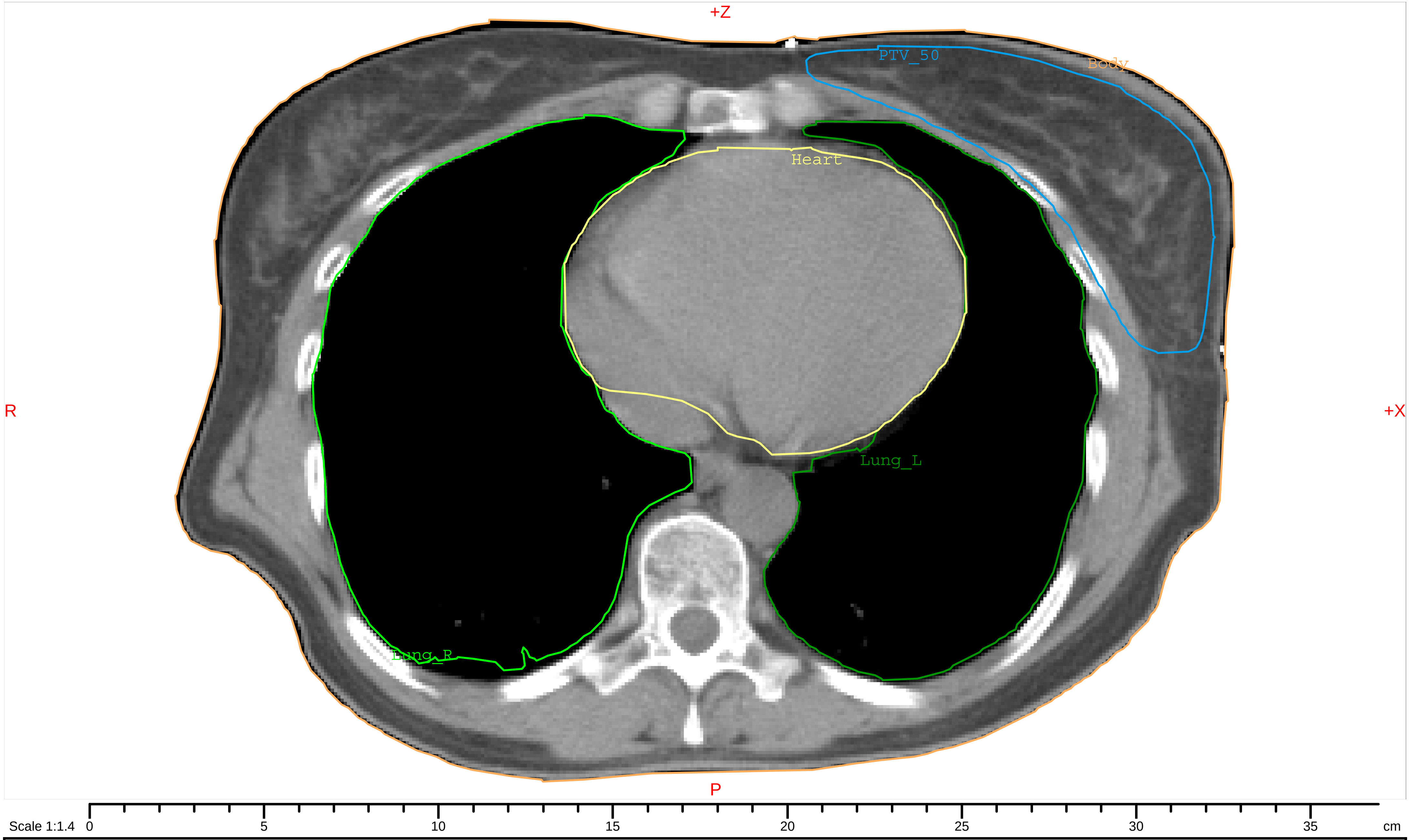

Slice at -3.00 cm (# 44/70)

**Patient name**	med körtelengagemang, bröst ca. I och II
**Patient id**	200609051245
**Case**	PTV mallar
**Plan**	Mallar
**Treatment position**	HFS
**Last saved**	29 Oct 2014 17:44:32

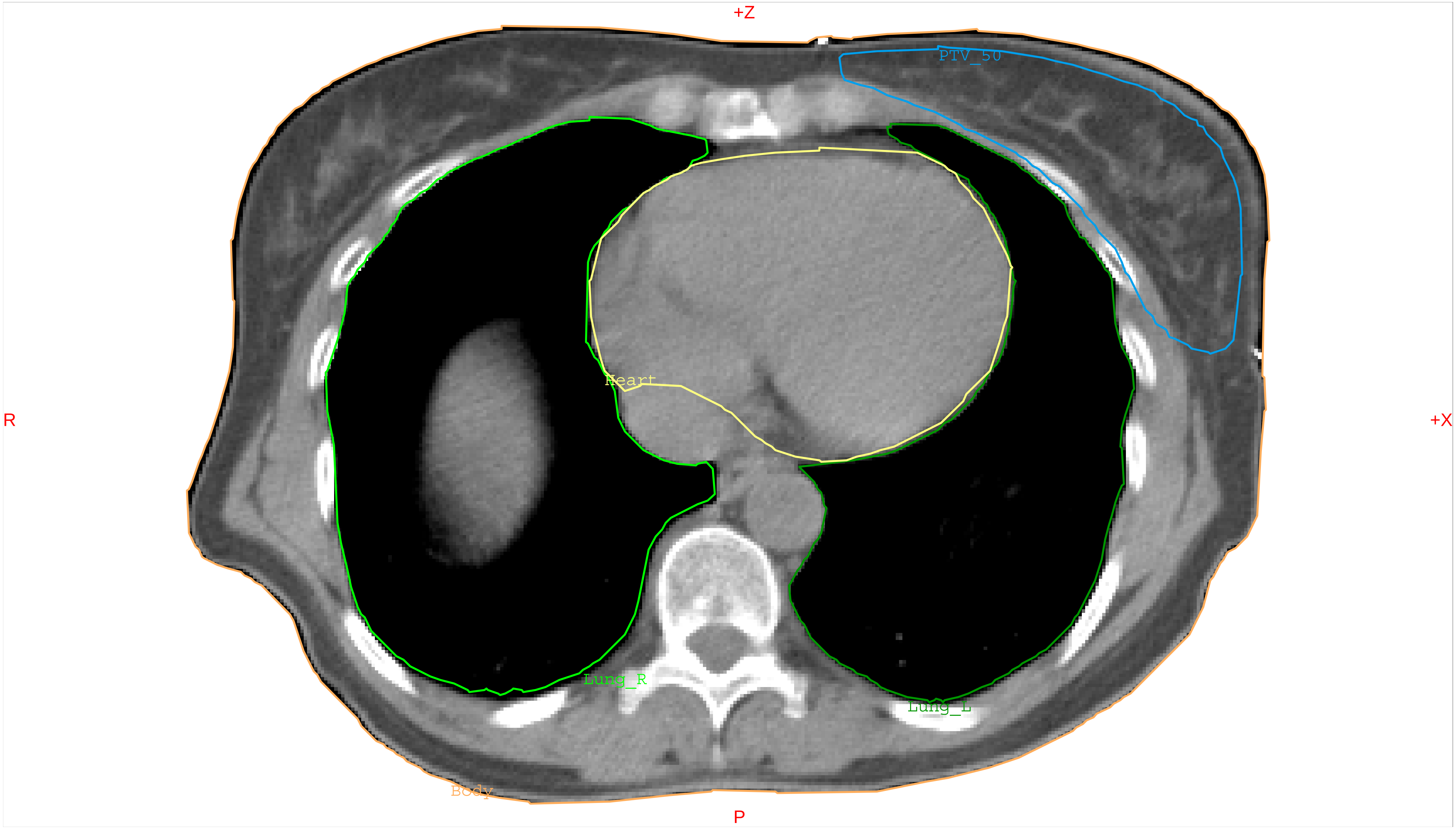

Scale 1:1.4   0   5   10   15   20   25   30   35   cm

Slice at -3.50 cm (# 45/70)

**Patient name**	med körtelengagemang, bröst ca. I och II
**Patient id**	200609051245
**Case**	PTV mallar
**Plan**	Mallar
**Treatment position**	HFS
**Last saved**	29 Oct 2014 17:44:32

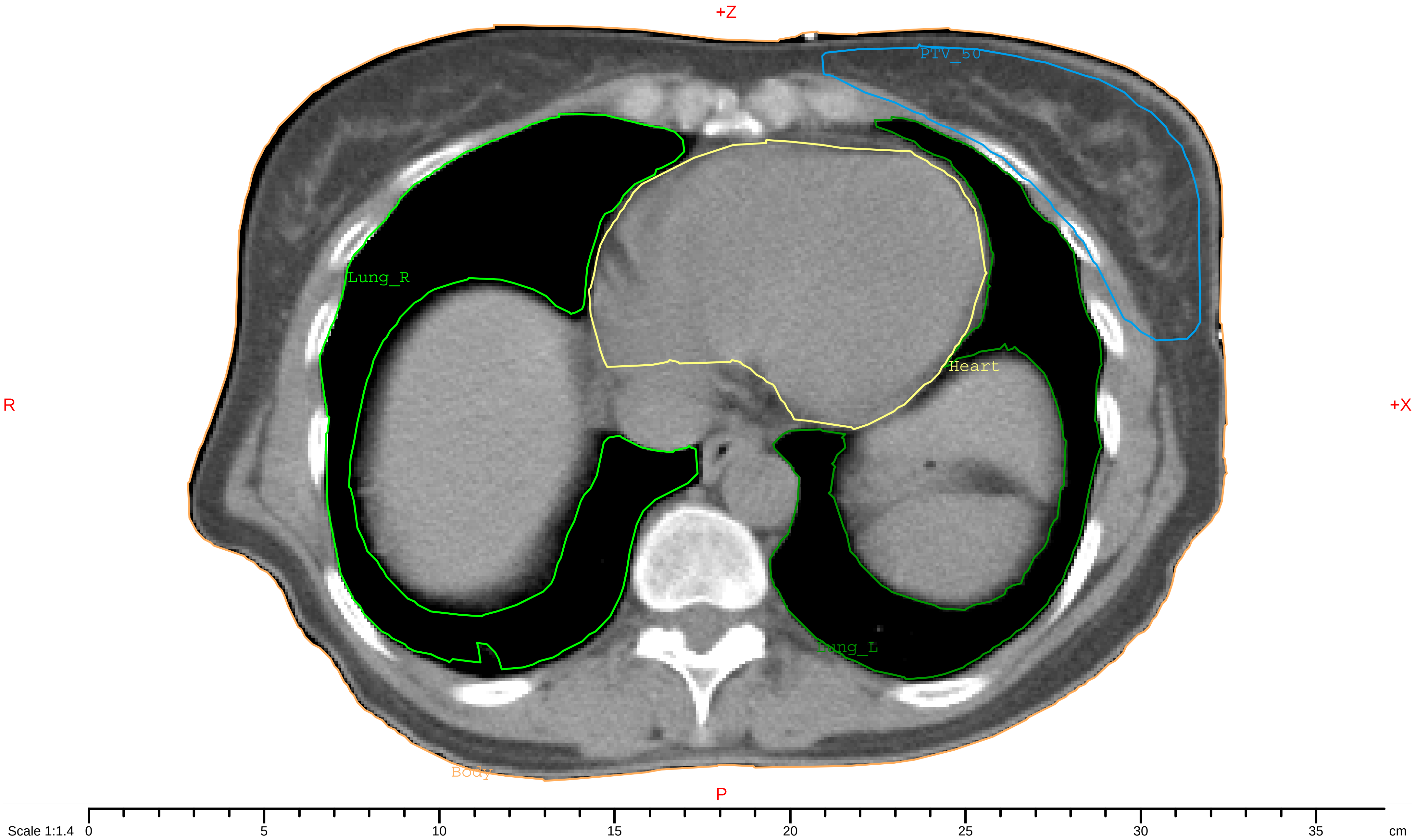

Scale 1:1.4

Slice at -4.00 cm (# 46/70)

**Patient name**	med körtelengagemang, bröst ca. I och II
**Patient id**	200609051245
**Case**	PTV mallar
**Plan**	Mallar
**Treatment position**	HFS
**Last saved**	29 Oct 2014 17:44:32

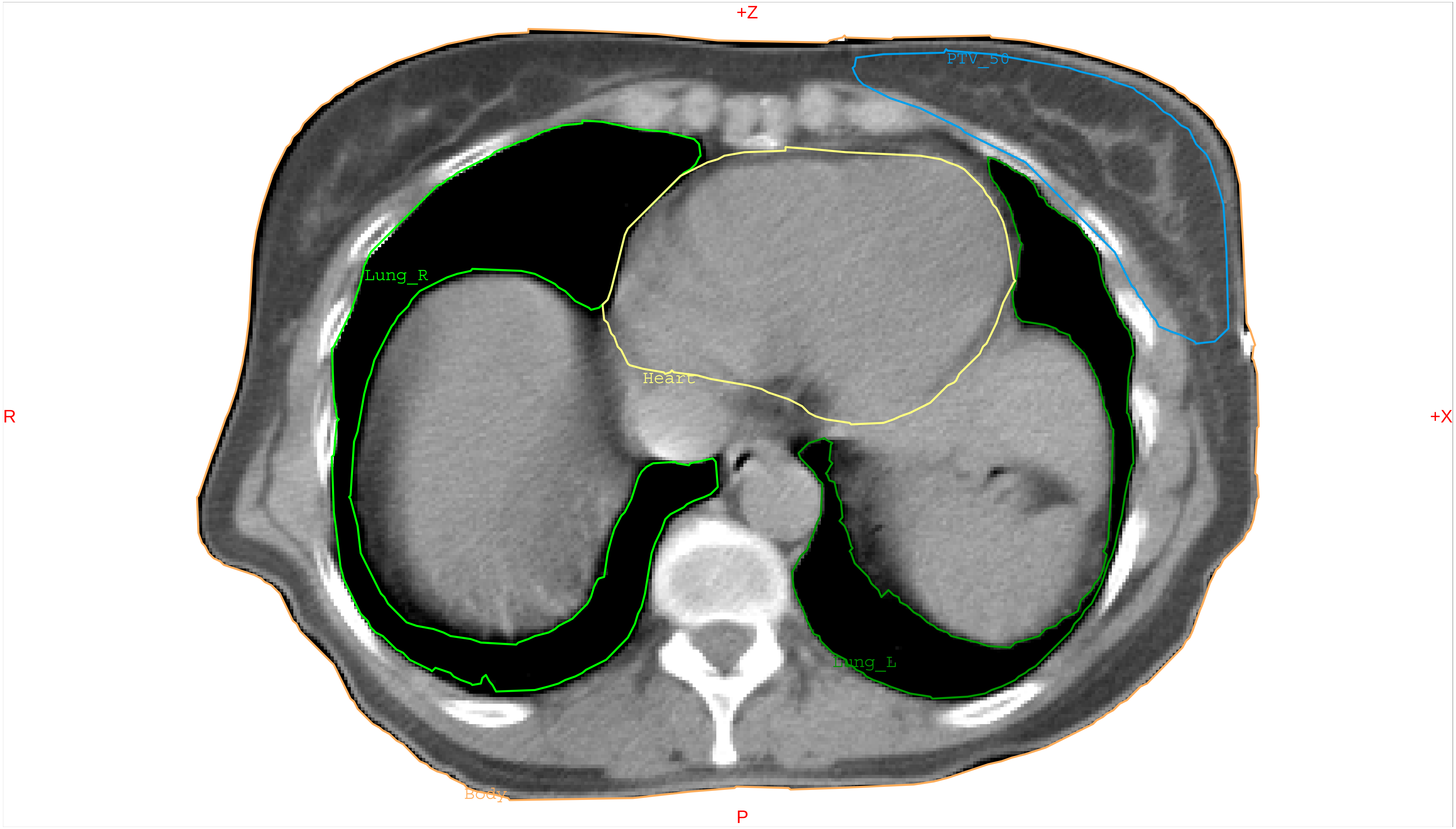

Scale 1:1.4 0 5 10 15 20 25 30 35 cm

Slice at -4.50 cm (# 47/70)

**Patient name**	med körtelengagemang, bröst ca. I och II
**Patient id**	200609051245
**Case**	PTV mallar
**Plan**	Mallar
**Treatment position**	HFS
**Last saved**	29 Oct 2014 17:44:32

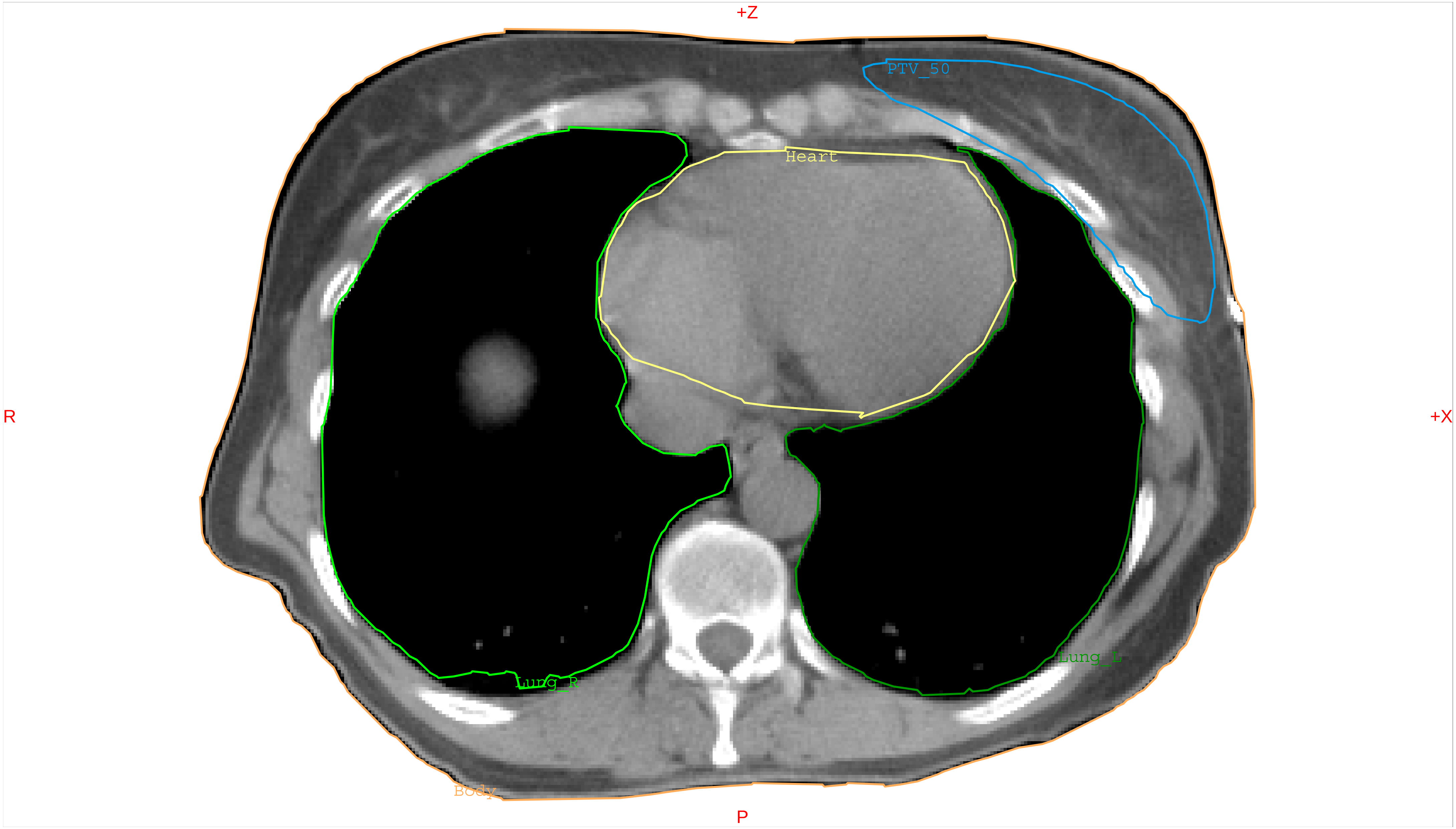

Scale 1:1.4 0 5 10 15 20 25 30 35 cm

Slice at -5.00 cm (# 48/70)

**Patient name**	med körtelengagemang, bröst ca. I och II
**Patient id**	200609051245
**Case**	PTV mallar
**Plan**	Mallar
**Treatment position**	HFS
**Last saved**	29 Oct 2014 17:44:32

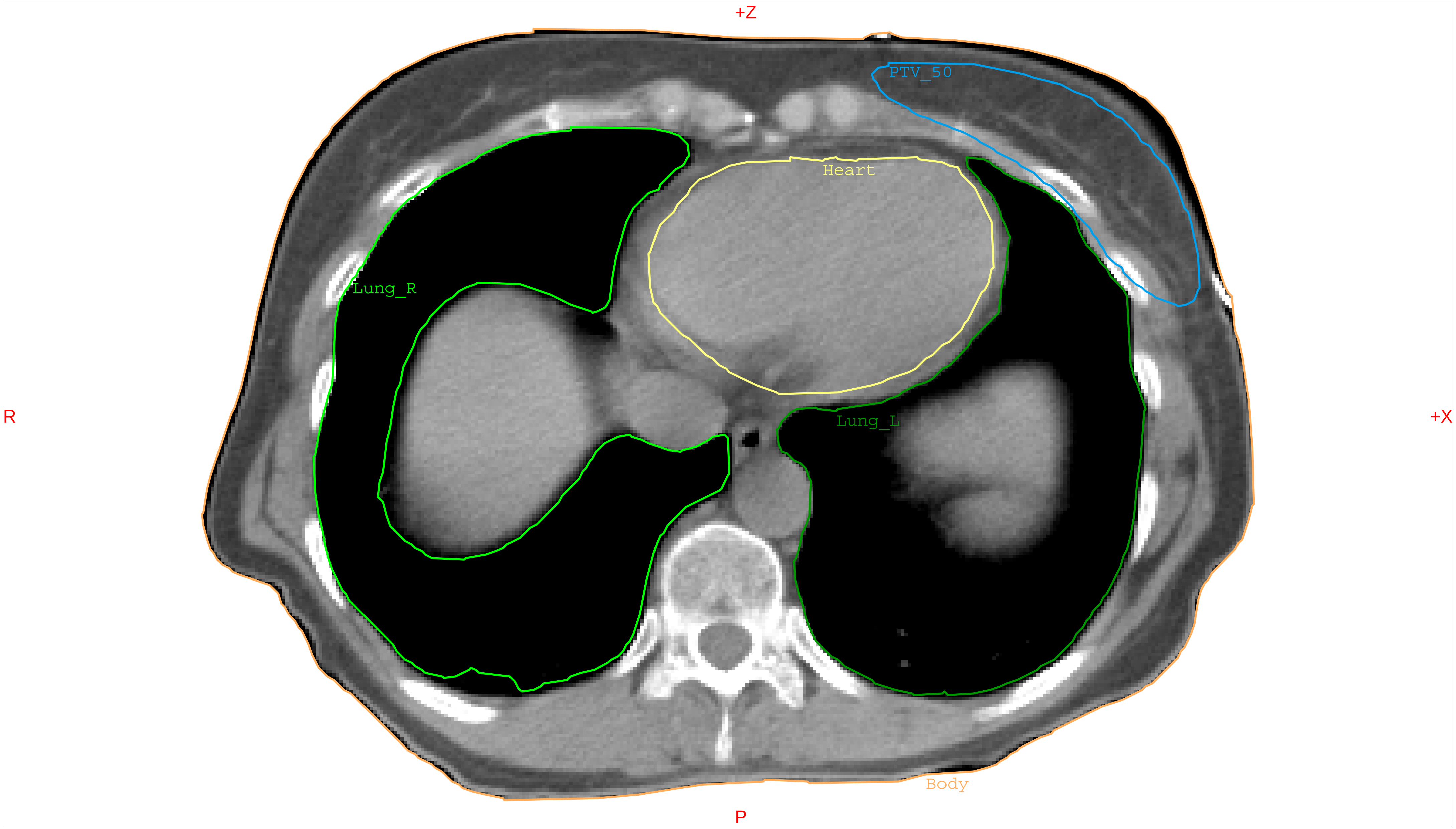

Scale 1:1.4 0 5 10 15 20 25 30 35 cm

Slice at -5.50 cm (# 49/70)

**Patient name**	med körtelengagemang, bröst ca. I och II
**Patient id**	200609051245
**Case**	PTV mallar
**Plan**	Mallar
**Treatment position**	HFS
**Last saved**	29 Oct 2014 17:44:32

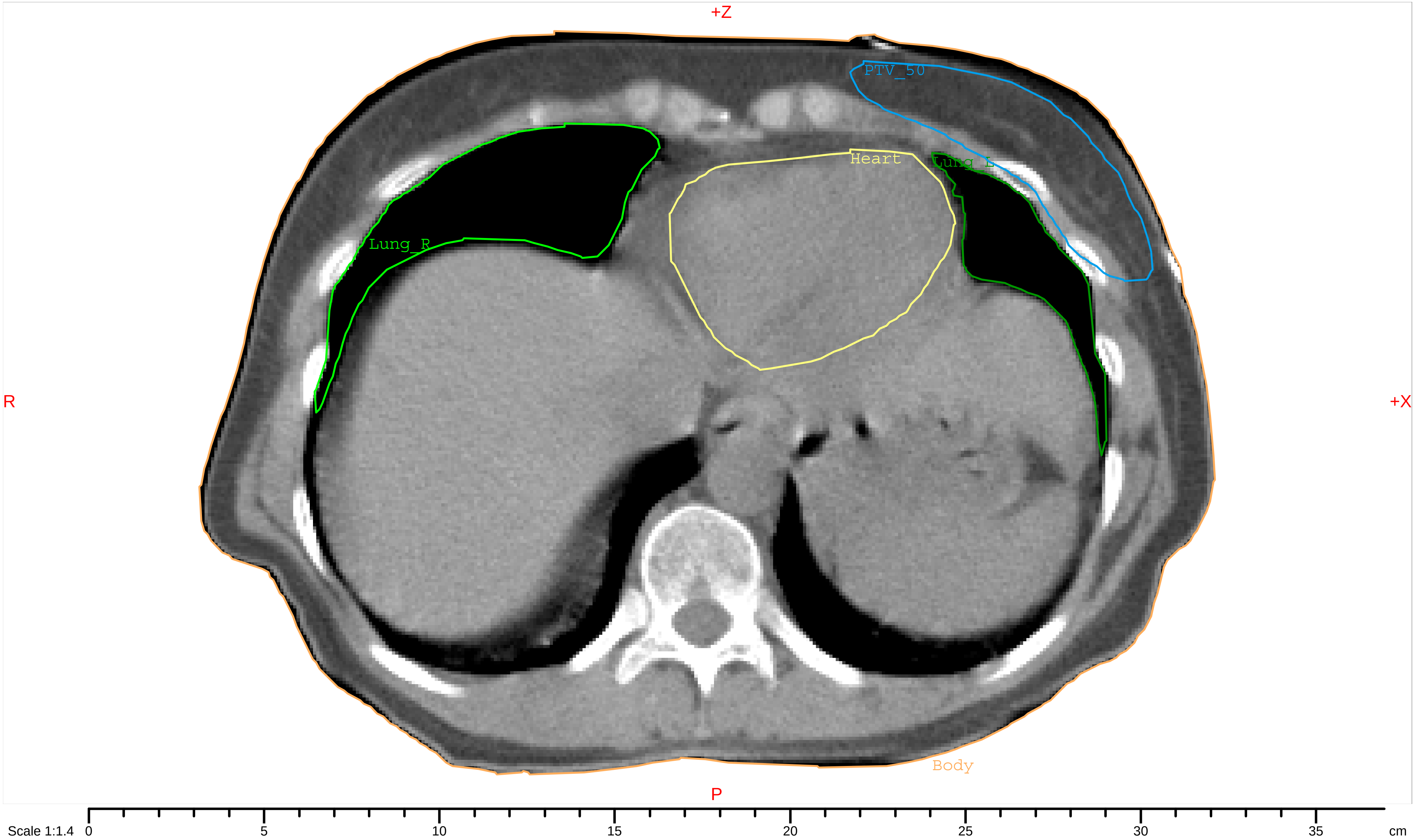

Slice at -6.00 cm (# 50/70)

**Patient name**	med körtelengagemang, bröst ca. I och II
**Patient id**	200609051245
**Case**	PTV mallar
**Plan**	Mallar
**Treatment position**	HFS
**Last saved**	29 Oct 2014 17:44:32

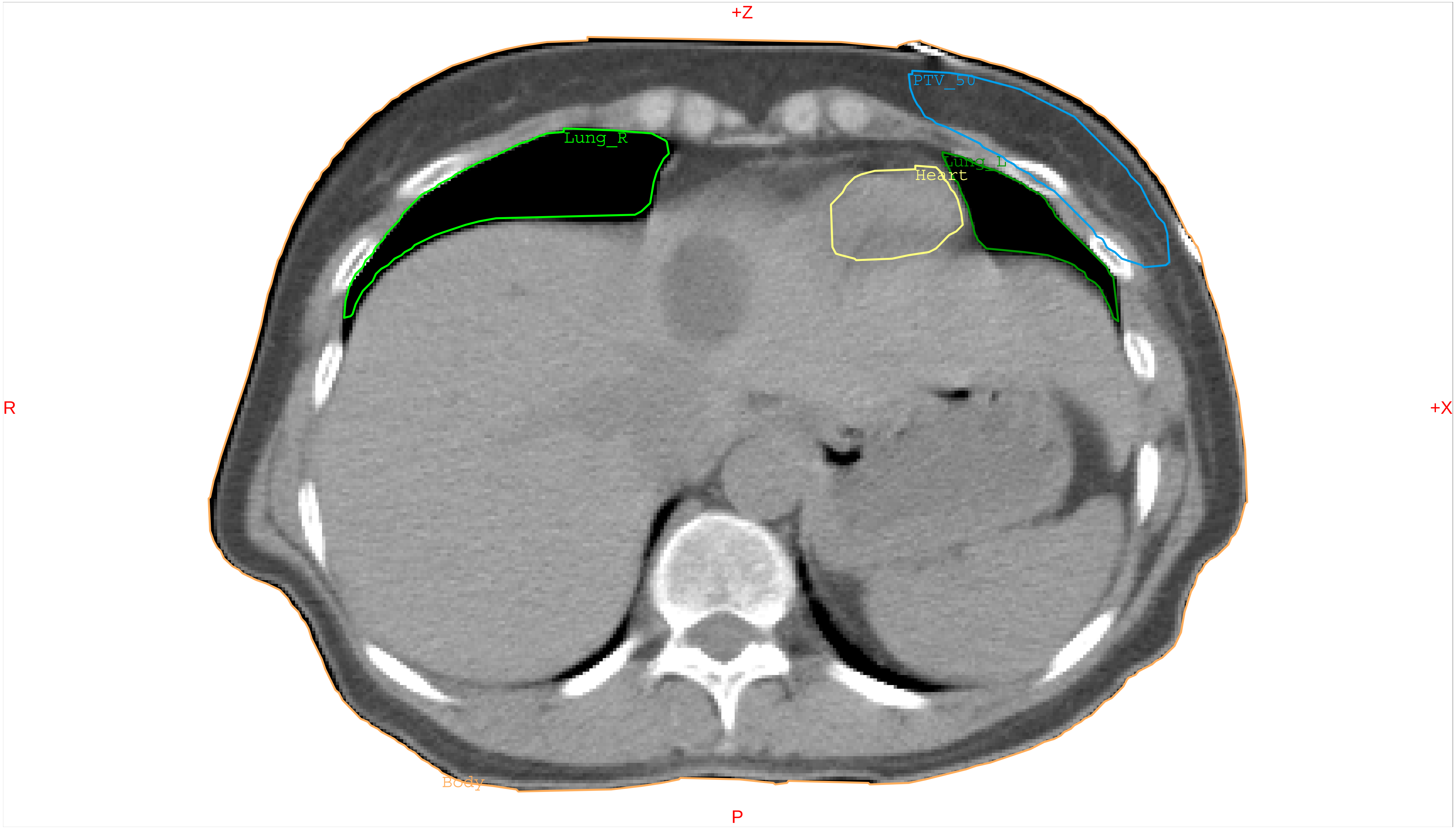

Scale 1:1.4 0 5 10 15 20 25 30 35 cm

Slice at -6.50 cm (# 51/70)

**Patient name**	med körtelengagemang, bröst ca. I och II
**Patient id**	200609051245
**Case**	PTV mallar
**Plan**	Mallar
**Treatment position**	HFS
**Last saved**	29 Oct 2014 17:44:32

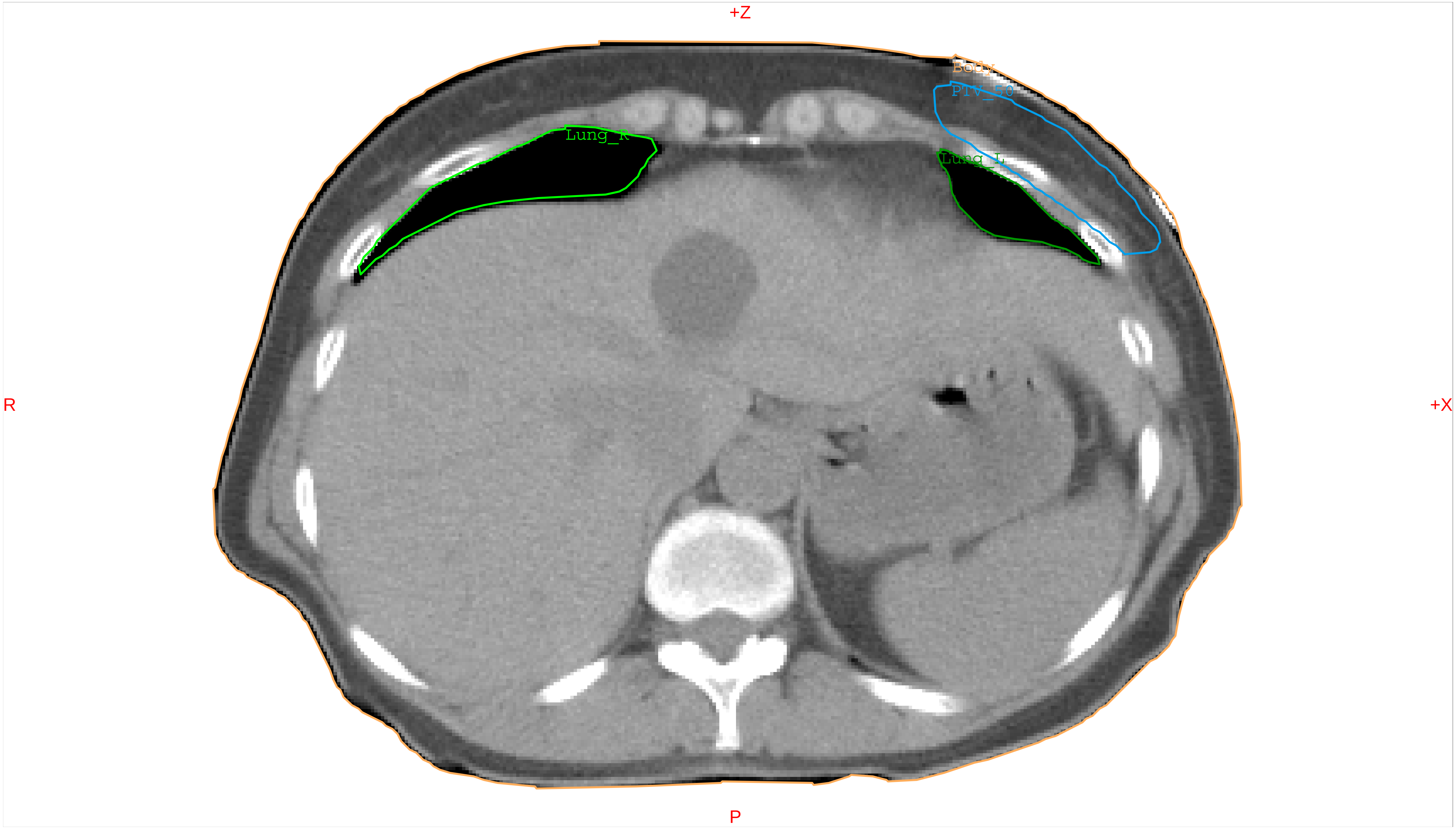

Scale 1:1.4 0 5 10 15 20 25 30 35 cm

Slice at -7.00 cm (# 52/70)

**Patient name**	med körtelengagemang, bröst ca. I och II
**Patient id**	200609051245
**Case**	PTV mallar
**Plan**	Mallar
**Treatment position**	HFS
**Last saved**	29 Oct 2014 17:44:32

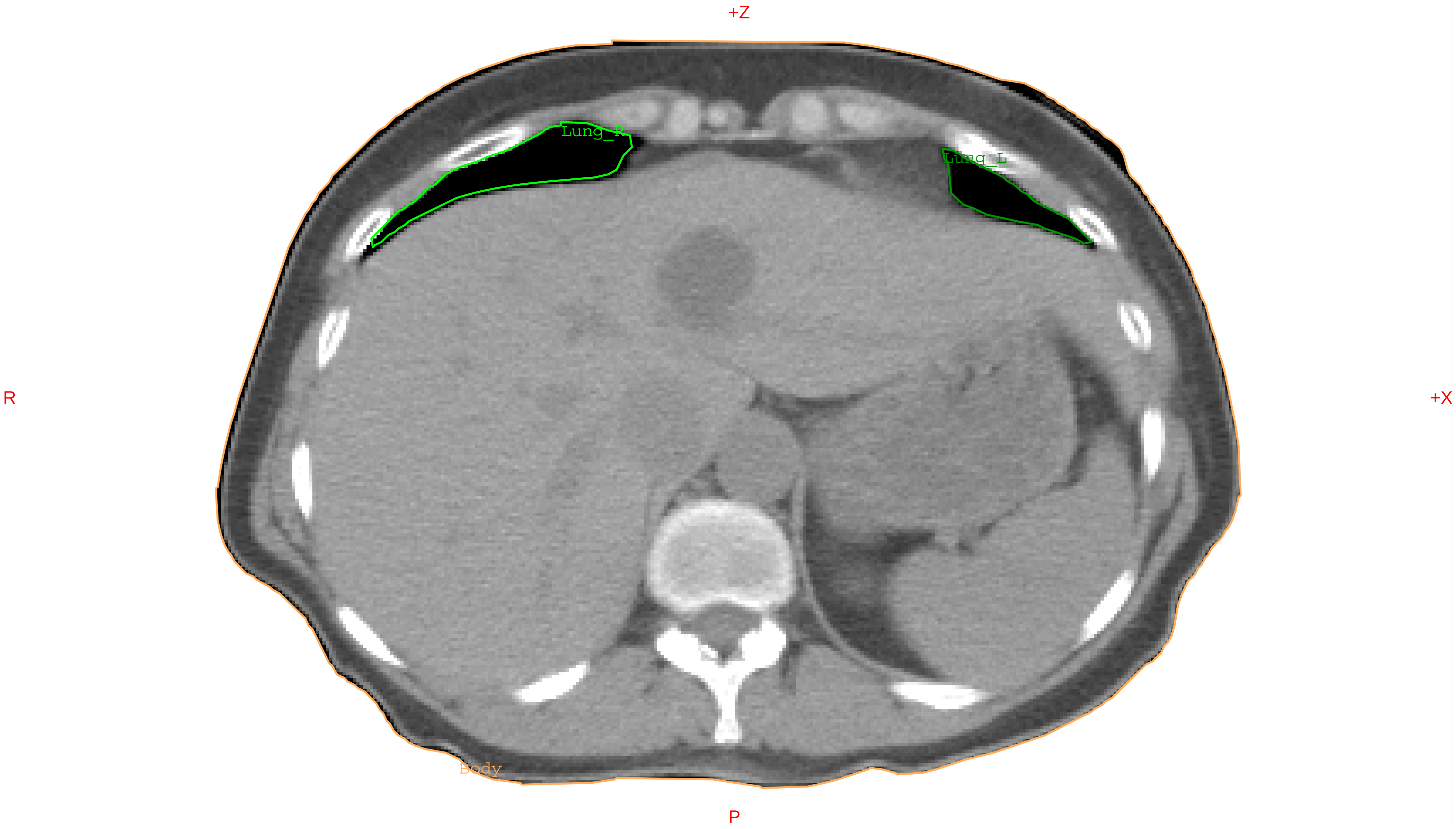

Scale 1:1.4 0 5 10 15 20 25 30 35 cm
